# An integrated cell atlas of the lung in health and disease

**DOI:** 10.1038/s41591-023-02327-2

**Published:** 2023-06-08

**Authors:** Lisa Sikkema, Ciro Ramírez-Suástegui, Daniel C. Strobl, Tessa E. Gillett, Luke Zappia, Elo Madissoon, Nikolay S. Markov, Laure-Emmanuelle Zaragosi, Yuge Ji, Meshal Ansari, Marie-Jeanne Arguel, Leonie Apperloo, Martin Banchero, Christophe Bécavin, Marijn Berg, Evgeny Chichelnitskiy, Mei-i Chung, Antoine Collin, Aurore C. A. Gay, Janine Gote-Schniering, Baharak Hooshiar Kashani, Kemal Inecik, Manu Jain, Theodore S. Kapellos, Tessa M. Kole, Sylvie Leroy, Christoph H. Mayr, Amanda J. Oliver, Michael von Papen, Lance Peter, Chase J. Taylor, Thomas Walzthoeni, Chuan Xu, Linh T. Bui, Carlo De Donno, Leander Dony, Alen Faiz, Minzhe Guo, Austin J. Gutierrez, Lukas Heumos, Ni Huang, Ignacio L. Ibarra, Nathan D. Jackson, Preetish Kadur Lakshminarasimha Murthy, Mohammad Lotfollahi, Tracy Tabib, Carlos Talavera-López, Kyle J. Travaglini, Anna Wilbrey-Clark, Kaylee B. Worlock, Masahiro Yoshida, Lung Biological Network Consortium, Yuexin Chen, James S. Hagood, Ahmed Agami, Peter Horvath, Joakim Lundeberg, Charles-Hugo Marquette, Gloria Pryhuber, Chistos Samakovlis, Xin Sun, Lorraine B. Ware, Kun Zhang, Maarten van den Berge, Yohan Bossé, Tushar J. Desai, Oliver Eickelberg, Naftali Kaminski, Mark A. Krasnow, Robert Lafyatis, Marko Z. Nikolic, Joseph E. Powell, Jayaraj Rajagopal, Mauricio Rojas, Orit Rozenblatt-Rosen, Max A. Seibold, Dean Sheppard, Douglas P. Shepherd, Don D. Sin, Wim Timens, Alexander M. Tsankov, Jeffrey Whitsett, Yan Xu, Nicholas E. Banovich, Pascal Barbry, Thu Elizabeth Duong, Christine S. Falk, Kerstin B. Meyer, Jonathan A. Kropski, Dana Pe’er, Herbert B. Schiller, Purushothama Rao Tata, Joachim L. Schultze, Sara A. Teichmann, Alexander V. Misharin, Martijn C. Nawijn, Malte D. Luecken, Fabian J. Theis

**Affiliations:** 1 Department of Computational Health, Institute of Computational Biology, Helmholtz Center Munich, Munich, Germany; 2 TUM School of Life Sciences, Technical University of Munich, Munich, Germany; 3 La Jolla Institute for Allergy and Immunology, La Jolla, CA USA; 4 Institute of Clinical Chemistry and Pathobiochemistry, TUM School of Medicine, Technical University of Munich, Munich, Germany; 5 Experimental Pulmonary and Inflammatory Research, Department of Pathology and Medical Biology, University Medical Centre Groningen, University of Groningen, Groningen, the Netherlands; 6 Groningen Research Institute for Asthma and COPD, University Medical Center Groningen, University of Groningen, Groningen, the Netherlands; 7 Department of Mathematics, Technical University of Munich, Garching, Germany; 8 Wellcome Sanger Institute, Hinxton, Cambridge UK; 9 Division of Pulmonary and Critical Care Medicine, Feinberg School of Medicine, Northwestern University, Chicago, IL USA; 10 Institut de Pharmacologie Moléculaire et Cellulaire, Université Côte d’Azur and Centre National de la Recherche Scientifique, Valbonne, France; 11 Institute of Lung Health and Immunity (a member of the German Center for Lung Research) and Comprehensive Pneumology Center with the CPC-M bioArchive, Helmholtz Center Munich, Munich, Germany; 12 Department of Pathology and Medical Biology, University Medical Center Groningen, University of Groningen, Groningen, the Netherlands; 13 Institute for Transplant Immunology, Hannover Medical School, Hannover, Germany; 14 Translational Genomics Research Institute, Phoenix, AZ USA; 15 3IA Côte d’Azur, Nice, France; 16 Department of Genomics and Immunoregulation, Life and Medical Sciences Institute, University of Bonn, Bonn, Germany; 17 Department of Pulmonary Diseases, University Medical Center Groningen, University of Groningen, Groningen, the Netherlands; 18 Pulmonology Department, Fédération Hospitalo-Universitaire OncoAge, Centre Hospitalier Universitaire de Nice, Université Côte d’Azur, Nice, France; 19 Research, Development and Innovation, Comma Soft, Bonn, Germany; 20 Division of Allergy, Pulmonary, and Critical Care Medicine, Department of Medicine, Vanderbilt University Medical Center, Nashville, TN USA; 21 Core Facility Genomics, Helmholtz Center Munich, Munich, Germany; 22 Department of Translational Psychiatry, Max Planck Institute of Psychiatry and International Max Planck Research School for Translational Psychiatry, Munich, Germany; 23 School of Life Sciences, Respiratory Bioinformatics and Molecular Biology, University of Technology Sydney, Sydney, Australia; 24 Division of Neonatology and Pulmonary Biology, Cincinnati Children’s Hospital Medical Center, University of Cincinnati College of Medicine, Cincinnati, OH USA; 25 Department of Pediatrics, University of Cincinnati College of Medicine, Cincinnati, OH US; 26 Center for Genes, Environment, and Health, National Jewish Health, Denver, CO USA; 27 Department of Cell Biology, Duke University School of Medicine, Durham, NC USA; 28 Department of Pharmacology and Regenerative Medicine, University of Illinois Chicago, Chicago, IL USA; 29 Division of Rheumatology and Clinical Immunology, Department of Medicine, University of Pittsburgh, Pittsburgh, PA USA; 30 Division of Infectious Diseases and Tropical Medicine, Klinikum der Lüdwig-Maximilians-Universität, Munich, Germany; 31 Department of Biochemistry, Stanford University School of Medicine, Stanford, CA USA; 32 Howard Hughes Medical Institute, Chevy Chase, MD USA; 33 Allen Institute for Brain Science, Seattle, WA USA; 34 Department of Respiratory Medicine, Division of Medicine, University College London, London, UK; 35 Institut Universitaire de Cardiologie et de Pneumologie de Québec, Department of Molecular Medicine, Laval University, Quebec City, Quebec Canada; 36 Department of Medicine, Stanford University School of Medicine, Stanford, CA USA; 37 Division of Pulmonary, Allergy, and Critical Care Medicine, University of Pittsburgh, Pittsburgh, PA USA; 38 Pulmonary, Critical Care and Sleep Medicine, Yale School of Medicine, New Haven, CT USA; 39 Garvan Institute of Medical Research, Sydney, New South Wales Australia; 40 Cellular Genomics Futures Institute, University of New South Wales, Sydney, New South Wales Australia; 41 Center for Regenerative Medicine, Massachusetts General Hospital, Harvard Medical School, Cambridge, MA USA; 42 Department of Internal Medicine, Division of Pulmonary, Critical Care and Sleep Medicine, The Ohio State University, Columbus, OH USA; 43 Klarman Cell Observatory, Broad Institute of Harvard and MIT, Cambridge, MA USA; 44 Cellular and Tissue Genomics, Genentech, South San Francisco, CA USA; 45 Department of Pediatrics, National Jewish Health, Denver, CO USA; 46 Division of Pulmonary Sciences and Critical Care Medicine, University of Colorado School of Medicine, Aurora, CO USA; 47 Division of Pulmonary, Critical Care, Allergy and Sleep Medicine, University of California, San Francisco, San Francisco, CA USA; 48 Department of Physics and Center for Biological Physics, Arizona State University, Tempe, AZ USA; 49 Centre for Heart Lung Innovation, St. Paul’s Hospital, University of British Columbia, Vancouver, British Columbia Canada; 50 Department of Genetics and Genomic Sciences, Icahn School of Medicine at Mount Sinai, New York, NY USA; 51 Department of Pediatrics, Division of Respiratory Medicine, University of California, San Diego, La Jolla, CA USA; 52 Department of Cell and Developmental Biology, Vanderbilt University, Nashville, TN USA; 53 Computational and Systems Biology Program, Sloan Kettering Institute, Memorial Sloan Kettering Cancer Center, New York, NY USA; 54 PRECISE Platform for Single Cell Genomics and Epigenomics, Deutsches Zentrum für Neurodegenerative Erkrankungen and University of Bonn, Bonn, Germany; 55 Department of Physics, Cavendish Laboratory, University of Cambridge, Cambridge, UK; 56 Department of Pediatrics (Pulmonology), University of North Carolina at Chapel Hill, Chapel Hill, NC USA; 57 Biological Research Centre, Szeged, Hungary; 58 Institute for Molecular Medicine Finland, University of Helsinki, Helsinki, Finland; 59 Department of Gene Technology, KTH Royal Institute of Technology, Stockholm, Sweden; 60 Department of Pediatrics, University of Rochester Medical Center, Rochester, NY USA; 61 Lab and Department of Molecular Bioscience, Stockholm University, Stockholm, Sweden; 62 University of California, San Diego, La Jolla, CA USA

**Keywords:** Cell biology, Data integration, Transcriptomics, Mechanisms of disease, Computational models

## Abstract

Single-cell technologies have transformed our understanding of human tissues. Yet, studies typically capture only a limited number of donors and disagree on cell type definitions. Integrating many single-cell datasets can address these limitations of individual studies and capture the variability present in the population. Here we present the integrated Human Lung Cell Atlas (HLCA), combining 49 datasets of the human respiratory system into a single atlas spanning over 2.4 million cells from 486 individuals. The HLCA presents a consensus cell type re-annotation with matching marker genes, including annotations of rare and previously undescribed cell types. Leveraging the number and diversity of individuals in the HLCA, we identify gene modules that are associated with demographic covariates such as age, sex and body mass index, as well as gene modules changing expression along the proximal-to-distal axis of the bronchial tree. Mapping new data to the HLCA enables rapid data annotation and interpretation. Using the HLCA as a reference for the study of disease, we identify shared cell states across multiple lung diseases, including *SPP1*^+^ profibrotic monocyte-derived macrophages in COVID-19, pulmonary fibrosis and lung carcinoma. Overall, the HLCA serves as an example for the development and use of large-scale, cross-dataset organ atlases within the Human Cell Atlas.

## Abstract (web summary)

A single-cell atlas of the human lungs, integrating data from 2.4 million cells from 486 individuals and including samples from healthy and diseased lungs, provides a roadmap for the generation of organ-scale cell atlases.

## Main

Rapid technological improvements over the past decade have allowed single-cell datasets to grow both in size and number^1^. This has led consortia, such as the Human Cell Atlas, to pursue the generation of large-scale reference atlases of human organs^2,3^. To advance our understanding of health and disease, such atlases must capture variation between individuals that is expected to impact the molecular phenotypes of the cells in a tissue. Whereas the generation of atlases at this scale by single research groups is currently not feasible, integrating datasets generated by the research community at large will enable capture of the diversity of the cellular landscape across individuals.

Several foundational studies have started to map the cellular landscape of the healthy human lung^4–6^. These studies each have a specific bias due to their choice of experimental protocol and technologies, and are therefore not tailored to serve as a universal reference. The studies moreover include only a limited number of samples and individuals, thus lacking the scale and diversity to capture the full cellular heterogeneity present within the lung as well as across individuals.

Integrated single-cell atlases provide novel insights not obtained in individual studies. Recent reference atlases have led to the discovery of unknown cell types^7–9^, the identification of marker genes that are reproducible across studies^7,10,11^, the comparison of animal and in vitro models with human healthy and diseased tissue^7,12,13^ and patient stratification for disease endotypes^14,15^. However, many currently available integrated atlases are limited in the number of human samples^7,8,10–12,16^, datasets^16^ or cell types^7,9,12,17,18^ per organ, as well as donor metadata^12,13,17,19,20^ (for example, age, body mass index (BMI) and smoking status), or focus mainly on a specific disease^14,15,17^. These limitations constrain the potential of atlases to serve as a reference, as they fail to represent and catalog the diversity of cellular phenotypes within the healthy organ and across individuals. Moreover, when integrating data from different sources, it is paramount to correctly separate technical biases from biologically relevant information. Yet, the majority of existing atlases have not assessed the quality of their data integration. Nonetheless, successful integration of the available datasets into a single tissue atlas is a critical step in achieving the goals of the Human Cell Atlas^2^.

**Fig. 1:**
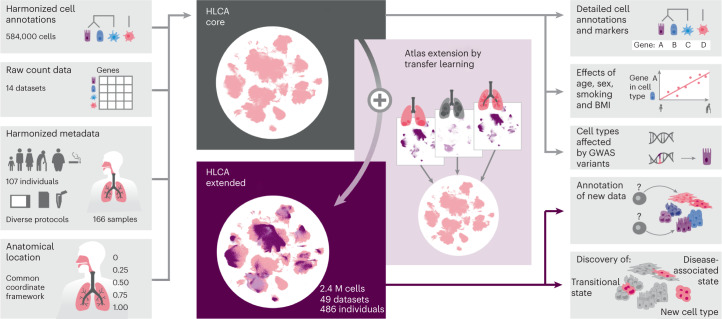
HLCA study overview. Harmonized cell annotations, raw count data, harmonized patient and sample metadata and sample anatomical locations encoded into a CCF were collected and generated as input for the HLCA core (left). After integration of the core datasets, the atlas was extended by mapping 35 additional datasets, including disease samples, to the HLCA core, bringing the total number of cells in the extended HLCA to 2.4 million (M). The HLCA core provides detailed consensus cell annotations with matched consensus cell type markers (top right), gene modules associated with technical, demographic and anatomical covariates in various cell types (middle right), GWAS-based association of lung conditions with cell types (middle right) and a reference projection model to annotate new data (middle right) and discover previously undescribed cell types, transitional cell states and disease-associated cell states (right, bottom).

In this resource, we present an integrated single-cell transcriptomic atlas of the human respiratory system, including the upper and lower airways, from published and newly generated datasets (Fig. 1). The Human Lung Cell Atlas (HLCA) comprises data from 486 donors and 49 datasets, including 2.4 million cells, which we re-annotated to generate a consensus cell type reference. The HLCA expands our understanding of the healthy lung and its changes in disease and can be used as a reference for analyzing future lung data. Together, we provide a roadmap for building and using comprehensive, interpretable and up-to-date organ- and population-scale cell atlases.

## Results

### Data integration establishes the HLCA core

**Fig. 2:**
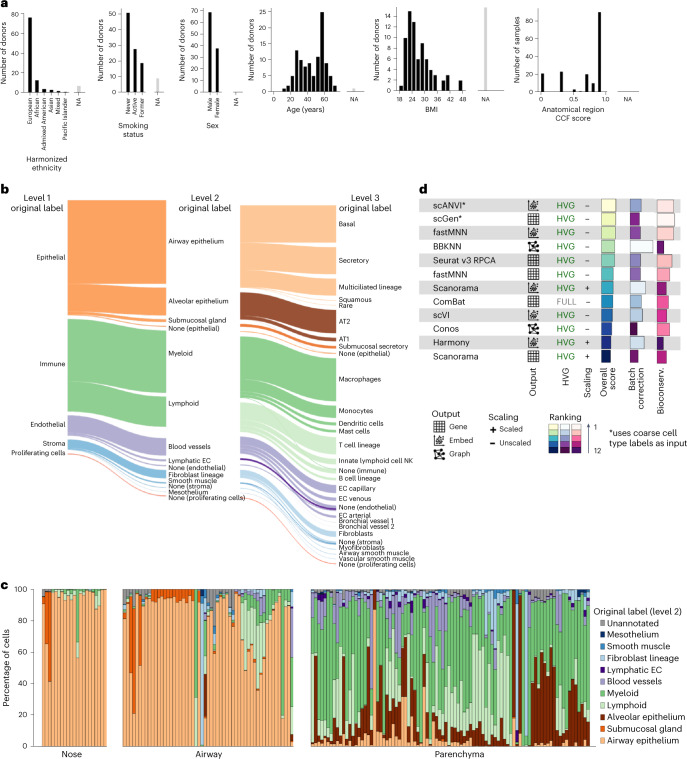
Composition and construction of the HLCA core. **a**, Donor and sample composition in the HLCA core for demographic and anatomical variables. Donors/samples without annotation are shown as not available (NA; gray bars) for each variable. For the anatomical region CCF score, 0 represents the most proximal part of the lung and airways (nose) and 1 represents the most distal (distal parenchyma). Donors show diversity in ethnicity (harmonized metadata proportions: 65% European, 14% African, 2% admixed American, 2% mixed, 2% Asian, 0.4% Pacific Islander and 14% unannotated; see Methods), smoking status (52% never, 16% former, 15% active and 17% NA), sex (60% male and 40% female), age (ranging from 10–76 years) and BMI (20–49; 30% NA). **b**, Overview of the HLCA core cell type composition for the first three levels of cell annotation, based on harmonized original labels. In the cell type hierarchy, the lowest level (1) consists of the coarsest possible annotations (that is, epithelial (48% of cells), immune (38%), endothelial (9%) and stromal (4%)). Higher levels (2–5) recursively break up coarser-level labels into finer ones (Methods). Cells were set to ‘none’ if no cell type label was available at the level. Cell labels making up less than 0.02% of all cells are not shown. Overall, 94, 66 and 7% of cells were annotated at levels 3, 4 and 5, respectively. **c**, Cell type composition per sample, based on level 2 labels. Samples are ordered by anatomical region CCF score. **d**, Summary of the dataset integration benchmarking results. Batch correction score and biological conservation score each show the mean across metrics of that type, as shown in Supplementary Fig. 1, with metric scores scaled to range from 0 to 1. Both Scanorama and fastMNN were benchmarked on two distinct outputs: the integrated gene expression matrix and integrated embedding (see output). The methods are ordered by overall score. For each method, the results are shown only for their best-performing data preprocessing. Methods marked with an asterisk use coarse cell type labels as input. Preprocessing is specified under HVG (that is, whether or not genes were subsetted to the 2,000 (HVG) or 6,000 (FULL) most highly variable genes before integration) and scaling (whether genes were left unscaled or scaled to have a mean of 0 and a standard deviation of 1 across all cells). EC, endothelial cell; NK, natural killer; Bioconserv., conservation of biological signal.

To build the HLCA, we collected single-cell RNA sequencing (scRNA-seq) data and detailed, harmonized technical, biological and demographic metadata from 14 datasets (11 published and three unpublished)^4–6,21–25,26,27^. These datasets include samples from 107 individuals, with diversity in age, sex, ethnicity (harmonized as detailed in Methods), BMI and smoking status (Fig. 2a). Cells were obtained from 166 tissue samples using a variety of tissue donors, sampling methods, experimental protocols and sequencing platforms (Supplementary Tables 1 and 2). Anatomical locations of the samples were projected onto a one-dimensional (1D) common coordinate framework (CCF), representing the proximal (0) to distal (1) axis of the respiratory system, to standardize the anatomical location of origin (Fig. 2a and Supplementary Tables 2 and 3).

Consensus definitions of cell types based on single-cell transcriptomic data across studies—particularly of transitional cell states—are lacking. To enable supervised data integration and downstream integrated analysis, we harmonized cell type nomenclature by building a five-level hierarchical cell identity reference framework (Methods, Supplementary Table 4 and Fig. 2b). We then unified cell type labeling across datasets by mapping the collected cell identity labels for every dataset as provided by the data generator to the hierarchical reference framework, showing varying cell type proportions per sample (Fig. 2c).

**Fig. 3:**
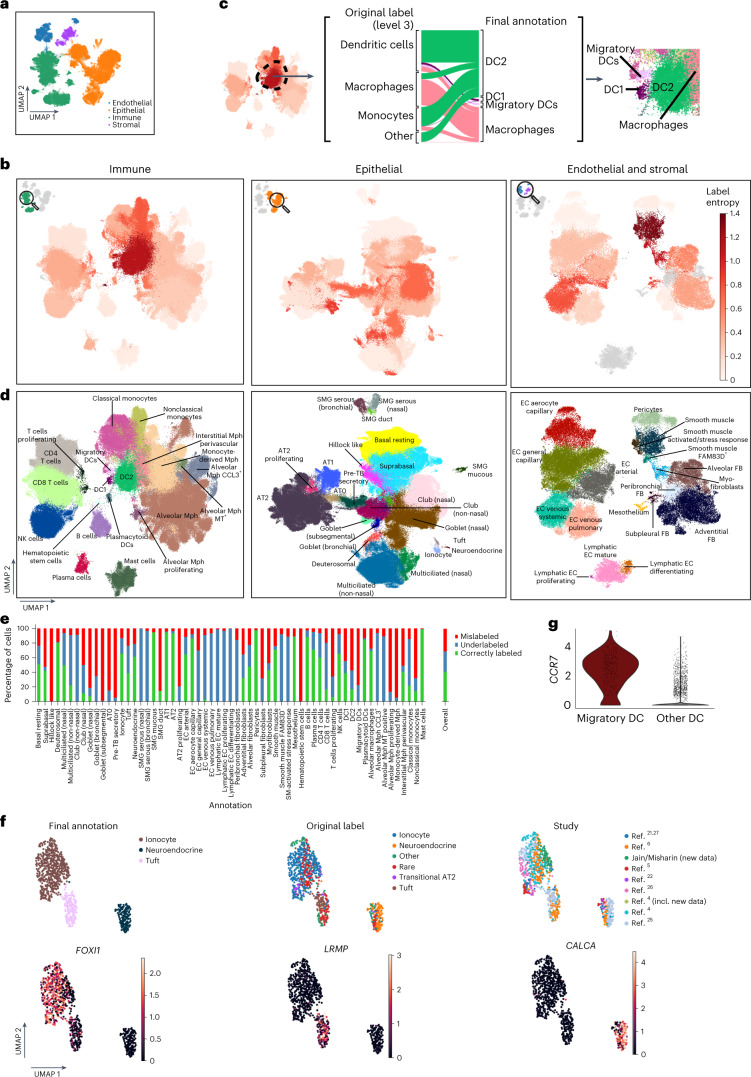
The HLCA core conserves detailed biology and enables consensus-driven annotation. **a**, A UMAP of the integrated HLCA, colored by level 1 annotation. **b**, Cluster label disagreement (label entropy) of Leiden 3 clusters of the HLCA. The HLCA was split into three parts (immune, epithelial and endothelial/stromal) for ease of visualization. Cells from every cluster are colored by label entropy. Clusters with less than 20% of cells annotated at level 3 are colored gray. **c**, Cell type label composition of the immune cluster with the most label disagreement (left), with original labels (middle left) and matching manual re-annotations (middle right). A zoom-in on the UMAP from **b** shows the final re-annotations (right). **d**, UMAPs of the immune, epithelial and endothelial/stromal parts of the HLCA core with cell annotations from the expert manual re-annotation. **e**, Percentage of cells originally labeled correctly, mislabeled or underlabeled (that is, only labeled at a coarser level) compared with final manual re-annotations. The percentages were calculated per manual annotation, as well as across all cells (right bar). **f**, UMAP of HLCA clusters annotated as rare epithelial cell types (that is, ionocytes, neuroendocrine cells and tuft cells). Final annotations, original labels and the study of origin are shown (top), as well as the expression of ionocyte marker *FOXI1*, tuft cell marker *LRMP* and neuroendocrine marker *CALCA* (bottom). **g**, Log-normalized expression of the migratory dendritic cell marker *CCR7* in cells identified during re-annotation as migratory dendritic cells, versus other dendritic cells. AT, alveolar type; DC, dendritic cell; FB, fibroblast; Mph, macrophage; MT, metallothionein; SM, smooth muscle; SMG, submucosal gland; TB, terminal bronchiole.

To optimally remove dataset-specific batch effects, we evaluated 12 different data integration methods on 12 datasets^4–6,21–25^ (Fig. 2d and Supplementary Fig. 1) using our previously established benchmarking pipeline^28^. We used the top-performing integration method, scANVI, to create an integrated embedding of all 584,444 cells of 107 individuals from the collected datasets: the HLCA core (Fig. 3a).

### Consensus cell type annotations based on the HLCA core

**Extended Data Fig. 1:**
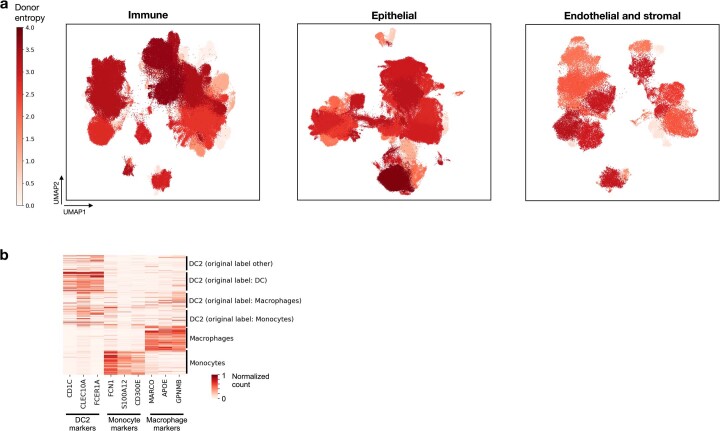
HLCA cluster donor diversity and marker expression for a cluster with high cell type label disagreement. **a**, Donor diversity is calculated for every cluster as entropy of donor proportions in the cluster, with high entropy indicating the cluster contains cells from many different donors. Most clusters (80 out of 94) contain cells from many donors (median 47 donors per cluster, range 2–102), as illustrated by high donor entropy (>0.43), whereas 14 clusters show low donor diversity. These are largely immune cell clusters (n=13, of which 7 macrophage clusters, 4 T cell clusters and 2 mast cell clusters), representing donor- or group-specific phenotypes. Matching cell type annotations are shown in Fig. 3d. **b**, Marker expression among cells from the immune cluster with highest disagreement in original cell type labels (high ‘label entropy’). DC2, monocyte and macrophage marker expression is shown for cells from Fig. 3c. Cells are labeled by their final annotation, as well as their original label. Log-normalized counts are scaled such that for each gene the 99th expression percentile, as calculated among all cells included in the heatmap, is set to 1. DC: dendritic cell.

A large-scale integrated atlas provides the unique opportunity to systematically investigate the consensus in cell type labeling across datasets. To identify areas of consensus and disagreement, we iteratively clustered the HLCA core and investigated donor diversity and cell type label agreement in these clusters using entropy scores (see Methods). Most clusters contained cells from many donors (Extended Data Fig. 1a). Clusters with low donor diversity (*n* = 14) were largely immune cell clusters (*n* = 13), representing donor- or donor group-specific phenotypes. Similarly, a high diversity of (contradictory) cell type labels (high label entropy) can identify both annotation disagreements between studies and clusters of doublets (Methods). Most clusters (61 out of 94) showed low label entropy, suggesting overall agreement of coarse cell type labels across datasets (Fig. 3b). The remaining 33 clusters exhibited high label entropy, highlighting cellular phenotypes that were differently labeled across datasets (Fig. 3b). For example, the immune cluster with the highest label entropy contained many cells that were originally mislabeled as monocytes and macrophages but were actually type 2 dendritic cells (Fig. 3c and Extended Data Fig. 1b). Thus, populations with high label entropy identify mislabeled cell types, indicating the need for consensus re-annotation of the integrated atlas.

**Extended Data Fig. 2:**
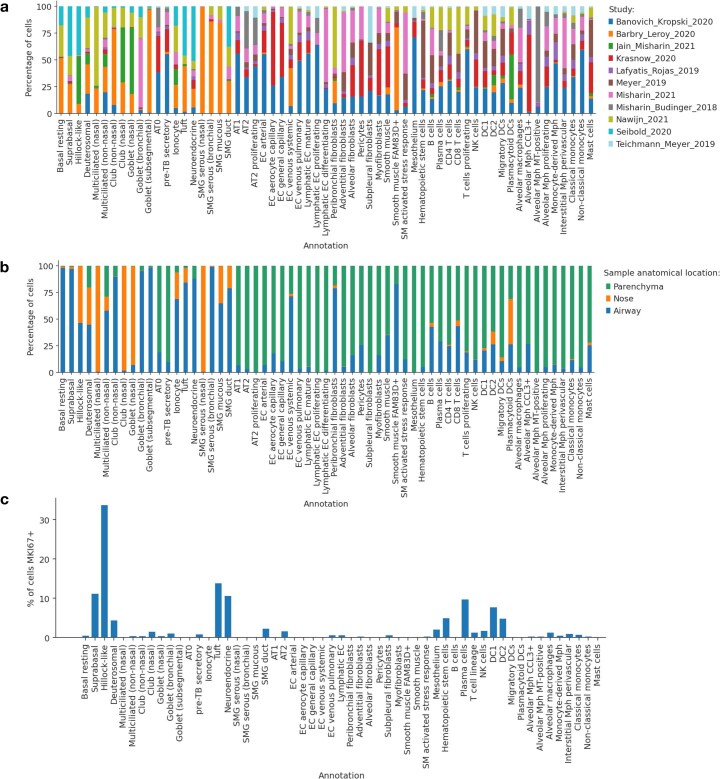
HLCA core cell type composition details. **a**, Percentage of cells from each of the 11 studies included in the HLCA core, shown per cell type (3 studies include 2 separate datasets). Each cell type was detected in at least 4 out of 14 datasets, with a median of 11 datasets in which a cell type was detected, and a maximum of 14. **b**, Percentage of cells from each of the three anatomical locations, shown per cell type. **c**, Percentage of cells with at least one UMI count for *MKI67*, a marker gene of proliferating cells, shown per cell type. AT: alveolar type. TB: terminal bronchiole. SMG: submucosal gland. DC: dendritic cell. Mph: macrophage. NK: natural killer. MT: metallothionein. SM: smooth muscle. EC: endothelial cell.

**Extended Data Fig. 3:**
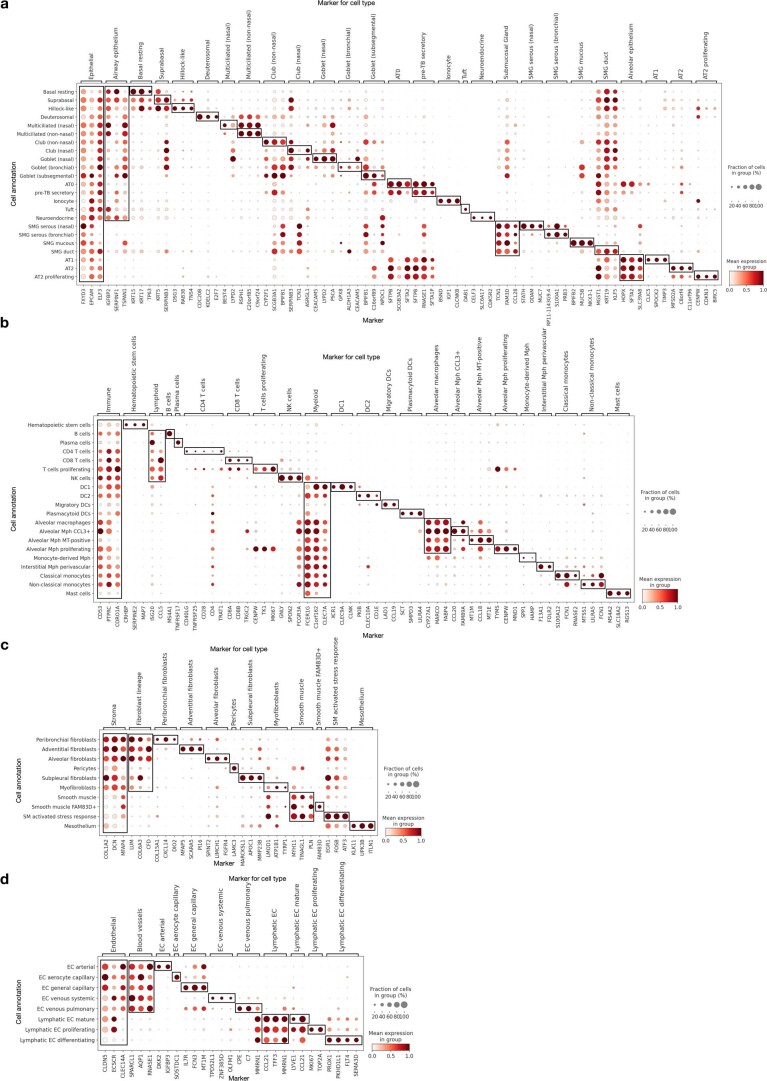
Marker gene expression for all 61 cell types in the HLCA core. Expression is shown within each cell type compartment. **a**, Epithelial cell type markers, **b**, Immune cell type markers, **c**, Stromal cell type markers, **d**, Endothelial cell type markers. Expression was normalized such that the maximum group expression of cells within the compartment for each marker was set to 1. Marker gene sets include both sets that mark groups of cell types (for example ‘epithelial’) and single cell types (for example ‘basal resting’). For each marker gene set, cell types identified by the set are boxed. AT: alveolar type. TB: terminal bronchiole. SMG: submucosal gland. DC: dendritic cell. Mph: macrophage. NK: natural killer. MT: metallothionein. SM: smooth muscle. EC: endothelial cell.

As a first step to achieve such a consensus on the diversity of cell types present in the HLCA core, we performed a full re-annotation of the integrated data on the basis of the original annotations and six expert opinions (consensus annotation; Methods and Fig. 3d). Each of the 61 annotated cell types (Supplementary Table 5) was detected in at least four datasets out of 14, often in specific parts of the respiratory system, and different cell types showed varying fractions of proliferating (*MKI67*^+^) cells (Extended Data Fig. 2a–c). While our consensus cell type annotations partly correspond to original labels (41% of cells), there were also refinements (28%) and substantial re-annotations (31%; Fig. 3e and Supplementary Fig. 2). To robustly characterize the cell types, we established a universal set of marker genes that generalizes across individuals and studies (Methods, Extended Data Fig. 3 and Supplementary Table 6). The fully re-annotated HLCA core thus combines data from a diverse set of studies to provide a carefully curated reference for cell type annotations and marker genes in healthy lung tissue.

### The HLCA recovers rare cell types and identifies novel ones

Rare cell types, such as ionocytes, tuft cells, neuroendocrine cells and specific immune cell subsets, are often difficult to identify in individual datasets. Yet, combining datasets in the HLCA core provides better power for identifying these rare cell types. Ionocytes, tuft and neuroendocrine cells make up only 0.08, 0.01 and 0.02% of the cells in the HLCA core according to the original labels, and were originally identified in only seven, two and four datasets out of 14, respectively. Despite their low abundance, these cells formed three separate clusters of the HLCA core (Fig. 3f). Our re-annotation increases the number of datasets in which these cells are detected up to threefold and identifies both cells falsely annotated as monocytes, tuft cells or neuroendocrine cells, as well as originally undetected rare cells (Fig. 3f and Supplementary Fig. 3a). Importantly, other integration methods tested during our benchmarking, such as Harmony^29^ and Seurat’s RPCA^30^, failed to separate these rare cells into distinct clusters (Supplementary Fig. 3b).

**Extended Data Fig. 4:**
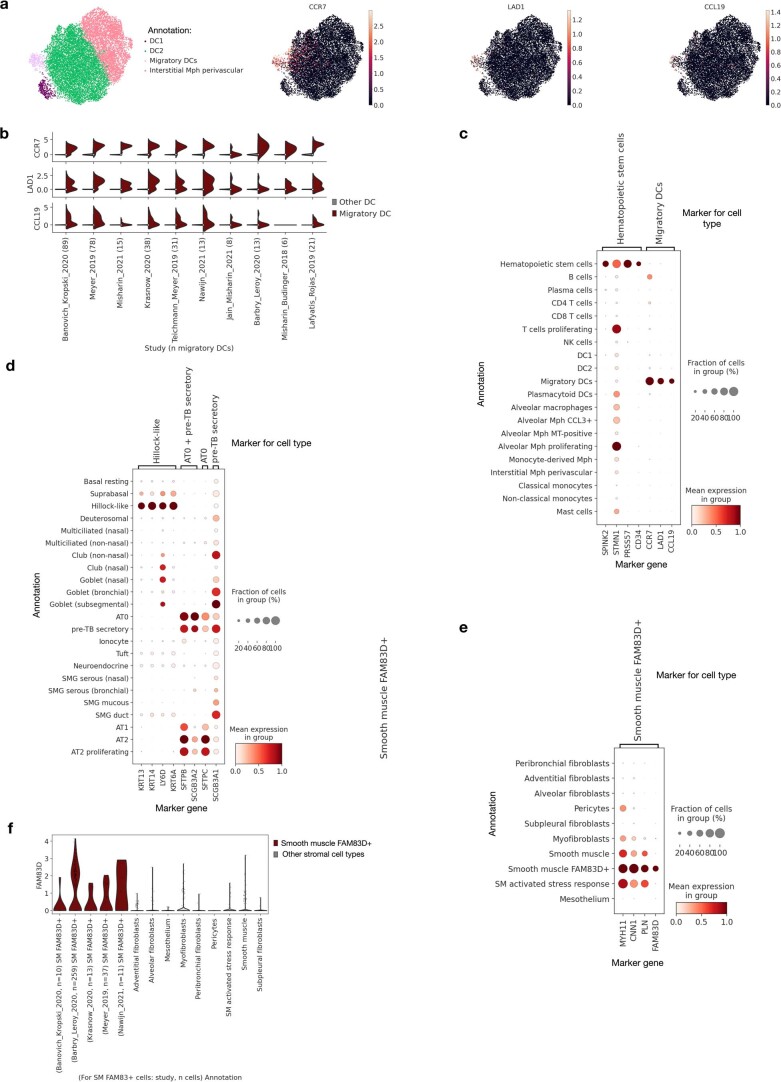
Marker expression of several rare and novel cell types detected in the HLCA. **a**, A UMAP embedding of all cells annotated as dendritic cells, colored by final detailed annotation (left), and by expression of three migratory DC marker genes (right, *CCR7*, *LAD1*, and *CCL19*). **b**, Expression of migratory DC marker genes from **a** among migratory DCs (red, right half of violins) versus other DCs (gray, left half of violins), split by study. Number of migratory DCs per study is specified in the x-axis labels. **c**, Expression of markers for two novel immune cell types (hematopoietic stem cells and migratory DCs, found in 9 and 10 out of 11 studies, respectively), shown per stromal cell type. **d**, Expression of markers for three novel epithelial cell types (hillock-like, AT0, and pre-TB secretory cells, found in 9, 9, and 11 out of 11 studies, respectively), shown per epithelial cell type. Two markers shared between AT0 and pre-TB secretory cells are also included. **e**, Expression of markers for a novel stromal cell type (‘smooth muscle FAM83D+’, found in 8 out of 11 studies), including three general smooth muscle marker genes and one marker gene uniquely expressed in FAM83D+ smooth muscle cells (*FAM83D*), shown per stromal cell type. For **c**-**e**, gene counts were normalized such that the maximum expression of a group of cells in the plot was set to 1. **f**, *FAM83D* expression across stromal cell types. Cells annotated as FAM83D+ smooth muscle are split by study. Studies with fewer than 3 smooth muscle FAM83D+ cells are not shown. DC: dendritic cell. Mph: macrophage. MT: metallothionein. AT: alveolar type. SMG: submucosal gland. TB: terminal bronchiole.

We were further able to detect six cell identities that were not previously found in the human lung or were only recently described in individual studies. These cell types include migratory dendritic cells^31,32^ (*n* = 312 cells, expressing *CCR7*, *LAD1* and *COL19*), hematopoietic stem cells (*n* = 60, expressing *SPINK2*, *STMN*, *PRSS57* and *CD34*), highly proliferative hillock-like epithelial cells not previously reported in adult human lung (*n* = 4,600, expressing *KRT6A*, *KRT13* and *KRT14*), the recently described alveolar type 0 cells (*n* = 1,440, expressing *STFPB*^+^, *SCGB3A2*^+^, *SFTPC*^high^ and *SCGB3A1*^low^) and the closely related preterminal bronchiole secretory cells (*n* = 4,393, expressing *SFTPB*^+^, *SCGB3A2*^+^, *SFTPC*^low^ and *SCGB3A1*^high^, together with alveolar type 0 cells called transitional club-AT2 cells)^33,34^ and a subset of smooth muscle cells (*n* = 335) that to our knowledge have not previously been described (Fig. 3d,g and Extended Data Fig. 4a–f). These smooth muscle cells, predominantly found in the airways, express canonical smooth muscle markers (*CNN1* and *MYH11*) and also uniquely and consistently express *FAM83D* across datasets (Extended Data Fig. 4e,f). The HLCA core thus enables improved detection and identification of rare cell types, as well as the discovery of unknown cell types.

### Donor and experimental factors affect gene expression profiles

**Fig. 4:**
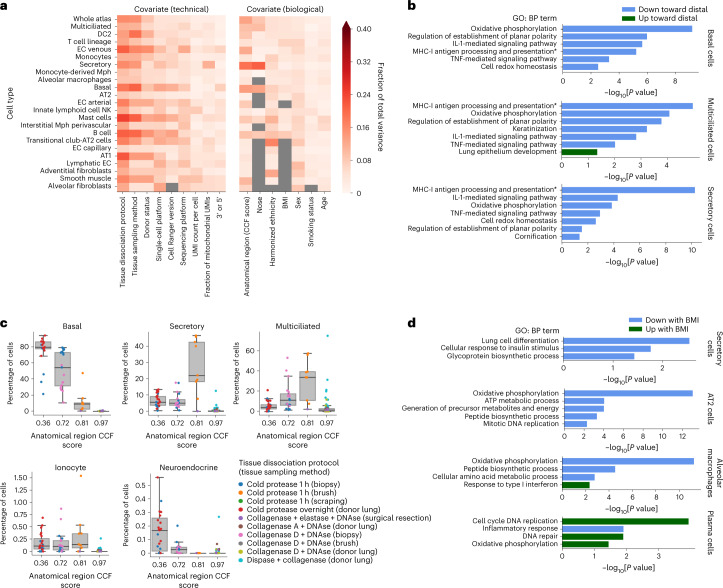
Demographic and technical variables driving interindividual variation. **a**, Fraction of total inter-sample variance in the HLCA core integrated embedding that correlates with specific covariates. Covariates are split into technical (left) and biological covariates (right). Cell types are ordered by the number of samples in which they were detected. Only cell types present in at least 40 samples are shown. Tissue sampling method represents the way a sample was obtained (for example, surgical resection or nasal brush). Donor status represents the state of the donor at the moment of sample collection (for example, organ donor, diseased alive or healthy alive). The heatmap is masked gray where fewer than 40 samples were annotated for a specific covariate or where only one value was observed for all samples for that cell type. **b**, Selection of gene sets that are significantly associated with anatomical location CCF score, in different airway epithelial cell types. All gene set names are Gene Ontology biological process (GO: BP) terms. Sets upregulated toward distal lungs are shown in green, whereas sets downregulated are shown in blue. The full name of the term marked by an asterisk is ‘Antigen processing and presentation of exogenous peptide antigen via MHC-I’. **c**, Cell type proportions per sample, along the proximal-to-distal axis of the respiratory system. The lowest and highest CCF scores shown (0.36 and 0.97) represent the most proximal and most distal sampled parts of the respiratory system, respectively (trachea and parenchyma), excluding the upper airways. The dots are colored by the tissue dissociation protocol and tissue sampling method used for each sample. The boxes show the median and interquartile range of the proportions. Samples with proportions more than 1.5 times the interquartile range away from the high and low quartile are considered outliers. Whiskers extend to the furthest nonoutlier point. *n* = 23, 19, 9 and 90 for CCF scores 0.36, 0.72, 0.81 and 0.97, respectively. **d**, Selection of gene sets significantly up- (green) or downregulated (blue) with increasing BMI, in four different cell types. For **b** and **d**, *P* values were calculated using correlation-adjusted mean-rank gene set tests (Methods) and false discovery rate corrected using the Benjamini–Hochberg procedure. IL-1, interleukin-1; MHC-I, major histocompatibility complex class I; TNF, tumor necrosis factor.

Demographic and other metadata covariates affect cellular transcriptional phenotypes^19,25^. Better insight into the impact of these covariates (for example, sex, BMI and smoking) on cell type gene expression can shed light on the contribution of these factors to progression from healthy to diseased states. In addition, technical covariates such as ribosomal and mitochondrial genes exhibit batch-specific variation in expression (Methods and Supplementary Table 7). The diversity in demographics (for example, smoking status, age, harmonized ethnicity and BMI) and experimental protocols represented in the HLCA core enables us to explore the contribution of each technical or biological covariate to cell type-specific gene expression variation (Methods and Supplementary Fig. 4). For many cell types, anatomical location is the biological variable explaining most of the variance between samples (Fig. 4a). Furthermore, sex is most associated with transcriptomic variation in lymphatic endothelial cells, whereas BMI is most associated with variation in B and T cells, harmonized ethnicity in transitional club-AT2 cells and smoking status in innate lymphoid/natural killer cells. Furthermore, for several cell types (for example, mast, AT1 and smooth muscle cells), the tissue dissociation protocol explains most of the variance of all technical as well as biological covariates recorded. These associations provide a systematic overview of the effects of biological and technical factors on the transcriptional state of lung cell types.

To better characterize how biological variables affect cellular phenotypes, we modeled their cell type-specific effects on the transcriptome at the gene level (Methods). Sex-related differences in lymphatic endothelial cells are dominated by differential expression of genes located on the X and Y chromosomes, but also include a decrease in IFNAR1 in females (Supplementary Table 8), which may be linked to differential interferon responses between the biological sexes^35^. We furthermore found cell type-specific programs that change with proximal (low CCF score) to distal (high CCF score) location along the respiratory tract (Supplementary Tables 8 and 9). For instance, oxidative phosphorylation (including cytochrome c oxidase genes such as *COX7A1*), antigen presentation by major histocompatibility complex class I molecules (including proteasome and protease subunit genes such as *PSMD14* and *PSMB4*), signaling by interleukin-1 and tumor necrosis factor α, as well as planar cell polarity, were downregulated toward more distal locations in secretory, multiciliated and basal cells (Fig. 4b). Some gene programs were specific for a subset of airway epithelial cell types (for example, cornification and keratinization, which were programs that were downregulated in distal multiciliated and secretory cells; including genes such as *KRT8* and *KRT19*). The changes in airway epithelial cell states toward the terminal airways are further illustrated by increased expression of developmental pathway genes such as *NKX2-1*, *NFIB*, *GATA6*, *BMP4* and *SOX9* in multiciliated cells along the proximal-to-distal axis (Fig. 4b), whereas basal cells decrease in number (Fig. 4c)^36^. Similarly, several cell types display transcriptomic changes in donors with increasing BMI (Fig. 4d and Supplementary Tables 8 and 9). AT2 cells, secretory cells and alveolar macrophages exhibit downregulation of a range of biological processes (Supplementary Fig. 5), including cellular respiration, differentiation and synthesis of peptides and other molecules. In secretory cells, a downregulation of the insulin response pathway is also associated with higher BMI, in line with the insulin resistance observed in donors with obesity^37,38^. In alveolar macrophages, inflammatory responses involving JAK/STAT signaling (previously associated with obesity-induced chronic systemic inflammation^38^) are associated with higher BMI. In contrast, in plasma cells, high BMI is associated with downregulation of gene sets associated with immune response and upregulation of gene sets associated with cellular respiration, the cell cycle and DNA repair. This is consistent with obesity being a known risk factor for multiple myeloma—a plasma cell malignancy^39^. Thus, the HLCA enables a detailed understanding of the effects of anatomical and demographic covariates on the cellular landscape of the lung and their relation to disease.

**Extended Data Fig. 5:**
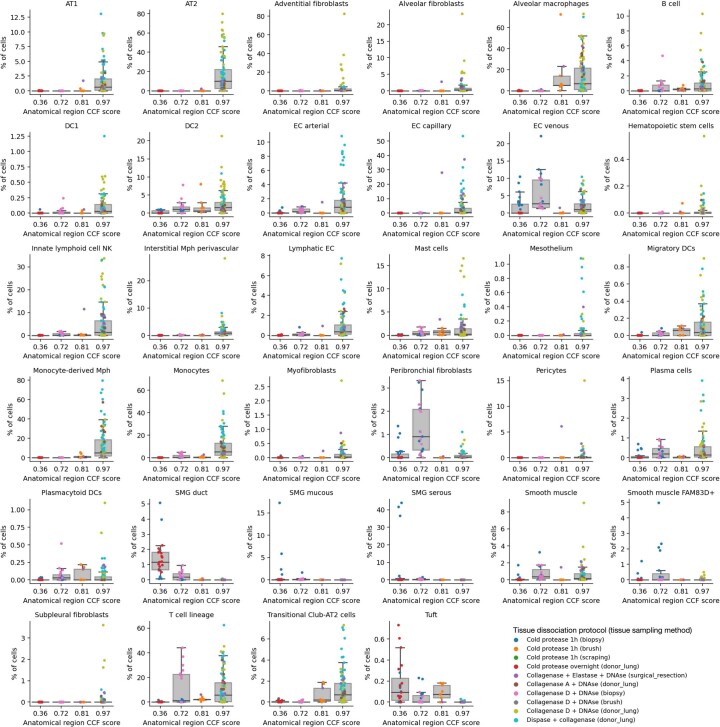
Cell type proportions per sample along the proximal-to-distal axis of the lung. All cell types not included in Fig. 4b are shown. The lowest and highest CCF score shown (0.36, 0.97) represent the most proximal and most distal sampled parts of the respiratory system, respectively (trachea and parenchyma), excluding the upper airways. Dots are colored by the tissue dissociation protocol and tissue sampling method used for the sample. Boxes show median and interquartile range of the proportions. Samples with proportions more than 1.5 times the interquartile range away from the high and low quartile are considered outliers. Whiskers extend to the furthest non-outlier point. n=23, 19, 9 and 90 for CCF score 0.36, 0.72, 0.81 and 0.97, respectively. AT: alveolar type. DC: dendritic cell. EC: endothelial cell. NK: natural killer. Mph: macrophages. SMG: submucosal gland.

Biological and technical factors can also affect cell type proportions. Indeed, all cell types show changes in abundance as a function of anatomical location **(**Fig. 4c and Extended Data Fig. 5). For example, ionocytes are present at comparable proportions in the airway epithelium, from the larger lower airways (CCF score = 0.36) down to the distal lobular airways (CCF score = 0.81), while being largely absent in the lung parenchyma (CCF score = 0.97). In contrast, neuroendocrine cells are predominantly observed in the larger lower airways but are absent from more distal parts of the bronchial tree (Fig. 4c). In some cases, these proportions are highly dependent on the tissue sampling method and the dissociation protocol used (for example, for smooth muscle FAM83D^+^ cells; Extended Data Fig. 5). These observations shed light on the effects of biological and technical factors on the abundance of cell types in different parts of the lung and can help guide important choices in study design.

### HLCA-based analysis of lung data highlights new cell types

**Fig. 5:**
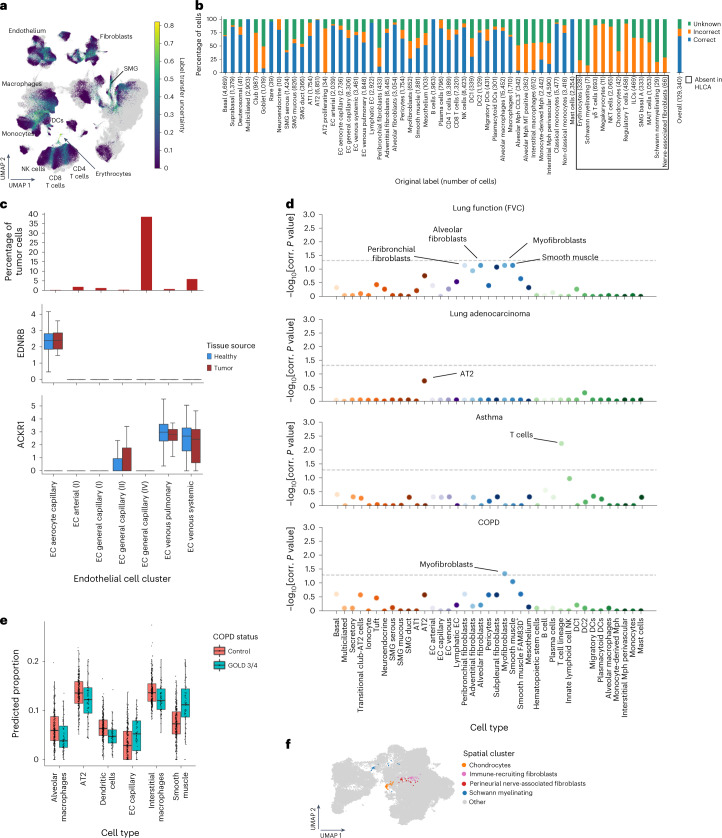
The HLCA core serves as a reference for label transfer and data contextualization. **a**, UMAP of the jointly embedded HLCA core (gray) and the projected healthy lung dataset (colored by label transfer uncertainty). HLCA cell types surrounding regions of high uncertainty are labeled. **b**, Percentage of cells from the newly mapped healthy lung dataset that are annotated either correctly or incorrectly by label transfer annotation or annotated as unknown, split by original cell type label (number of cells in parentheses). Cell type labels not present in the HLCA are boxed. **c**, Top, percentage of cells derived from tumor tissue, per endothelial cell cluster from the joint HLCA core and lung cancer data embedding. Only clusters with at least ten tumor cells are shown. Clusters are named based on the dominant HLCA core cell type annotation in the cluster. Middle, box plot showing the expression of *EDNRB* in endothelial cell clusters, split by tissue source. Bottom, as in the middle plot but for the expression of *ACKR1*. Numbers of cells per group were as follows: 6,574 (endothelial cell aerocyte capillary), 7,379 (endothelial cell arterial (I)), 10,906 (endothelial cell general capillary (I)), 3,440 (endothelial cell general capillary (II)), 2,859 (endothelial cell general capillary (III)), 6,318 (endothelial cell venous pulmonary) and 7,161 (endothelial cell venous systemic). **d**, Association of HLCA cell types with four different lung phenotypes based on previously performed GWASs. The horizontal dashed lines indicate a significance threshold of *α* = 0.05. *P* values were calculated using linkage disequilibrium score regression (Methods) and multiple testing corrected with the Benjamini–Hochberg procedure. **e**, Cell type proportions in lung bulk expression samples as estimated from HLCA-based cell type deconvolution, comparing controls (*n* = 281) versus donors with severe COPD (GOLD stage 3/4; *n* = 83). **f**, UMAP of fibroblast-dominated clusters from the jointly embedded HLCA core and mapped healthy lung dataset, colored by spatial cluster, with cells outside of the indicated clusters colored in gray. For all boxplots, the boxes show the median and interquartile range. Data points more than 1.5 times the interquartile range outside the low and high quartile are considered outliers. In **c**, these are not shown (see Supplementary Fig. 6 for full results), whereas in **e**, they are shown. Whiskers extend to the furthest nonoutlier point. corr., corrected; FVC, forced vital capacity; MAIT cells, mucosal-associated invariant T cells; NKT cells, natural killer T cells.

**Fig. 6:**
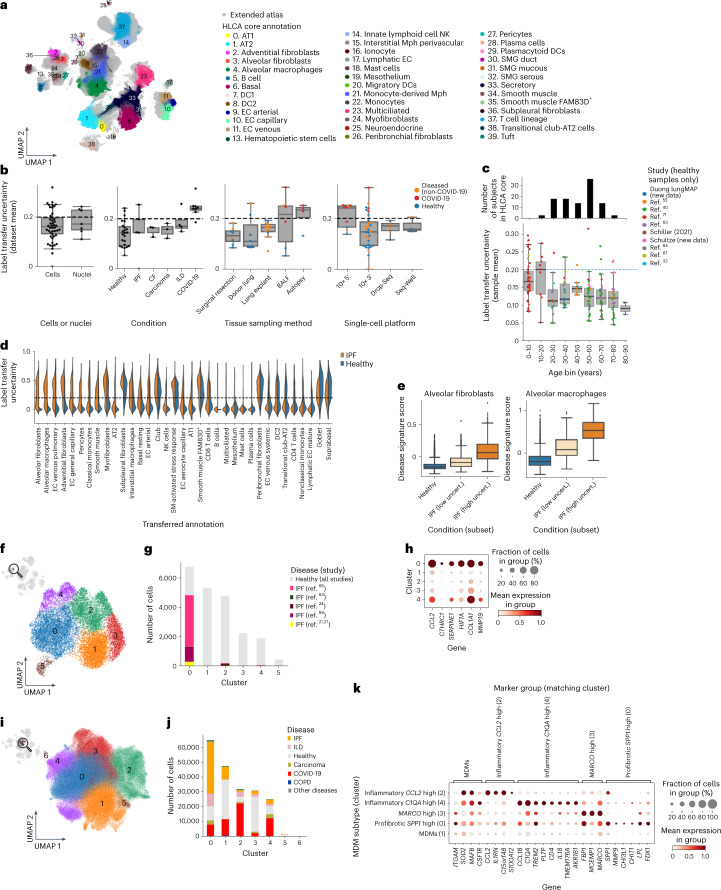
The extended HLCA enables the identification of disease-associated cell states. **a**, UMAP of the extended HLCA colored by coarse annotation (HLCA core) or in gray (cells mapped to the core). **b**, Uncertainty of label transfer from the HLCA core to newly mapped datasets, categorized by several experimental or biological features. Categories with fewer than two instances are not shown. The numbers of datasets per category were as follows: 30 cells, 7 nuclei, 23 healthy, 5 IPF, 3 CF, 3 carcinoma, 4 ILD, 8 surgical resection, 7 donor lung, 12 lung explant, 6 bronchoalveolar lavage fluid, 4 autopsy, 9 10x 5′, 31 10x 3′, 4 Drop-Seq and 3 Seq-Well. **c**, Bottom, mean label transfer uncertainty per mapped healthy lung sample in the HLCA extension, grouped into age bins and colored by study. The numbers of mapped samples per age bin were as follows: 43 for 0–10 years, 33 for 10–20 years, 31 for 20–30 years, 23 for 30–40 years, 19 for 40–50 years, 12 for 50–60 years, 9 for 60–70 years, 8 for 70–80 years and 2 for 80–90 years. Top, bar plot showing the number of donors per age group in the HLCA core. **d**, Violin plot of label transfer uncertainty per transferred cell type label for a single mapped IPF dataset^64^, split into cells from healthy donors (blue) and donors with IPF (orange). **e**, Uncertainty-based disease signature scores among alveolar fibroblasts and alveolar macrophages, split into cells from control donors (*n* = 10,453 and 1,812, respectively), and low-uncertainty cells (*n* = 1,419 and 200, respectively) and high-uncertainty cells (*n* = 1,172 and 162, respectively) from donors with IPF. **f**, UMAP embedding of alveolar fibroblasts (labeled with manual annotation (core) or label transfer (five IPF datasets)) colored by Leiden cluster. **g**, Composition of the clusters shown in **f** by study, with cells from control samples colored in gray. **h**, Expression of marker genes for IPF-enriched cluster 0 per alveolar fibroblast cluster. Cluster 5 was excluded as 96% of its cells were from a single donor. **i**, UMAP of all MDMs in the HLCA, colored by Leiden cluster. **j**, Composition of the MDM clusters from **i** by disease. **k**, Expression of cluster marker genes among all MDM clusters excluding donor-specific clusters 5 and 6. For **h** and **k**, mean counts were normalized such that the highest group mean was set to 1 for each gene. For **b**, **c** and **e**, the boxes show the median and interquartile range. Data points more than 1.5 times the interquartile range outside the low and high quartile are considered outliers. Whiskers extend to the furthest nonoutlier point. BALF, bronchoalveolar lavage fluid; CF, cystic fibrosis; Drop-Seq, droplet sequencing; ILD, interstitial lung disease; Mph, macrophages; SM, smooth muscle; uncert., uncertainty.

**Extended Data Fig. 6:**
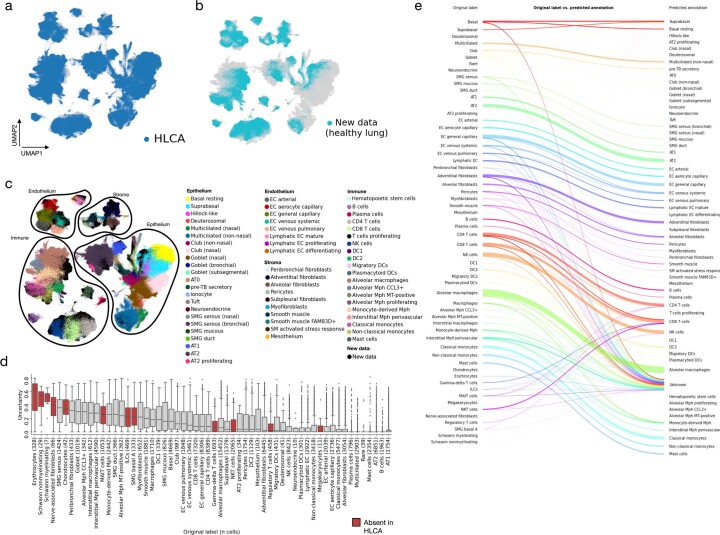
Mapping of unseen healthy lung scRNA-seq data to the HLCA core. **a**, UMAP of the jointly embedded HLCA core (dark blue, plotted on top) and the newly mapped healthy lung data (gray). **b**, Same as a, but now plotting cells from the HLCA in gray, and cells from the new data on top in light blue. **c**, Same as a, but now coloring cells from the HLCA core by their final annotation, and coloring cells from the new data in black. Cells from each of the compartments are outlined to ease visual identification of cell types by colors. **d**, Uncertainty of label transfer (ranging from 0 to 1) for cells from the mapped data, subdivided by original cell type label. Number of cells per label is shown between brackets. Cell labels are ordered by mean uncertainty. Boxes of cell labels not present in the HLCA core are colored red. Boxes show median and interquartile range of uncertainty. Cells with uncertainties more than 1.5 times the interquartile range away from the high and low quartile are considered outliers and plotted as points. Whiskers extend to the furthest non-outlier point. **e**, Sankey plot of original labels of cells from the mapped dataset versus predicted annotations based on label transfer. Cells with uncertainty >0.3 are labeled ‘unknown’. AT: alveolar type. DC: dendritic cells. EC: endothelial cells. ILCs: innate lymphoid cells. MAIT cells: mucosal-associated invariant T cells. MT: metallothionein. Mph: macrophages. NK: natural killer. NKT cells: natural killer T cells. SM: smooth muscle. SMG: submucosal gland. TB: terminal bronchiole.

The HLCA core contains an unprecedented diversity of donors, sampling protocols and cell identities, and can serve as a transcriptomic reference for lung research. New datasets can be mapped to this reference to substantially speed up data analysis by transferring consensus cell identity annotations to the new data. We tested this on a recently released multimodal lung dataset^40^ (Methods, Fig. 6a and Extended Data Fig. 6). Overall, the transferred labels were correct in the majority of cases, with 68% of the cells correctly labeled, 14% of labels incorrectly labeled and 18% set to unknown due to highly uncertain labeling (Fig. 5b and Methods). Uncertain labels were observed specifically in continuous transitions from one cell type to another and among cellular identities not present in the HLCA core, including rare cell identities (erythrocytes (*n* = 328), chondrocytes (*n* = 42), myelinating Schwann cells (*n* = 7), nonmyelinating Schwann cells (*n* = 29) and nerve-associated fibroblasts (*n* = 66); Fig. 5b and Extended Data Fig. 6d). Taken together, these results show that the HLCA core can be used for highly detailed annotation of new datasets, while allowing for the identification of unknown cell types in these datasets based on label transfer uncertainty.

### The HLCA provides crucial context for understanding disease

**Extended Data Fig. 7:**
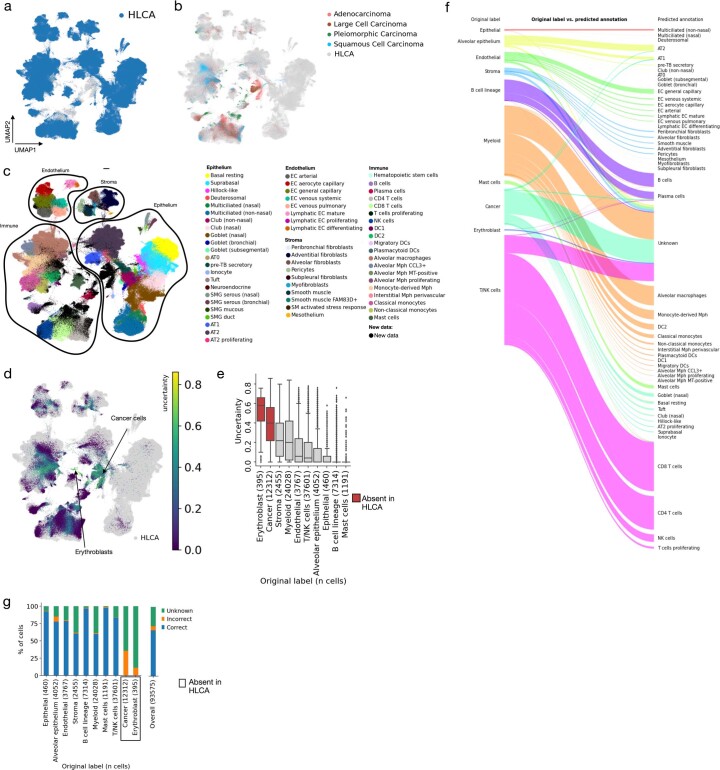
Mapping of unseen lung cancer data to the HLCA. **a**, UMAP of the jointly embedded HLCA (dark blue, plotted on top) and lung cancer data (gray). **b**, Same as a, but now plotting cells from the HLCA core in gray. Cells from the mapped data are plotted on top, and colored by the cancer type of the patient. **c**, Same as **a**, but now coloring cells from the HLCA core by their final final annotation, and coloring cells from the mapped cancer data in black. Cells from each of the compartments are outlined to ease visual identification of cell types by colors. **d**, Uncertainty of label transfer, shown for all cells from the mapped data. Regions dominated by high-uncertainty cells are labeled by the original cell type label. Cells from the HLCA core are colored in gray. **e**, Uncertainty of label transfer (ranging from 0 to 1) for the mapped cells, subdivided by original cell type label. Number of cells per label is shown between brackets. Boxes of cell type labels not present in the HLCA core are colored red. Cell types are ordered by mean uncertainty. Boxes show median and interquartile range of uncertainty. Cells with uncertainties more than 1.5 times the interquartile range away from the high and low quartile are considered outliers and plotted as points. Whiskers extend to the furthest non-outlier point. **f**, Sankey plot of original labels of the mapped data versus predicted annotations based on label transfer. Cells with uncertainty >0.3 are labeled ‘unknown’. **g**, Percentage of cells from newly mapped healthy lung dataset that are either annotated correctly or incorrectly by label transfer annotation (matched at the level of the original labels), or annotated as unknown, subdivided by original cell type label. The number of cells in the mapped dataset labeled with each label are shown between brackets after cell type names. Cell type labels not present in the HLCA are boxed. AT: alveolar type. DC: dendritic cells. EC: endothelial cells. MT: metallothionein. Mph: macrophages. NK: natural killer. SM: smooth muscle. SMG: submucosal gland. TB: terminal bronchiole.

Single-cell studies of disease rely on adequate, matching control samples to allow correct identification of disease-specific changes. To demonstrate the ability of the HLCA core to serve as a comprehensive healthy control and contextualize disease data, we mapped scRNA-seq data from lung cancer samples^41^ to the HLCA core (Methods and Extended Data Fig. 7a–c). Using HLCA label transfer, we correctly identified cell states missing from the HLCA core as unknown (cancer cells and erythroblasts). The remaining cells were annotated correctly in 77%, incorrectly in 1% and as unknown in 22% of cases (Extended Data Fig. 7d–g). A finding of the original study was the separation of endothelial cells into tumor-associated and normal cells^41^. Clustering of the projected dataset with the HLCA reference showed that cells expressing the suggested tumor-associated marker *ACKR1* were also abundant in healthy tissue from the HLCA core, specifically in venous endothelial cells (both pulmonary and systemic, Fig. 5c and Supplementary Fig. 6a–c). This suggests that *ACKR1* is a general marker of venous endothelial cells rather than a tumor-specific endothelial cell marker. Similarly, the reported normal endothelial cell marker *EDNRB* characterizes aerocyte capillary endothelial cells, both in tumor and in healthy tissue (Fig. 5c and Supplementary Fig. 6d). As endothelial cell numbers in the original study were low, correctly identifying and distinguishing these cell types without a larger healthy reference is challenging. Thus, by serving as a comprehensive healthy control, the HLCA prevents misinterpretation of limitations in sampling and experimental design as meaningful differences between healthy and diseased tissue.

In addition, the HLCA can provide context to the results of large-scale genetic studies of disease. Genome-wide association studies (GWASs) link disease with specific genomic variants that may confer an increased risk of disease. Previous studies have linked such variants to cell type-specific mechanistic hypotheses, which are often lacking in the initial association study. Yet, these studies fail to include all known lung cell types in their cell type reference^42,43^. To demonstrate the value of the HLCA core in contextualizing genetic data, we mapped association results from four GWASs of lung function or disease^44–47^ to the HLCA core cell types, by testing significant enrichment of both weakly and strongly disease-associated variants in regions of genes that characterize each cell type^48^ (Fig. 5d, Supplementary Fig. 7 and Methods). We show that genomic variants linked to lung function (forced vital capacity) are associated with smooth muscle (adjusted *P* value (*P*_adj_) = 0.07), alveolar fibroblasts (*P*_adj_ = 0.07), peribronchial fibroblasts (*P*_adj_ = 0.07) and myofibroblasts (*P*_adj_ = 0.07), suggesting that these fibroblast subtypes play a causative role in inherited differences in lung function. We further find a significant association of lung T cells with asthma-associated single-nucleotide polymorphisms (SNPs) (*P*_adj_ = 0.005). Lung adenocarcinoma-associated variants trend towards AT2 cells (*P*_adj_ = 0.18) and myofibroblasts are significantly associated with chronic obstructive pulmonary disease (COPD) GWAS SNPs (*P*_adj_ = 0.04). Thus, by linking genetic predispositions to lung cell types, the HLCA core serves as a valuable resource with which to improve our understanding of lung function and disease.

Finally, the HLCA can be used as a reference for cell type deconvolution of bulk RNA expression samples, which have been shown to reflect cell type proportions more accurately than scRNA-seq datasets^49^. Inferring cell type proportions from bulk RNA samples from nasal brushings and bronchial biopsies using the HLCA core (Supplementary Table 10, Supplementary Fig. 8a and Methods) revealed no significant cell type compositional changes associated with corticosteroid inhalation^50^ or asthma^51^, respectively (Supplementary Fig. 8b,c and Supplementary Table 11). In contrast, we find that the proportion of capillary endothelial cells in lung resection tissue from the Lung Tissue Database^52^ is higher in samples from patients with severe COPD (GOLD stage 3 or 4) than in those from non-COPD controls matched for age and smoking history (*P*_adj_ = 0.0004). Conversely, alveolar and interstitial macrophages, AT2 cells and dendritic cells decrease in proportion (Fig. 5e, Supplementary Fig. 8d and Supplementary Table 11; *P*_adj_ = 0.0007, 0.0003, 0.005 and 3.21 × 10^−6^, respectively). Finally, smooth muscle shows the largest shift in proportion, increasing significantly in patients with severe COPD (*P* = 1.85 × 10^−6^) in line with previous work^53^. As deconvolution of bulk samples using the HLCA can reveal disease-specific changes in cell type composition, we provide publicly available preprocessed cell type signature matrices based on the HLCA core (https://github.com/LungCellAtlas/HLCA).

### Extending the HLCA by projecting new data

As knowledge of cell types in the lung expands, and the sizes of newly generated datasets increase, annotations in the HLCA core will need to be further refined. The HLCA and its annotations can be updated by learning from new data projected onto the reference. We simulated such an HLCA update using the previously projected healthy lung dataset, specifically focusing on the cell identities that were distinguished based on their tissue location in matched spatial transcriptomic data (spatially annotated cell types)^40^. These cell identities were present at very low frequencies (median: 0.005% of all cells; Supplementary Fig. 9a). Both spatially annotated mesenchymal cell types with more than 40 cells (immune-recruiting fibroblasts and chondrocytes) and two rare cell types (myelinating Schwann cells and perineurial nerve-associated fibroblasts) were recovered in distinct clusters (spatially annotated clusters), and three of these (all except chondrocytes) also contained cells from the HLCA core, thereby enabling a refinement of existing HLCA core annotations using the spatial context from the projected dataset (Fig. 5f and Supplementary Fig. 9b,c). In this manner the HLCA core and its annotations can be refined by mapping new datasets to the atlas and incorporating annotations from these new datasets into the reference.

### Mapping data to the HLCA highlights disease-related states

**Extended Data Fig. 8:**
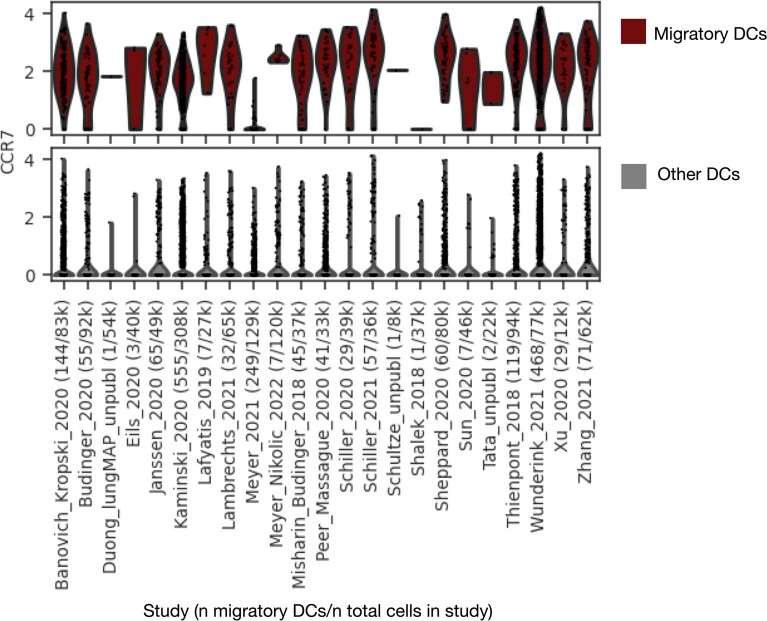
Expression of *CCR7* among cells annotated as migratory DCs by label transfer. Expression of *CCR7* is shown for all cells that were annotated as migratory DCs with low uncertainty (<0.2) (top) and all other cells annotated as DC (bottom) by label transfer from the HLCA core to the extended HLCA. Cells are grouped based on study of origin (some studies contain multiple datasets). X-tick labels show study, number of cells annotated as migratory DCs, and number of total cells (in thousands) per study. *CCR7* counts shown are counts that were normalized based on the total count among 2000 genes used for mapping to the HLCA core, and then log-transformed. DCs: dendritic cells.

To extend the atlas and include samples from lung disease, we mapped 1,797,714 cells from 380 healthy and diseased individuals from 37 datasets (four unpublished and 33 published^21,24,26,27,33,40,41,54–70^) to the HLCA core using scArches^71^, bringing the HLCA to a total of 2.4 million cells from 486 individuals (Fig. 6a and Supplementary Table 1). Label transfer from the HLCA core to the newly mapped datasets enabled detailed cell type annotation across datasets even for rare cells, including 2,048 migratory dendritic cells identified across 28 datasets with label transfer, whereas this cell type was originally labeled in only two of 12 labeled datasets (Extended Data Fig. 8).

Out of 37 new datasets, 27 were observed to map well to the HLCA, as evaluated by the mean label transfer uncertainty score (Fig. 6b, Supplementary Fig. 10a and Methods). The remaining ten datasets were often from coronavirus disease 2019 (COVID-19) studies or, unlike the HLCA core, contained pediatric samples (Fig. 6b,c and Supplementary Fig. 10b). In these datasets, higher uncertainty values may be attributable to true biological differences between the mapped data and the HLCA core adult, healthy lung samples. Overall, the successfully mapped datasets include disease samples, as well as single-nucleus and single-cell data from multiple chemistries (Fig. 6b), demonstrating the potential of the HLCA core as a universal reference.

**Extended Data Fig. 9:**
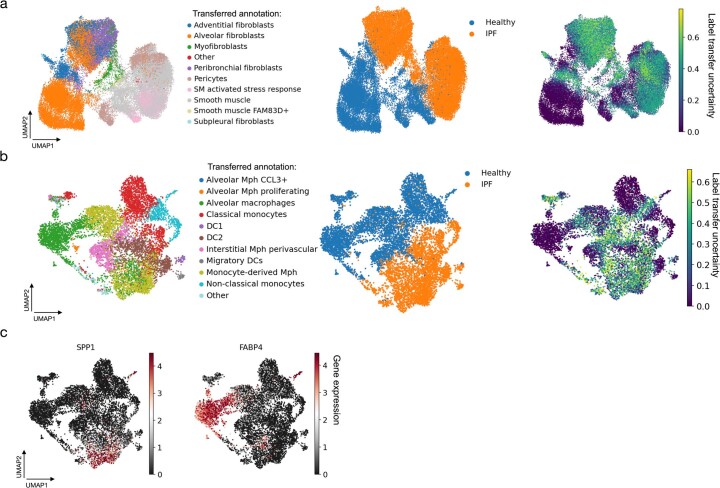
Transferred labels and matching uncertainty for a mapped IPF dataset. **a**, UMAPs of cells originally labeled as stroma, from a mapped IPF dataset^56^ including both healthy and IPF samples. Cells are labeled by annotation transferred from the HLCA core (left), by disease status (middle), and by label transfer uncertainty (right). Cells with labels transferred to fewer than 10 cells were excluded. **b**, same as **a**, but showing cells originally labeled as macrophages. **c**, As **b**, but now colored by expression of *SPP1* and *FABP4*. SM: smooth muscle. Mph: macrophages. DC: dendritic cells.

Pulmonary diseases are characterized by the emergence of unique disease-associated transcriptional phenotypes^4,21,22,24,72^. We observed higher levels of label transfer uncertainty in datasets from diseased lungs (Fig. 6b, condition), possibly flagging cell types changed in response to disease. Specifically, labels of alveolar fibroblasts and alveolar macrophages, which interact to form a dysregulated cellular circuit in idiopathic pulmonary fibrosis (IPFs)^21,22,24^, are transferred with higher uncertainty in IPF samples than in samples from healthy controls from the same dataset^64^ (Fig. 6d and Extended Data Fig. 9a,b). Furthermore, uncertainty scores separate cells—derived from donors with IPF—within these cell types into more and less affected subsets: the genes more highly expressed in the high-uncertainty subset are also lowly expressed in healthy samples (Fig. 6e). Genes downregulated in high-uncertainty IPF macrophages are associated with homeostatic functions of tissue-resident alveolar macrophages and lipid metabolism (*PPARG*, *FABP4* and others)^22,24,58^, while upregulated genes are associated with extracellular matrix remodeling and scar formation in the context of lung fibrosis (*SPP1*, *PLA2G7* and *CCL2*; Supplementary Tables 12 and 13 and Extended Data Fig. 9b,c)^22,24,58^. Thus, the HLCA core can be used to annotate new data, identify previously unreported populations, and—using label transfer uncertainty scores—help to detect disease-affected cell states and corresponding gene expression programs. This vastly speeds up analysis and interpretation of new data, automatically prioritizing the most relevant populations. Automated mapping of new data to the HLCA core can be done by any user via an interactive web portal (https://github.com/LungCellAtlas/HLCA) or using code tutorials as provided online.

### The HLCA reveals common aberrant cell states across diseases

**Extended Data Fig. 10:**
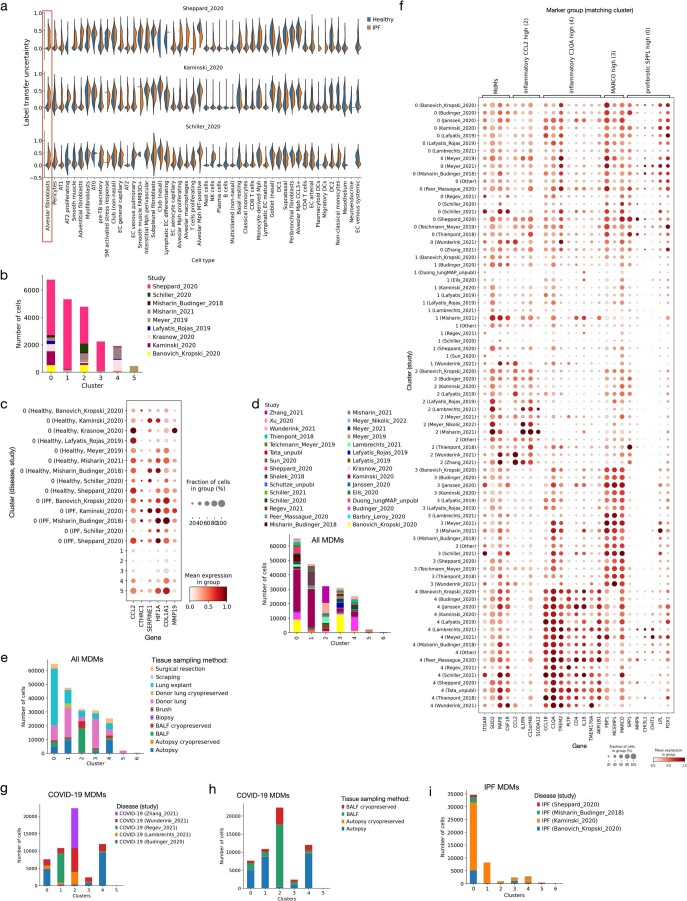
Disease-specific cellular states and states shared across diseases in the extended HLCA. **a**, Label transfer uncertainty shown per cell type, comparing cells from control samples (‘healthy’, blue) to cells from IPF samples (orange). Results are shown per dataset, only showing datasets that include both control and IPF mapped samples. Alveolar fibroblasts, the cell type chosen for downstream analysis, are boxed in red. AT: alveolar type. DC: dendritic cell. TB: terminal bronchiole. EC: endothelial cell. Mph: macrophage. MT: metallothionein. NK: natural killer. SM: smooth muscle. **b**, Composition of alveolar fibroblast clusters by study. **c**, Expression of several genes highly expressed in IPF-enriched alveolar fibroblast cluster 0, shown per cluster. Cluster 0 is split into control (‘Healthy’) and IPF, further subdivided by study. **d**, Composition of monocyte-derived macrophage (MDM) clusters by study. **e**, As **d**, but by tissue sampling method. **f**, Expression of MDM cluster marker genes shown per cluster, with clusters split into studies. Studies with fewer than 200 were grouped into ‘Other’ for each cluster. **g**, Composition of MDM clusters by study, subsetted to only cells from donors with COVID-19. **h**, As **g**, but by tissue sampling method. **i**, As **g**, but subsetted to cells from donors with IPF. For **c** and **f**, mean expressions were normalized such that the highest mean expression was set to 1 for each gene. BALF: bronchoalveolar lavage fluid. IPF: idiopathic pulmonary fibrosis.

Similar to healthy cellular states, the HLCA can provide insight into disease-specific states that are consistent across demographics and experimental protocols. To demonstrate this, we determined which cell types are consistently affected by IPF across datasets, extending the previous IPF analysis to five independent datasets. We found that cells labeled as alveolar fibroblasts consistently show high uncertainty levels in IPF samples compared with controls across all mapped IPF datasets that include controls^58,62,64^ (Extended Data Fig. 10a). Clustering of alveolar fibroblasts from the HLCA core and all IPF datasets^21,24,58,62,64^ shows that cells from patients with IPF predominantly cluster together in a single cluster (Fig. 6f,g and Extended Data Fig. 10b) characterized by high expression of genes previously associated with IPF^64,73,74^ (*CCL2*, *COL1A1*, *CTHRC1* and *MMP19*), as well as further fibrosis-associated markers (*SERPINE1*, an inhibitor of extracellular matrix breakdown^75^, and *HIF1A*, a chronic hypoxia response gene^76^; Fig. 6h and Supplementary Table 14). These marker genes are consistently expressed across datasets (Extended Data Fig. 10c), confirming that the identification of this IPF-specific alveolar fibroblast state is reproducible.

The HLCA contains data across more than ten lung diseases, providing the unique opportunity to discover cellular states shared across diseases. Discovering such common diseased cellular states could improve our understanding of lung diseases and accelerate the identification of effective treatments. For example, profibrotic *SPP1*^+^ monocyte-derived macrophages (MDMs) have previously been reported in COVID-19, IPF and cancer^26,77,78^. To test whether similar cross-disease MDM states could be discovered in the HLCA, we performed clustering of all MDMs from the HLCA (Fig. 6i). We identified four main MDM subtypes (Methods and Supplementary Table 15), each showing distinct gene expression and disease enrichment patterns, and representing different stages of monocyte-to-MDM differentiation and adaptation to the disease microenvironment. First, an early and inflammatory MDM state was observed that was high in the expression of *CCL2*, a gene involved in the recruitment of immune cells. This cluster predominantly contained cells from bronchoalveolar lavage fluid samples collected early during the course of COVID-19 pneumonia (cluster 2; *IL1RN*^high^ and *S100A12*^high^; Fig. 6i–k and Extended Data Fig. 10d–h). We further observed an MDM subset expressing inflammation and phagocytosis-associated genes (cluster 4; *CCL18*, *IL18*, *C1QA* and *TREM2*) and enriched for samples from patients with COVID-19 pneumonia, as well as samples from patients with lung carcinoma (Fig. 6i–k and Extended Data Fig. 10d–h). A third MDM subset represented a more differentiated MDM phenotype, as indicated by the expression of *MARCO* and *MCEMP1*, dominated by cells from nondiseased samples (cluster 3; Fig. 6i–k and Extended Data Fig. 10d,f). The final MDM subset was dominated by IPF samples. Interestingly, this cluster was also enriched for cells from patients who died late in the course of COVID-19 and developed post-COVID-19 lung fibrosis, as well as cells from patients with lung carcinoma (cluster 0; Fig. 6i–k and Extended Data Fig. 10g–i). This multidisease cluster is marked by high expression of *SPP1*, *LPL* and *CHIT1*—markers that have been shown to play a causal role in the development of lung fibrosis^22,79–81^ (Fig. 6k), one of which (*CHIT1*) is currently being investigated as a therapeutic target for IPF^82^. The expression of these markers is consistent across diseases and studies (Extended Data Fig. 10f), suggesting that also in cancer and late-stage COVID-19 samples a subset of MDMs adopt a fibrosis-associated phenotype. Together, this analysis shows that the HLCA enables a better understanding of cellular states shared between diseases and thereby has the potential to accelerate the discovery of effective disease treatments.

## Discussion

In this study, we built the HLCA: an integrated reference atlas of the human respiratory system. While previous studies have described the cellular heterogeneity within the human lung^4–6,24,58^, study-specific biases due to experimental design and a limited number of sampled individuals constrain their capacity to capture population variation and serve as a universal reference. The HLCA integrates data from 49 datasets to produce such a reference of 2.4 million cells, covering all major lung scRNA-seq studies published to date. The core of this atlas consists of a fully integrated healthy reference of 14 datasets with 61 cell identities, including rare and novel cell types, representing a data-derived consensus annotation of the cellular landscape of the human lung. We leveraged the unprecedented complexity of the HLCA to recover cell type-specific gene modules associated with covariates such as lung anatomical location, age, sex, BMI and smoking status. By projecting data onto the HLCA, we showed that the HLCA enables a fast and detailed annotation of new datasets, as well as the identification of unique, disease-associated cell states and cell states common to multiple diseases. The HLCA is publicly available as a resource for the community, together with an online platform for automated mapping of new data. Taken together, the HLCA is a universal reference for single-cell lung research that promises to accelerate future studies into pulmonary health and disease.

The ultimate goal of a human lung cell atlas reference is to provide a comprehensive overview of all cells in the healthy human lung, as well as their variation from individual to individual. Despite its overall diversity, the HLCA is limited by the biological, demographic and experimental diversity in the foundational single-cell studies. For example, 65% of the HLCA core data are from individuals of European harmonized ethnicity, highlighting the urgent need for diversification of the population sampled in lung studies. Moreover, ethnicity metadata were based on self-reports and harmonized across datasets, which is an imperfect approach to representing the diversity of the atlas. SNP-based inference of genetic ancestry constitutes a more objective and therefore preferable approach to the grouping of individuals based on genetic background and would aid in better assessing the genetic diversity captured in the atlas. Overall, more diverse samples will enrich the atlas, diversify captured cell identities and improve the quality of the HLCA as a reference for new datasets. Such a reference will also enable comparison with model systems such as mice, cell lines or organoids, although further method development may be required to map across diverse in vitro and clinical datasets.

The constituent datasets of the HLCA vary widely in experimental design, such as the sample handling protocol or single-cell platform used, causing dataset-specific batch effects. The quality of the HLCA hinges on the choice of data integration method, with methods such as Seurat’s RPCA^30^ and Harmony^29^ failing to correctly group rare cell identities into separate clusters. Nevertheless, also in the HLCA, certain subsets of T cells (regulatory T cells and γδ T cells) could not be identified as separate clusters, showing the limitations of the current HLCA in capturing cellular heterogeneity for a subset of immune cell types. Mapping additional datasets with high-resolution annotations (for example, derived from multimodal data) could provide the power to detect these cell identities in the atlas. Indeed, the HLCA must be viewed as a live resource that requires continuous updates. While we showed that mapping new, spatially annotated data to the HLCA core can refine HLCA annotations, this new knowledge must be consolidated by regular updates of the HLCA with new datasets (including epigenomic, spatial and imaging data) and refinements of HLCA annotations based on additional expert opinions. Thereby, the HLCA can serve as a community- and data-driven platform for open discussion on lung cell identities as the respiratory community progresses in charting the cellular landscape of the lung. In this process, we envision that the HLCA will be completed in two phases: first on the level of cellular variation (when no new consensus cell types can be found) and then in the description of individual variation (when population diversity is fully represented).

Taken together, the HLCA provides a central single-cell reference of unprecedented size. It offers a model framework for building integrated, consensus-based, population-scale atlases for other organs within the Human Cell Atlas. The HLCA is publicly available, and combined with an open-access platform to map new datasets to the atlas, this resource paves the way toward a better and more complete understanding of both health and disease in the human lung.

## Methods

### Ethics approval and consent

Ethics approval information per study was as follows. For the pooled data from refs. ^21,27^, approval was given by the Vanderbilt Institutional Review Board (IRB) (numbers 060165 and 171657) and Western IRB (number 20181836). All samples were collected from declined organ donors who were also consented for research. For ref. ^6^, the study was approved by the Comité de Protection des Personnes Sud Est IV (approval number 17/081). Informed written consent was obtained from all participants involved. For Jain_Misharin_2021 (A.V.M., M.J. and N.S.M., newly generated dataset), the protocol was approved by the Northwestern University IRB (STU00214826). Written informed consent was obtained from all study participants. For ref. ^5^, patient tissues were obtained under a protocol approved by Stanford University’s Human Subjects Research Compliance Office (IRB 15166). Informed consent was obtained from each patient before surgery. For ref. ^22^, healthy control lungs were obtained under a protocol approved by the University of Pittsburgh Committee for Oversight of Research and Clinical Training Involving Decedents (CORID protocol 718) and following rejection as candidate donors for transplant (IRB STUDY 19100326). For ref. ^23^, tissue samples were obtained from the Cambridge Biorepository for Translational Medicine (CBTM) with approval from the National Research Ethics Services (NRES) Committee of East of England—Cambridge South (15/EE/0152). Tissue samples were obtained with informed consent from the donor families. For ref. ^26^, the protocol was approved by the Northwestern University IRB (STU00212120). Written informed consent was obtained from all individuals in the study. For the pooled data from ref. ^4^ and associated unpublished data, the protocol was approved by the IRB (Algemeen Beoordelings- en Registratieformulier number NL69765.042.19). Patients gave informed consent. For ref. ^25^, the National Jewish Health IRB approved the research under IRB protocols HS-3209 and HS-2240. Informed consent was obtained from authorized family members of all donors. For ref. ^4^, approval was given by the NRES Committee of East of England—Cambridge South (Research Ethics Committee (REC) reference: 15/EE/0152). Informed consent for use of the tissue was obtained from the donors’ families. For Barbry_unpubl (P.B., L.-E.Z., M.J.A., A.C., C.B. et al., newly generated dataset), the protocol was approved by the Centre Hospitalier Universitaire de Nice. Nasal and tracheobronchial samples were collected from patients with IPF after obtaining their informed consent. For ref. ^26^, approved was given by the IRB of Northwestern University (STU00212120, STU00213177, STU00212511 and STU00212579). For inclusion in this study, patients or their designated medical power of attorney provided informed consent. For Duong_lungMAP_unpubl (T.E.D., K.Z., X.S., J.S.H. and G.P., newly generated dataset), all postmortem human donor lung samples were obtained from the Biorepository for Investigation of Neonatal Diseases of the Lung (BRINDL), supported by the National Heart, Lung, and Blood Institute (NHLBI) LungMAP Human Tissue Core housed at the University of Rochester. Consent can be found on the repository’s website (brindl.urmc.rochester.edu/). For ref. ^54^, the study was conducted in accordance with the Declaration of Helsinki and Department of Health and Human Services Belmont Report. The use of biomaterial and data for this study was approved by the local ethics committee of the Medical Faculty Heidelberg (S-270/2001 and S-538/2012). All individuals gave informed consent for inclusion before they participated in the study. For ref. ^55^, human lung tissues were procured under each institution’s approved IRB protocol (numbers 00035396 (Cedars-Sinai Medical Center), 03-1396 (University of North Carolina at Chapel Hill), 1172286 (Cystic Fibrosis Foundation and WIRB-Copernicus Group Western IRB) and 16-000742 (University of California, Los Angeles)). Informed consent was obtained from lung donors or their authorized representatives. For ref. ^57^, the study was approved and monitored by the National Jewish Health IRB (FWA00000778). Written informed consent was obtained from all participants. For ref. ^58^, the study protocol was approved by the Partners Healthcare IRB (protocol 2011P002419). For ref. ^60^, lung tissue was obtained under a protocol approved by the University of Pittsburgh IRB (IRB STUDY 19100326) during transplantation surgery. For ref. ^59^, the study was conducted according to the principles expressed in the Declaration of Helsinki. Ethical approval was obtained from Ethics Committee Research UZ/KU Leuven (S63881). All participants provided written informed consent for sample collection and subsequent analyses. For ref. ^40^, approval was given by the NRES Committee of East of England—Cambridge South (15/EE/0152). The CBTM operates in accordance with UK Human Tissue Authority guidelines. Samples were obtained from deceased transplant organ donors by the CBTM with informed consent from the donor families. For ref. ^70^, ethical approval was given through the Living Airway Biobank, administered through the University College London Great Ormond Street Institute of Child Health (REC reference: 19/NW/0171; Integrated Research Application System (IRAS) project ID: 261511; North West Liverpool East REC), REC reference 18/SC/0514 (IRAS project ID: 245471; South Central Hampshire B REC; administered through the University College London Hospitals NHS Foundation Trust), REC reference 18/EE/0150 (IRAS project ID: 236570; East of England—Cambridge Central REC; administered through Great Ormond Street Hospital NHS Foundation Trust) and REC reference 08/H0308/267 (administered through the Cambridge University Hospitals NHS Foundation Trust), as well as by the local R&D departments at all hospitals. All of the study participants or their surrogates provided informed consent. For ref. ^61^, all protocols were reviewed and approved by the IRB at the Memorial Sloan Kettering Cancer Center (IRB protocol 14-091). Noninvolved lung, tumor tissues and metastatic lesions were obtained from patients with lung adenocarcinoma undergoing resection surgery at the Memorial Sloan Kettering Cancer Center after obtaining informed consent. For ref. ^69^, samples underwent IRB review and approval at the institutions where they were originally collected. Specifically, the Dana-Farber Cancer Institute approved protocol 13-416, the partners Massachusetts General Hospital and Brigham and Women’s Hospital approved protocols 2020P000804, 2020P000849 and 2015P002215, the Beth Israel Deaconess Medical Center approved protocols 2020P000406 and 2020P000418 and New York Presbyterian Hospital/Columbia University Irving Medical Center approved protocols IRB-AAAT0785, IRB-AAAB2667 and IRB-AAAS7370. Secondary analysis of samples at the Broad Institute was covered under Massachusetts Institute of Technology IRB protocols 1603505962 and 1612793224, or the Not Human Subjects Research protocol ORSP-3635. Donor identities were encoded at the hospitals before shipping to or sharing with the Broad Institute for sample processing or data analysis, respectively. For ref. ^62^, the study was approved by the local ethics committee of the Ludwig Maximilian University of Munich (EK 333-10 and 382-10). Written informed consent was obtained from all patients. For Schiller_2021 (H.B.S., J.G.-S., C.H.M., B.H.K., M.A. et al., newly generated dataset), the study was approved by the local ethics committee of the Ludwig Maximilian University of Munich (EK 333-10 and 382-10). Written informed consent was obtained from all patients. For Schultze_unpubl (J.L.S., C.S.F., T.S.K. and E.C., newly generated dataset), human lung tissue was available for research purposes following ethical approval from Hannover Medical School (ethical vote of the German Centre for Lung Research (DZL) number 7414, 2017). All patients in this study provided written informed consent for sample collection and data analysis. For ref. ^63^, samples were obtained under the Cells and Mediators IRB protocol (2003P002088). All individuals provided written informed consent. For ref. ^64^, the studies described were conducted according to the principles of the Declaration of Helsinki. The study was approved by the University of California, San Francisco IRB. Written informed consent was obtained from all individuals. For ref. ^65^, peripheral blood was obtained from healthy consenting adult volunteers by venipuncture through a protocol approved by the Columbia University IRB. All relevant ethical regulations for work with human participants were complied with. For ref. ^66^, donor lung samples were provided through the federal United Network for Organ Sharing via the National Disease Research Interchange and International Institute for the Advancement of Medicine and entered into the NHLBI LungMAP BRINDL at the University of Rochester Medical Center, overseen by the IRB as RSRB00047606. For for the pooled data from ref. ^33^ and associated unpublished data, human lung tissue collection was approved by the Duke University IRB (Pro00082379) and University of North Carolina Biomedical IRB (03-1396) under exempt protocols. Consent was obtained to use human tissues for research purposes. For ref. ^41^, the study was approved by the local ethics committee at University Hospitals Leuven (B322201422081) and all of the relevant ethical regulations were complied with. Only patients with untreated, primary, nonmetastatic lung tumors who underwent lung lobe resection with curative intent and who provided informed consent were included in this study. For ref. ^67^, all of the research involving human participants was approved by the Northwestern University IRB. Samples from patients with COVID-19, viral pneumonia and other pneumonia, as well as controls without pneumonia, were collected from participants enrolled in the Successful Clinical Response in Pneumonia Therapy study STU00204868. All study participants or their surrogates provided informed consent. For ref. ^56^, the IRB of the University of Cincinnati College of Medicine approved all human-relevant studies. For ref. ^68^, the study was conducted according to the principles expressed in the Declaration of Helsinki. Ethical approval was obtained from the REC of Shenzhen Third People’s Hospital (2020-112). All participants provided written informed consent for sample collection and subsequent analyses. Further study details can be found in Supplementary Table 1.

### Single-cell sequencing and preprocessing of data

Several previously unpublished datasets were used for the HLCA and generated as follows.

#### Barbry_unpubl

Participants recruited by the Pneumology Unit of Nice University Hospital were sampled between 1 and 15 December 2020. The full procedure, including patient inclusion criteria, is detailed at https://www.clinicaltrials.gov/ct2/show/NCT04529993. Nasal and tracheobronchial samples were collected from patients with IPF after obtaining their informed consent, following a protocol approved by the Centre Hospitalier Universitaire de Nice. The data were derived from the clinical trial registered at ClinicalTrials.gov under reference NCT04529993. This study was described as an interventional study instead of an observational study because the participants were volunteers and all assigned to a specific bronchoscopy not related to routine medical care. Participants were prospectively assigned to a procedure (bronchoscopy) according to a specific protocol to assess our ability to sample the airway. No other procedures were included in this study. Metadata of the donors’ sex was based on self-report. The libraries were prepared as described in Deprez et al.^6^ and yielded an average of 61,000 ± 11,000 cells per sample, with a viability above 95%. The single-cell suspension was used to generate single-cell libraries following the v3.1 protocol for 3′ chemistry from 10x Genomics (CG000204). Sequencing was performed on a NextSeq 500/550 sequencer (Illumina). Raw sequencing data were processed using the Cell Ranger 6.0.0 pipeline, with the reference genome GRCh38 and annotation using Ensembl98. For each sample, cells with fewer than 200 transcripts or more than 40,000 transcripts were filtered out, as well as genes expressed in fewer than three cells. Normalization and log transformation were done using the standard Scanpy^83^ pipeline. Principal component analysis (PCA) was performed on 1,000 highly variable genes (HVGs) to compute 50 principal components, and the Louvain algorithm was used for clustering. These clusters were then annotated by hand for each sample. Raw counts and the thus obtained cell annotations were used as input for the HLCA.

#### Schiller_2021

Tumor-free, uninvolved lung samples (peritumor tissues) were obtained during tumor resections at the lung specialist clinic Asklepios Fachkliniken München-Gauting and accessed through the bioArchive of the Comprehensive Pneumology Center in Munich. The study was approved by the local ethics committee of the Ludwig Maximilian University of Munich (EK 333-10 and 382-10), and written informed consent was obtained from all patients. All fresh tissues from patients in a given time frame without any specific selection criteria were included, and only patients with obvious chronic lung disease as comorbidity based on their lung function parameters before tumor resection were excluded. Metadata of the donors’ sex were based on self-report.

Single-cell suspensions for scRNA-seq were generated as previously described^62^. In brief, lung tissue samples were cut into smaller pieces, washed with phosphate-buffered saline (PBS) and enzymatically digested using an enzyme mix composed of dispase, collagenase, elastase and DNAse for 45 min at 37 °C while shaking. After inactivating the enzymatic activity with 10% fetal calf serum (FCS)/PBS, dissociated cells were passed through a 70 µm cell strainer, pelleted by centrifugation (300*g*; 5 min) and subjected to red blood cell lysis. After stopping the lysis with 10% FCS/PBS, the cell suspension was passed through a 30 µm strainer and pelleted. Cells were resuspended in 10% FCS/PBS, assessed for viability and counted using a Neubauer hematocytometer. The cell concentration was adjusted to 1,000 cells per µl and ~16,000 cells were loaded on a 10x Genomics Chip G with Chromium Single Cell 3′ v3.1 gel beads and reagents (3′ GEX v3.1; 10x Genomics). Libraries were prepared according to the manufacturer’s protocol (CG000204_RevD; 10× Genomics). After a quality check, scRNA-seq libraries were pooled and sequenced on a NovaSeq 6000 instrument.

The generation of count matrices was performed using the Cell Ranger computational pipeline (v3.1.0; STAR v2.5.3a). The reads were aligned to the GRCh38 human reference genome (GRCh38; Ensembl99). Downstream analysis was performed using the Scanpy^83^ package (version 1.8.0). We assessed the quality of our libraries and excluded barcodes with fewer than 300 genes detected, while retaining those with a number of transcripts between 500 and 30,000. Furthermore, cells with a high proportion (>15%) of transcript counts derived from mitochondrial-encoded genes were removed. Genes were considered if they were expressed in at least five cells. Raw counts of cells that passed filtering were used as input for the HLCA.

#### Duong_lungMAP_unpubl

All postmortem human donor lung samples were obtained from BRINDL, supported by the NHLBI LungMAP Human Tissue Core housed at the University of Rochester. Consent, tissue acquisition and storage protocols can be found on the repository’s website (brindl.urmc.rochester.edu/). Data were collected as part of the Human Biomolecular Atlas Program (HuBMAP). Metadata of the donor’s sex were based on self-report. For isolation of single nuclei, ten cryosections (40 µm thickness) from O.C.T.-embedded tissue blocks stored at −80 °C were shipped on dry ice and processed according to a published protocol^84^. Single-nucleus RNA-seq was completed using 10x Chromium Single Cell 3’ Reagent Kits v3, according to a published protocol^84,85^. Raw sequencing data were processed using the 10x Cell Ranger v3 pipeline and the GRCh38 reference genome. For downstream analysis, mitochondrial transcripts and doublets identified by DoubletDetection^86^ version 2.4.0 were removed. Samples were then combined and cell barcodes were filtered based on the genes detected (>200 and <7,500) and the gene unique molecular identifier (UMI) ratio (gene.vs.molecule.cell.filter function) using Pagoda2 (github.com/hms-dbmi/pagoda2). Also using Pagoda2 for clustering, counts were normalized to total counts per nucleus. For batch correction, gene expression was scaled to dataset average expression. After variance normalization, all significantly variant genes (*n* = 4,519) were used for PCA. Clustering was done at different *k* values (50, 100 or 200) using the top 50 principal components and the infomap community detection algorithm. Then, principal component and cluster annotations were imported into Seurat^30^ version 4.0.0. Differentially expressed genes for all clusters were generated for each *k* resolution using Seurat FindAllMarkers (only.pos = TRUE, max.cells.per.ident = 1000, logfc.threshold = 0.25, min.pct = 0.25). Clusters were manually annotated based on distinct differentially expressed marker genes. Raw counts and the thus obtained cell annotations were used as input for the HLCA.

#### Pooled data from ref. ^4^ and unpublished data

These data were a combination of published^4^ and unpublished data. In both cases, healthy volunteers were recruited for bronchoscopy at the University Medical Center in Groningen after giving informed consent and according to the protocol approved by the IRB (ABR number NL69765.042.19). Inclusion criteria and tissue processing were performed as previously described^4^. In short, all donors were 20–65 years old and had a history of smoking <10 pack-years. Metadata of the donors’ sex were based on self-report. To exclude respiratory disease, the following criteria were used: absent history of asthma or COPD; no use of asthma- or COPD-related medication; a negative provocation test (concentration of methacholine that provokes a 20% decrease in the forced expiratory volume in 1 s (FEV_1_) > 8 mg ml^−1^); no airflow obstruction (FEV_1_/forced vital capacity ≥ 70%); and an absence of lung function impairment (that is, FEV_1_ ≥ 80% predicted). All donors underwent a bronchoscopy under sedation using a standardized protocol^87^. Nasal brushes were obtained from the lateral inferior turbinate in a subset of the volunteers immediately before bronchoscopy using a Cyto-Pak CytoSoft nasal brush (Medical Packaging Corporation). Six macroscopically adequate endobronchial biopsies were collected for this study, located between the third and sixth generation of the right lower and middle lobe. Bronchial brushes were obtained from a different airway at similar anatomical locations using a Cellebrity bronchial brush (Boston Scientific). Extracted biopsies and bronchial and nasal brushes were processed directly, with a maximum of 1 h delay. Bronchial biopsies were chopped biopsies using a single-edge razor blade. A single-cell solution was obtained by tissue digestion using 1 mg ml^−1^ collagenase D and 0.1 mg ml^−1^ DNase I (Roche) in Hanks’ Balanced Salt Solution (Lonza) at 37 °C for 1 h with gentle agitation for both nasal brushes and bronchial biopsies. Single-cell suspensions were filtered and forced using a 70 µm nylon cell strainer (Falcon), followed by centrifugation at 550*g* and 4 °C for 5 min and one wash with PBS containing 1% bovine serum albumin (BSA; Sigma–Aldrich). The single-cell suspensions used for 10x Genomics scRNA-seq analysis were cleared of red blood cells using a red blood cell lysis buffer (eBioscience) followed by live cell counting and loading of 10,000 cells per lane. We used 10x Genomics Chromium Single Cell 3′ Reagent Kits v2 and v3 according to the manufacturers’ instructions. Raw sequencing data were processed using the Cell Ranger 3.1.0-based HLCA pipeline, with the reference genome GRCh38 and annotation using Ensembl98. Ambient RNA correction was performed with FastCAR^88^, using an empty library cutoff of 100 UMI and a maximum allowed contamination chance of 0.05, ignoring the mitochondrial RNA. Data were merged and processed using Seurat^30^, filtering to libraries with >500 UMIs and >200 genes and to the libraries containing the lowest 95% of mitochondrial RNA per sample and <25% mitochondrial RNA, normalized using sctransform^89^ while regressing out variation correlating with the percentage of mitochondrial RNA per cell. In general, 15 principal components were used for the clustering, at a resolution of 0.5 to facilitate manual annotation of the dataset. Clusters in the final object that were driven by single donors were removed. Raw counts and cell annotations were used as input for the HLCA.

#### Jain_Misharin_2021

Nasal epithelial samples were collected from healthy volunteers who provided informed consent at Northwestern Medicine in Chicago. The protocol was approved by the Northwestern University IRB (STU00214826). Healthy volunteers were recruited to match a cohort of patients with cystic fibrosis for the ongoing study at Northwestern University (with M.J. as the principal investigator). In both studies, A.V.M. did not influence participant recruitment and did not introduce biases in sample selection. Metadata of the donors’ sex were based on self-report. Briefly, donors were seated and asked to extend their neck. A nasal curette (Rhino-Pro; VWR) was inserted into either nare and gently slid in the direction of posterior to anterior ~1 cm along the lateral inferior turbinate. Five curettes were obtained per participant. The curette tip was then cut and placed in 2 ml hypothermosol and stored at 4 C until processing. A single-cell suspension was generated using the cold-active dispase protocol reported by Deprez et al.^6^ and Zaragosi and Barbry^90^ with slight modification. Specifically, ethylenediaminetetraacetic acid (EDTA) was omitted and cells were dispersed by pipetting 20 times every 5 min using a 1 ml tip instead of tritration using a 21/23 G needle. The final concentration of protease from *Bacillus licheniformis* was 10 mg ml^−1^. The total digestion time was 30 min. Following the wash in 4 ml 0.5% BSA in PBS and centrifugation at 400*g* for 10 min, cells were resuspended in 0.5% BSA in PBS and counted using a Nexcelom K2 Cellometer with acridine orange/propidium iodide reagent. This protocol typically yields ~300–500,000 cells with a viability of >95%. The resulting single-cell suspension was then used to generate single-cell libraries following the protocol for 5′ V1 (CG000086 Rev M; 10x Genomics) or V2 chemistry (CG000331 Rev A; 10x Genomics). Excess cells from two of the samples were pooled together to generate one additional single-cell library. After a quality check, the libraries were pooled and sequenced on a NovaSeq 6000 instrument. Raw sequencing data were processed using the Cell Ranger 3.1.0 pipeline, with the reference genome GRCh38 and annotation using Ensembl98. To assign sample information to cells in the single-cell library prepared from two samples, we ran souporcell^91^ version 2.0 for that library and two libraries that were prepared from these samples separately. We used common genetic variants prepared by the souporcell authors to separate cells into two groups by genotype for each library, and Pearson correlation between the identified genotypes across libraries to establish correspondence between genotype and sample. Cell annotations were assigned to cell clusters based on expert interpretation of marker genes for each cluster. Cell clusters were derived with the Seurat^30^ version 3.2 workflow in which samples were normalized with sctransform^89^, 3,000 HVGs were selected and integrated and clusters were derived from 30 principal components using the Louvain algorithm with default parameters. Clusters with a low number of UMIs and high expression of ribosomal or mitochondrial genes were excluded as low quality. Raw counts and the thus obtained cell annotations were used as input for the HLCA.

#### Schultze_unpubl

Human lung tissue wabus available for research purposes following ethical approval from Hannover Medical School (Nr. 7414, 2017). All patients in this study provided written informed consent for sample collection and data analyses. At Hannover Medical School, patients with lung cancer were recruited in the course of their operation (that is, surgical tumor resection was performed according to the ethical vote of the German Centre for Lung Research, ethical vote 7414 and data safety guidelines). There was no bias in patient recruitment since the samples were collected as fresh native tissue following surgical tumor resection and according to the availability of surplus adjacent nonmalignant lung tissue, which was resected in parallel to the tumor tissue. Metadata of the donors’ sex were based on self-report or reported by medical professionals during consenting. Fresh adjacent normal tumor-free lung tissues from patients with non-small cell lung cancer tumors were obtained by the Lung Research group (D. Jonigk, Pathology, Hannover Medical School) and processed for single-cell isolation immediately. Lung tissue was chopped with a scalpel and scissors and digested using BD Tumor Dissociation Reagent (BD Biosciences) for 30 min in a 37 °C water bath. The digestion was stopped with 1% FCS and 2 mM EDTA in PBS without Ca^2+^/Mg^2+^ and cells were filtered over a 70 µm cell strainer (BD Falcon). Erythrocytes were removed using a human MACSxpress Erythrocyte Depletion Kit (Miltenyi Biotec) and cells were filtered using a 40 µm cell strainer (BD Falcon). The viability of the cells was assessed microscopically and by flow cytometry using a LIVE/DEAD Fixable Yellow Dead Cell Stain Kit (Invitrogen) and was ~84%. The single-cell suspension was processed for scRNA-seq and library preparation with the Seq-Well protocol^92^. Library pools with fewer than 100 cells were discarded and merged into one object. The Seurat v3.2 pipeline was used to further analyze the data. Genes in fewer than five cells in the dataset, as well as the mitochondrial genes *MT-RNR1* and *MT-RNR2*, were removed. Cells with fewer than 200 genes were discarded, whereas cells with <5% mitochondrial genes or <30% intronic reads were kept for further analysis. The data were log normalized and 2,000 variable genes were calculated for each sample for integration with Seurat’s Canonical Correlation Analysis algorithm^93^. The data were scaled, 50 principle components were selected and the data were clustered with 0.6 resolution. Cluster annotation revealed a low-quality cluster that was subsequently removed and the process (the calculation of variable genes, calculation of 30 principal components, clustering with 0.4 resolution) was repeated. Raw counts of the cells that passed all filtering were provided as input for the HLCA.

### HLCA core data collection

To accommodate data protection legislation, scRNA-seq datasets of healthy lung tissue were shared by dataset generators as raw count matrices, thereby obviating the need to share genetic information. Count matrices were generated using varying software (Supplementary Table 1). Previously published scRNA-seq data were partly realigned by the dataset generators: the raw sequencing data of four previously published studies were realigned to GRCh38 using Ensembl84 for the HLCA^5,6,25,40^. For two of these studies^5,6^, the Cell Ranger 3.1.0-based HLCA pipeline was used for realignment. For the remaining two^25,40^, data were processed as previously described^25,40^, but with the reference genome and genome annotation adapted to the HLCA (GRCh38; Ensembl84). All other datasets in the HLCA core were originally already aligned to GRCh38 (Ensembl84) except data from ref. ^22^ (GRCh38; Ensembl93) (Supplementary Table 1). For ref. ^6^, the count matrices provided had ambient RNA removed, as described previously.

### Metadata collection (HLCA core)

For all of the datasets from the HLCA core, a preformatted sample metadata form was filled out by the dataset providers for every sample, containing metadata such as the ID of the donor from whom the sample came, the donor’s self-reported ethnicity, the type of sample, the sequencing platform and so on (Supplementary Table 2). Ethnicity metadata were based on self-reported ethnicity for live donors or retrieved from medical records or assigned by the organ procurement team in the case of organ donors, as collected in the individual studies. For donor ethnicity, the following categories of self-reported ethnicity were used during metadata collection: Black, white, Latino, Asian, Pacific Islander and mixed. To conform to pre-existing 1,000 Genomes ancestry superpopulations^94^, these self-reported ethnicity categories were then harmonized with the superpopulation categories as follows: Black was categorized as African, white as European and Latino as admixed American, while keeping the category Asian (merging the superpopulations East Asians and South Asians as this granularity was missing from the collected self-reported ethnicity data) and keeping Pacific Islander, as this category did not correspond to any of the superpopulations but does constitute a separate population in HANCESTRO^95^. We refer to the resulting categories as harmonized ethnicity. Both self-reported ethnicity (as collected) and harmonized ethnicity per donor are detailed in Supplementary Table 2. Cell type annotations from dataset providers were included in all datasets. For tissue dissociation protocols, protocols were grouped based on: (1) enzyme(s) used for tissue dissociation; and (2) the digestion time in cases where large differences were observed between protocols (that is, cold protease protocols were split into two groups: 30–60 min versus overnight).

### General data preprocessing for the HLCA core

Patients with lung conditions affecting larger parts of the lung, such as asthma or pulmonary fibrosis, were excluded from the HLCA core and later added to the extended atlas. For the HLCA core, all matrices were gene filtered based on Cell Ranger Ensembl84 gene-type filtering^96^ (resulting in 33,694 gene IDs). Cells with fewer than 200 genes detected were removed (removing 2,335 cells and 21 extra erythrocytes with close to 200 genes expressed as these hampered SCRAN normalization; see below), along with genes expressed in fewer than ten cells (removing 5,167 out of 33,694 genes).

### Total count normalization with SCRAN

To normalize for differences in total UMI counts per cell, we performed SCRAN normalization^97^. Since SCRAN assumes that at least half of the genes in the data being normalized are not differentially expressed between subgroups of cells, we performed SCRAN normalization within clusters. To this end, we first performed total count normalization, by dividing each count by its cell’s total count and multiplying by 10,000. We then performed a log transformation using natural log and pseudocount 1. A PCA was subsequently performed. Using the first 50 principal components, a neighborhood graph was calculated with the number of neighbors set to *k* = 15. Data were subsequently clustered with Louvain clustering at a resolution of *r* = 0.5. SCRAN normalization was then performed on the raw counts, using the Louvain clusters as input clusters and with the minimum mean (library size adjusted) average count of genes to be used for normalization set to 0.1. The resulting size factors were used for normalization. For the final HLCA (and not the benchmarking subset), cells with abnormally low size factors (<0.01) or abnormally high total counts after normalization (>10 × 10^5^) were removed from the data (267 cells in total).

### Cell type reference creation and metadata harmonization

To harmonize cell type labels from different datasets in the HLCA core, a common reference was created to which original cell type labels were mapped (Supplementary Table 4). To accommodate labels at different levels of detail, the cell type reference was made hierarchical, with level 1 containing the coarsest possible labels (immune, epithelial and so on) and level 5 containing the finest possible labels (for example, naive CD4 T cells). Levels were created in a data-driven fashion, recursively breaking up coarser-level labels into finer ones where finer labels were available.

To map anatomical location to a 1D CCF score that could be used for modeling, a distinction was made between upper and lower airways. First, an anatomical coordinate score was applied to the upper airways, starting at 0 and increasing linearly (with a value of 0.5) between each of the following anatomical locations: inferior turbinate, nasopharynx, oropharnyx, vesibula and larynx. The trachea received the next anatomical coordinate score using the same linear increment as in the upper airways (a score of 2.5). In the lower airways, the coordinate score within the bronchial tree was based on the generation airway, with the trachea being the first generation and the total number of generations assumed to be 23 (ref. ^98^). The alveolar sac was assigned the coordinate score of the 23rd generation airway. The coordinate score of each generation airway was calculated by taking the log_2_ value of the generation and adding it to the score of the trachea. Using this methodology, the alveolus received an anatomical coordinate score of 7.02. To calculate the final CCF coordinate, the coordinate scores (ranging from 0 to 7.02) were scaled to a value between 0 (inferior turbinate) and 1 (alveolus). Samples were then mapped to this coordinate system using the best approximation of the sampling location for each of the samples of the core HLCA (Supplementary Table 3).

### Data integration benchmarking

For computational efficiency, benchmarking was performed on a subset of the total atlas, including data from ten studies split into 13 datasets (ref. ^22^ was split into 10xv1 and 10xv2 data; ref. ^25^ was split into 10xv2 and 10xv3 data; and the pooled data from ref. ^21^ and associated unpublished data were split into two based on the processing site). The data came from 72 donors, 124 samples and 372,111 cells. Preprocessing of the benchmarking data included the filtering of cells (minimum number of total UMI counts: 500) and genes (minimum number of cells expressing the gene: 5).

For integration benchmarking, the scIB benchmarking framework was used^99^ with default integration parameter settings unless otherwise specified. All benchmarked methods except scGen (that is, BBKNN, ComBat, Conos, fas^99^ tMNN, Harmony, Scanorama, scANVI, scVI and Seurat RPCA) were run at least twice: on the 2,000 most HVGs; and on the 6,000 most HVGs. Of these methods, all that did not require raw counts as input were run twice on each gene set: once with gene counts scaled to have a mean of 0 and standard deviation of 1; and once with unscaled gene counts. scVI and scANVI, which require raw counts as input, were not run on scaled gene counts. scGen was only tested on 2,000 unscaled HVGs. For HVG selection, first, HVGs were calculated per dataset using Cell Ranger-based HVG selection^100^ (default parameter settings: min_disp=0.5, min_mean=0.0125, max_mean=3, span=0.3, n_bins=20). Then, genes that were highly variable in all datasets were considered overall highly variable, followed by genes highly variable in all datasets but one, in all datasets but two and so on until a predetermined number of genes were selected (2,000 or 6,000 genes).

For scANVI and scVI, genes were subset to the HVG set and the resulting raw count matrix was used as input. For all other methods, SCRAN-normalized (as described above) data were used. Genes were then subset to the precalculated HVG sets. For integration of gene-scaled data, all genes were scaled to have mean of 0 and standard deviation of 1.

Two integration methods allowed for input of cell type labels to guide the integration: scGen and scANVI. As labels, level 3 harmonized cell type labels were used (Supplementary Table 4), except for blood vessel endothelial, fibroblast lineage, mesothelial and smooth muscle cells, for which we used level 2 labels. Since scGen does not accept unlabeled cells, cells that did not have annotations available at these levels (that is, cells annotated as cycling, epithelial, stromal or lymphoid cells with no further annotations; 4,499 cells in total) were left out of the benchmarking data.

The dataset rather than the donor of the sample was used as the batch parameter. The maximum memory usage was set to 376 Gb and all methods requiring more memory were excluded from the analysis. The quality of each of the integrations was scored using 12 metrics, with four metrics measuring the batch correction quality and eight metrics quantifying the conservation of biological signal after integration (Supplementary Fig. 1; metrics previously described^28^). Overall scores were computed by taking a 0.4:0.6 weighted mean of batch effect removal to biological variation conservation (bioconservation), respectively. Methods were ranked based on overall score (Supplementary Fig. 1).

### Splitting of studies into datasets

For integration of the data into the HLCA core, we first determined for which cases studies had to be split into separate datasets (which were treated as batches during integration). Reasons for possible splitting were: (1) different 10x versions used within a study (for example, 10xv2 versus 10xv3); or (2) the processing of samples at different institutes within a study. To determine whether these covariates caused batch effects within a study, we performed principal component regression^101^. To this end, we preprocessed single studies separately (total count normalization to median total counts across cells and subsequent PCA with 50 principal components). For each study, we then calculated the fraction of the variance in the first 50 principal components that could be explained (PC_expl_) by the covariate of interest (that is, 10x version or processing institute):

$${\rm{PC}}_{{\rm{expl}}} = \frac{{\mathop {\sum }\nolimits_{i = 1}^{50} \quad {\rm{var}}\left( {{\rm{cov}}} \right)}}{{\mathop {\sum }\nolimits_{i = 1}^{50} \quad {\rm{var}}\left( {{\rm{PC}}_i} \right)}}$$

where var(PC_*i*_|cov) is the variance in scores for the *i*th principal component across cells that can be explained by the covariate under consideration, based on a linear regression.

Then, 10x version or processing institute assignments were randomly shuffled between samples and PC_expl_ was calculated for the randomized covariate. This was repeated over ten random shufflings and the mean and standard deviation of PC_expl_ were then calculated for the covariate. If the nonrandomized PC_expl_ was more than 1.5 standard deviations above the randomized PC_expl_, we considered the covariate a source of batch effect and split the study into separate datasets. Thus, both Jain_Misharin_2021 and ref. ^22^ were split into 10xv1 and 10xv2; ref. ^25^ was split into 10xv2 and 10xv3; and ref. ^21^ and its pooled unpublished data were not split based on 10x version nor on processing location.

### Integration of HLCA core datasets with scANVI

For integration of the datasets into the HLCA core, coarse cell type labels were used as described for integration benchmarking (AT1, AT2, arterial endothelial cell, B cell lineage, basal, bronchial vessel 1, bronchial vessel 2, capillary, multiciliated, dendritic, fibroblast lineage, KRT5^−^KRT17^+^ epithelial, lymphatic endothelial cell, macrophages, mast cells, megakaryocytes, mesothelium, monocytes, neutrophils, natural killer/natural killer T cells, proliferating cells, rare, secretory, smooth muscle, squamous, submucosal secretory, T cell lineage, venous and unlabeled), except cells with lacking annotations were set to unlabeled instead of being removed. scANVI was run on the raw counts of the 2,000 most HVGs (calculated as described above), using datasets as the batch variable to enable the conservation of interindividual variation. The following parameter settings were used: number of layers: 2; number of latent dimensions: 30; encode covariates: True; deeply inject covariates: False; use layer norm: both; use batch norm: none; gene likelihood: nb; n epochs unsupervised: 500; n epochs semi-supervised: 200; and frequency: 1. For the unsupervised training, the following early-stopping parameters were used: early stopping metric: elbo; save best state metric: elbo; patience: 10; threshold: 0; reduce lr on plateau: True; lr patience: 8; and lr_factor: 0.1. For the semisupervised training, the following early-stopping parameter settings were used: early stopping metric: accuracy; save best state metric: accuracy; on: full dataset; patience: 10; threshold: 0.001; reduce lr on plateau: True; lr_patience: 8; and lr_factor: 0.1. The integrated latent embedding generated by scANVI was used for downstream analysis (clustering and visualization). For gene-level analyses (differential expression and covariate effect modeling), uncorrected counts were used.

### UMAP embedding and clustering

To cluster the cells in the HLCA core, a nearest neighbor graph was calculated based on the 30 latent dimensions that were obtained from the scANVI output, with the number of neighbors set to *k* = 30. This choice of *k*, while improving clustering robustness, could impair the detection of very rare cell types. Coarse Leiden clustering was done on the graph with a resolution of *r* = 0.01. For each of the resulting level 1 clusters, a new neighbor graph was calculated using scANVIs 30 latent dimensions, with the number of neighbors again set to *k* = 30. Based on the new neighbor graph, each cluster was clustered into smaller level 2 clusters with Leiden clustering at a resolution of *r* = 0.2. The same was done for levels 3 and 4 and (where needed) 5, with *k* set to 15, 10 and 10, respectively, and the resolution set to 0.2. Clusters were named based on their parent clusters and sister clusters (for example, cluster 1.2 is the third biggest subcluster (starting at 0) of cluster 1). For visualization, a 2D UMAP^102^ of the atlas was generated based on the 30 nearest neighbors graph.

### Calculating cluster entropy of cell type labels and donors

To quantify cluster cell type label disagreement for a specific level of annotation, the label Shannon entropy was calculated on the cell type label distribution per cluster as

$$- \mathop {\sum }\limits_{i = 1}^k p\left( {x_i} \right){\rm{log}}\left[ {p\left( {x_i} \right)} \right],$$

where *x*_1_…*x*_*k*_ are the set of labels at that annotation level and *p*(*x*_*i*_) is the fraction of cells in the cluster that was labeled as *x*_*i*_. Cells without a label at the level under consideration were not included in the entropy calculation. If <20% of cells were labeled at the level under consideration, the entropy was set to not available for the figures. The entropy of donors per cluster (that is, diversity of donors in a cluster) was calculated in the same way.

### Thresholds for high label/donor entropy and doublet clusters

To set a threshold for high label entropy, we calculated the label entropy of a hypothetical cluster with 75% of cells given one label and 25% of cells given another label, as a cluster with <75% of cells with the same label suggests substantial disagreement in terms of cell type labeling. Clusters with a label entropy above that level (0.56) were considered to have high label entropy. Six small clusters with high label entropy even at the coarsest level of annotation highlighted doublet populations (identified via simultaneous expression of lineage-specific marker genes; for example, expression of both epithelial (AT2) and stromal (smooth muscle) marker genes) not labeled as such in the original datasets. These clusters were removed from the HLCA core, bringing the total number of clusters to 94. To set a threshold for low donor entropy, we calculated the label entropy for a hypothetical cluster with 95% of cells from one donor and the remaining 5% of cells distributed over all other donors, as clusters with >95% of the cells from the same cluster could be considered single-donor clusters, possibly caused by remaining batch effects or by donor-specific biology that is difficult to interpret. Clusters with a donor entropy below that level (0.43) were considered clusters with low donor entropy.

### Rare cell type analysis

To determine how well rare cell types (ionocytes, neuroendocrine cells and tuft cells) were clustered together and separate from other cell types after integration, we calculated recall (the percentage of all cells annotated as a specific rare cell type that were grouped into the cluster) and precision (the percentage of cells from the cluster that were annotated as a specific rare cell type) for all level 3 clusters. Nested clustering on Harmony^29,102^ and Seurat’s RPCA^30^ output was done based on PCA of the corrected gene counts, recalculating the principal components for every parent cluster when performing clustering into smaller children clusters, with clustering performed as described above under ‘UMAP embedding and clustering’. The level 3 clusters with the highest sensitivity for each cell type are included in Supplementary Fig. 3b.

### Manual cell type annotation

Re-annotation of cells in the HLCA core was done by six investigators with expertise in lung biology (E.M., M.C.N., A.V.M., L.-E.Z., N.E.B. and J.A.K.) based on original annotations and differentially expressed genes of the HLCA core clusters. Annotation was done per cluster, using finer clusters where these represented specific known cell types or states rather than donor-specific variation. Annotations of cell identities were hierarchical (as was the harmonized cell type reference) and each cluster was annotated at the finest known level, whereafter coarser levels could automatically be inferred (for example, a cell annotated as a CD8^+^ T cell was then automatically annotated as of T cell lineage at level 3, lymphoid cell lineage at level 2 and immune cell lineage at level 1). The number of cells per cell type is shown for all levels in Supplementary Table 5.

Mislabeling of original cells was identified by comparing final annotations with original harmonized labels and checking whether these were contradictory (and not only done at different levels of detail). Out of 61 final cell types, 18 included mostly mislabeled cells, 12 of which were previously known cell types. Despite consisting of mostly mislabeled cells from the original datasets, individual experts agreed on the annotation of these cell types: for all previously known cell types with a high proportion of mislabeled cells, the majority of experts agreed on the final annotation for the atlas, or only differed in the granularity of annotation.

### Marker gene selection

Marker genes were calculated based on per-sample, per-cell-type pseudo-bulks, calculating the mean (normalized and log-transformed) expression per pseudo-bulk for every gene. Pseudo-bulks were only calculated for a sample if it had at least ten cells of the cell type under consideration. An exception was made for cell types with fewer than 100 cells in total, for which the minimum number of cells per sample was set to 3. Pseudo-bulks rather than cell-level counts were used to ensure equal weighing of every sample in the differential expression test, thus statistically testing cell type-specific changes in expression that were significant across samples rather than cells. As pseudo-bulks represent the mean of a repeated draw from a single distribution, based on the central limit theorem, we expect pseudo-bulk gene counts to be normally distributed, and a *t*-test was therefore used to test differential gene expression, comparing a single cell type with all other cell types in the atlas (marker iteration 1). To further filter out differentially expressed genes that were not consistently expressed across samples, we applied a filtering step to remove genes expressed in <80% of the pseudo-bulks, or genes expressed in <50% of cells per pseudo-bulk (with the filtering based on the mean across pseudo-bulks). Similarly, to ensure specificity of gene expression, additional filtering was done to remove genes expressed in >20% of other pseudo-bulks. For many cell types, marker genes unique to a single cell type across the entire atlas could not be found. To nonetheless collect a robust and unique set of marker genes for every cell type, a hierarchical approach was taken, subsetting the atlas to four compartments (epithelial, endothelial, immune and stromal, for each of which a marker set was calculated) before calculating cell type-specific marker genes and filtering on uniqueness only within the compartment (marker iteration 2). When necessary, a second subsetting step was done, now subsetting to the next coarsest cell type set within the compartment (for example, lymphatic endothelial cells) and repeating the same procedure (marker iteration 3). Finally, filtering criteria were loosened for the remaining cell types for which no unique markers could be found in any of the iterations (marker iterations 4 and 5). Exact filtering parameters per iteration can be found in Supplementary Table 16. For lymphatic endothelial cell subtypes, one subtype contained sufficient cells for only a single sample, hampering a pseudo-bulk-based approach. Therefore, lymphatic endothelial cell subset markers (mature, differentiating and proliferating) were chosen based on known literature, after checking consistency with expression patterns observed in the HLCA lymphatic endothelial cells.

### Variance between individuals explained by covariates

To quantify the extent to which different technical and biological covariates correlated with interindividual variation in the atlas, we calculated the variance explained by each covariate for each cell type. We first split the data in the HLCA core by cell type annotation, merging substates of a single cell type into one (Supplementary Table 5; includes the number of cells per cell type). For every cell type, we excluded samples that had fewer than ten cells of the sample. We then summarized covariate values per sample for every cell type depending on the variable, taking the mean across cells from a sample for scANVI latent components (integration results), UMI counts per cell and fractions of mitochondrial UMIs, while for all other covariates (for example, BMI and tissue sampling method) each sample had only one value; therefore, these values were used.

Next, we performed principal component regression on every covariate, as described previously (see the section ‘Splitting of studies into datasets’), but now using scANVI latent component scores instead of principal component scores for the regression, yielding a fraction of latent component variance explained per covariate. Samples that did not have a value for a given covariate (for example, where the BMI was not recorded for the donor) were excluded from the regression. Categorical covariates were converted to dummy variables. Cell type–covariate pairs for which only one value was observed for the covariate were excluded from the analysis.

Quantification of the correlation or dependence between variables within a cell type and identification of the minimum number of samples needed to control for spurious correlation are described below.

### Covariate dependence for interindividual variance

To check the extent to which covariates correlated with each other, thereby possibly acting as confounders in the principal component regression scores, we determined dependence between all covariate pairs for every cell type. If at least one covariate was continuous, we calculated the fraction of variance in the continuous covariate that could be explained by the other covariate (dummying categorical covariates) and took the square root (equal to Pearson’s *r* for two continuous covariates). For two categorical covariates, if both covariates had more than two unique values, we calculated normalized mutual information between the covariates using scikit-learn^103^, since a linear regression between these two covariates is not possible.

### Finding the minimum number of samples for variance modeling

To control for spurious correlations between interindividual cell type variation and covariates due to low sample numbers, we assessed the relationship between sample number and mean variance explained across all covariates for every cell type. We found that for cell types sampled in fewer than 40 samples the mean variance explained across all covariates showed a high negative correlation with the number of samples (Supplementary Fig. 4a). We reasoned that for these cell types correlations between interindividual variation and our covariates were inflated due to undersampling. Moreover, we note that at lower sample numbers technical and biological covariates often strongly correlate with each other across donors (Supplementary Fig. 4c). This might lead to the attribution of true biological variation to technical covariates, and vice versa, complicating the interpretation of observed interindividual cell type variation. Consequently, we consider 40 a recommended minimum number of samples to avoid spurious correlations between observed interindividual variation and tested covariates, and excluded results from cell types with fewer samples.

### Cell type filtering covariate encoding for gene-level modeling

To select cell types for which covariate effects could be confidently modeled at the gene level, we followed the same procedure for every cell type: we filtered out all genes that were expressed in fewer than 50 cells and all samples that had fewer than ten cells of the cell type. We furthermore filtered out datasets with fewer than two donors and refrained from modeling categories in covariates that had fewer than three donors in their category for that cell type.

We encoded smoking status as a continuous covariate, setting never to 0, former to 0.5 and current to 1. Anatomical region was encoded into anatomical region CCF scores as described earlier. As we noted that changes from the nose to the rest of the airways and lungs were often independent from continuous changes observed in the lungs only, we encoded nasal as a separate covariate, setting samples from the nose to 1 and all others to 0. BMI and age were rescaled, such that the 10th percentile of observed values across the atlas was set to 0 and the 90th percentile was set to 1 (25 and 64 years for age, respectively, and 21.32 and 36,86 for BMI).

To determine whether covariance between covariates was low enough for modeling, we calculated the variance inflation factor (VIF) between covariates at the donor level. The VIF quantifies multicollinearity among covariates of an ordinary least squares regression and a high VIF indicates strong linear dependence between variables. If the VIF was higher than 5 for any covariate for a specific cell type, we concluded that covariance was too high and excluded that cell type from the modeling. As many cell types lacked sufficient representation of harmonized ethnicities other than European, including harmonized ethnicity in the analysis simultaneously decreased the samples that could be included in the analysis to only those with ethnicity annotations; hence, we excluded harmonized ethnicity from the modeling.

### Modeling gene-level interindividual variation and GSEA

To model the effects of demographic and anatomical covariates (sex, age, BMI, harmonized ethnicity, smoking status and anatomical location of the sample) on gene expression, we employed a generalized linear mixed model. We used sample-level pseudo-bulks (split by cell type), since the covariates modeled also varied at the sample or donor level and not at the cell level. Modeling these covariates at the cell level (that is, treating single cells as independent samples even when they come from the same sample) has been shown to inflate *P* values^104,105^. First, we split the lung cell atlas by cell type annotation, pooling detailed annotations into one subtype (for example, grouping all lymphatic endothelial cell subtypes into one) (Supplementary Table 5; includes the number of cells per cell type). Subsequent filtering, covariate encoding and exclusion of cell types due to covariate dependence are described above.

Gene counts were summed across cells for every sample, within cell type. Sample-wise sums (that is, pseudo-bulks) were normalized using edgeR’s calcNormFactors function, using default parameter settings. We then used voom^106^, a method designed for bulk RNA-seq that estimates observation-specific gene variances and incorporates these into the modeling. Specifically, we used a voom extension (differential expression testing with linear mixed models) that allows for mixed-effects modeling and modeled gene expression as:

$$\begin{array}{l}{\rm{log}}\left[ {{\rm{normcount}}} \right] \sim 1 + {\rm{age}} + {\rm{sex}} + {\rm{BMI}} + {\rm{smoking}} + {\rm{nose}} + {\rm{CCF}}\, {\rm{score}} \\+ \left( {1|{\rm{dataset}}} \right)\end{array}$$

where dataset is treated as a random effect to correct for dataset-specific changes in expression and all other effects are modeled as fixed effects. Resulting *P* values were corrected for multiple testing within every covariate using the Benjamini–Hochberg procedure.

To identify more systematic patterns across genes and changes happening at the gene set level, a gene set enrichment analysis was performed using correlation-adjusted mean-rank gene set tests^107^. The analysis was performed in R using the cameraPR function in the limma package^108^, with the differential expression test statistic. Gene Ontology biological process terms^109,110^ were tested separately for each comparison. These sets were obtained from MSigDB (version 7.1)^111^, as provided by the Walter and Eliza Hall Institute (https://bioinf.wehi.edu.au/MSigDB/index.html).

### Mapping of GWAS results to the HLCA cell types

To stratify GWAS results from several lung diseases by lung cell type, we applied stratified linkage disequilibrium score regression in single cells (sc-LDSC)^48^. sc-LDSC can link GWAS results to cell types by calculating, for each cell type, whether disease-associated variants are enriched in genomic regions of cell-type specific genes (i.e. the region of each gene and its surrounding base pairs), while taking into account the genetic signal of proximal linkage disequilibrium-associated regions. Here cell-type specific genes are defined as genes differentially expressed in the cell type of interest^48^. In contrast with simple enrichment testing of only significantly disease-associated genes from a GWAS among genes differentially expressed in a cell type, this method takes into account all SNPs included in the GWAS. Thus, consistent enrichment of weakly disease-associated genes (that would not individually pass significance tests) in a cell type could still lead to a significant association between the disease and the cell type. In this way, sc-LDSC provides more statistical power to detect associations between cell types and heritable phenotypes such as lung diseases.

To perform sc-LDSC on the HLCA, first a differential gene expression test was performed for every grouped cell type (Supplementary Table 5) in the HLCA using a Wilcoxon rank-sum test, testing against the rest of the atlas. The top 1,000 most significant genes with positive fold changes were stored as genes characterizing that cell type (cell type genes) and used as input for LDSC^48^. Gene coordinates of cell type genes were obtained based on the GRCh37.13 genome annotation. For SNP data (names, locations and linkage-related information), the 1000 Genomes European reference (GRCh37) was used, as previously described^48^. Only SNPs from the HapMap 3 project were included in the analysis. For identification of SNPs in the vicinity of cell type genes, we used a window size of 100,000 base pairs. Genes from X and Y chromosomes, as well as human leukocyte antigen genes, were excluded because of their unusual genetic architecture and linkage patterns. For linkage disequilibrium score calculation, a 1 cM window was used. Significance of the link between a phenotype and a cell type was calculated using LDSC^48^. *P* values yielded by LDSC were corrected for multiple testing for every disease tested using the Benjamini–Hochberg correction procedure. As a negative control, the analysis was performed with a GWAS of depression and no cell types were found to be significant (Supplementary Fig. 7). The numbers of cases and controls per GWAS study were as follows: *n* = 2,668 cases and 8,591 controls for IPF; *n* = 35,735 cases and 222,076 controls for COPD; *n* = 11,273 cases and 55,483 controls for lung adenocarcinoma; *n* = 321,047 individuals for lung function; *n* = 88,486 cases and 447,859 controls for asthma; and *n* = 113,769 cases and 208,811 controls for depression (used as negative control).

### Generating cell type signature matrices for deconvolution

To enable deconvolution of bulk expression data on the basis of the HLCA, HLCA cell type signature matrices were generated. One generic-purpose signature matrix was created per sublocation of the respiratory system (that is, one parenchyma, one airway and one nose tissue matrix; Supplementary Table 10). Additionally, a script to generate custom reference sets from the HLCA data is provided together with the HLCA code on GitHub (https://github.com/LungCellAtlas/HLCA) to tailor the deconvolution signature matrix to any specific research question.

Cell types were included in the bulk deconvolution signature matrix on the basis of cell proportions (constituting >2% of cells within samples of the corresponding tissue in the HLCA core). In addition, cell types were merged when they were deemed too transcriptionally similar. For each included cell type, 200 cells were randomly sampled from the HLCA core, while all cells were included for cell types with fewer than 200 cells present in the HLCA core. Cells were sampled from the matching anatomical location (for example, nose T cells rather than parenchymal T cells were used for the nose signature matrix). Signature matrices were constructed using CIBERSORTx^112^ (version 1.0) according to default settings, and no cross-platform batch correction was applied. The reference data were optimized by deconvolution of pseudo-bulk samples constructed from the HLCA core data, assessing deconvolution performance per included cell type based on the correlation of predicted proportions with ground truth composition (Supplementary Fig. 8a).

The following cell types were included in the deconvolution: endothelial cell arterial, endothelial cell capillary, lymphatic endothelial cell, basal and secretory (merged), multiciliated lineage, AT2, B cell lineage, innate lymphoid cell (ILC) natural killer and T cell lineage (merged), dendritic cells, alveolar macrophages, interstitial macrophages, mast cells, fibroblast lineage, smooth muscle, endothelial cell venous and monocytes (for the parenchyma); basal resting and suprabasal (merged), multiciliated lineage, club, goblet, dendritic cells, hillock like and T cell lineage (for the nose); and endothelial cell venous, CD4 T cells, fibroblasts, smooth muscle, basal and secretory (merged), multiciliated lineage, endothelial cell capillary, interstitial macrophages, B cell lineage, natural killer cells, CD8 T cells, dendritic cells, alveolar macrophages, mast cells and monocytes (for the airway). Capillary endothelial cells and interstitial macrophages (airway) were excluded from statistical testing due to poor performance in the benchmark. Venous endothelial cells and monocytes (parenchyma), hillock-like cells and T cell lineage cells (nose) and B cell lineage cells, natural killer cells, CD8 T cells, dendritic cells, alveolar macrophages, mast cells and monocytes (airways) were excluded from statistical testing due to >60% zero proportions.

### Deconvolution of bulk expression data using the HLCA core

The parenchymal signature matrix was used to deconvolve RNA expression data of samples from the Lung Tissue Database^52^ (GEO accession number GSE23546) using only lung tissue samples from patients with COPD GOLD stages 3 and 4 (*n* = 27 and 56, respectively) and matched controls (*n* = 281). The Lung Tissue Database dataset was run on the Rosetta/Merck Human RSTA Custom Affymetrix 2.0 microarray platform (HuRSTA-2a520709; GPL10379). As this platform has multiple probe sets for each gene, we focused on the probe sets that were derived from curated RefSeq records (with NM_ accession prefixes) when present to maximize the accuracy of the deconvolution. Where genes did not have probe sets based on curated RefSeq records or had multiple probe sets mapping to curated RefSeq records, the probe set with the highest average microarray intensity across samples was selected. Quantile normalization of the data and subsequent deconvolution were performed using CIBERSORTx. A Wilcoxon rank-sum test between control and GOLD stage 3/4 samples was performed to identify statistically significant compositional changes in advanced-stage COPD compared with control tissue. GOLD 3/4 and control samples were matched for age and smoking history. Cell types with >60% of samples predicted to have a proportion of zero were excluded from the Wilcoxon test, as the high number of tied ranks (zeros in both groups) would result in inflated *P* values. *P* values were multiple testing corrected using the Benjamini–Hochberg procedure.

The same procedure was followed for a dataset of nasal brush bulk RNA-seq samples from asthmatic donors pre- and postinhalation of corticosteroids (*n* = 54 and 26, respectively)^50^ and a dataset of airway biopsy bulk RNA-seq samples from asthmatic donors and controls (*n* = 95 and 38, respectively)^51^. As these consisted of RNA-seq data, no quantile normalization was applied.

### Extension of the HLCA core by mapping of new data

To map unseen scRNA-seq and single-nucleus RNA-seq data to the HLCA, we used scArches, our transfer learning-based method that enables mapping of new data to an existing reference atlas^71^. scArches trains an adaptor added to a reference embedding model, thereby enabling it to generate a common embedding of the new data and the reference, allowing reanalysis and de novo clustering of the joint data. The data to map were subsetted to the same 2,000 HVGs that were used for HLCA integration and embedding, and HVGs that were absent in the new data were set to 0 counts for all cells. Raw counts were used as input for scArches, except for the ref. ^40^ dataset, for which ambient RNA removal was run previously on the raw counts. Healthy lung data^40^ were split into two datasets: 3′ and 5′ based. Lung cancer data^41^ were also split into two datasets: 10xv1 and 10xv2.

The model that was learned previously for HLCA integration using scANVI was used as the basis for the scArches mapping. scArches was then run to train adaptor weights that allowed for mapping of new data into the existing HLCA embedding, using the following parameter settings: freeze-dropout: true; surgery_epochs: 500; train base model: false; metrics to monitor: accuracy and elbo; weight-decay: 0; and frequency: 1. The following early-stopping criteria were used: early stopping metric: elbo; save best state metric: elbo; on: full dataset; patience: 10; threshold: 0.001; reduce lr on plateau: True; lr patience: 8l and lr_factor: 0.1.

### Gene name harmonization

To enable cross-dataset gene-level analysis, harmonization of gene names from different datasets (using different reference genome builds and genome annotations; Supplementary Table 1) was necessary. Both annotation sources (for example, Ensembl or RefSeq) and annotation versions (for example, Ensembl release 84 or Ensembl release 91) contribute to the variation between different gene naming schemes. Therefore, both annotation sources and versions, including outdated ones, need to be taken into account to enable the mapping of all gene names to a single naming scheme.

For the harmonization of gene names, we aimed to map all original gene names to the target scheme HUGO Gene Nomenclature Committee gene name, corresponding to the Ensembl release 107 publication. To find the most likely match between each original gene name and a target gene name, we first downloaded Ensembl releases 79 to 107, which included for each release: (1) all Ensembl gene IDs from the downloaded release (for example, ENSG00000081237.21); (2) corresponding Ensembl transcript and protein IDs (for example, ENST00000442510.8 and ENSP00000411355.3); (3) matching Ensembl IDs from the previous release; (4) matching gene IDs from other genome annotation sources (for example, RefSeq); and (5) matching gene, transcript and protein identifiers from various external resources, such as UniProt, the HUGO Gene Nomenclature Committee and the Consensus Coding Sequence Project. We then constructed a graph, with each Ensembl ID, other genome annotation ID and external resource identifier represented by a single node per release. Nodes were then connected based on the matching (points 2–5) provided by Ensembl, weighing edges based on Ensembl similarity scores where available. For each original gene name from the HLCA datasets, the path with the lowest mean edge weight from that gene name to a gene name from the target names (Ensembl release 107) was selected to find the most likely matching gene name from the target (Supplementary Table 17). Genes for which no target could be found were excluded from downstream analysis. When multiple genes were matched with the same target gene name, counts from the original genes were summed.

### Identification of genes associated with common batch effects

To identify the genes most commonly exhibiting batch-specific expression, the HLCA was split by cell type and a differential expression analysis was performed (based on a Wilcoxon rank-sum test) in each cell type, comparing cells from one dataset (batch) with those from all other datasets and repeating this for all datasets. Datasets that had fewer than ten cells of the cell type or fewer than three samples with cells of the cell type were excluded from the test. For each test, genes were filtered such that only genes that were significantly upregulated were retained. Next, the fraction of included datasets in which a gene was significantly upregulated in the cell type (affected dataset fraction) was calculated for all genes. To find genes that were often batch effect associated across many cell types, the mean of the affected dataset fractions was calculated across cell types for each gene.

### Cell type label transfer from the HLCA core to new datasets

To perform label transfer from the HLCA core to the mapped datasets from the extended HLCA, we used the scArches *k* nearest neighbor-based label transfer algorithm^71^. Briefly, a *k* nearest neighbor graph was generated from the joint embedding of the HLCA core and the new, mapped dataset, setting the number of neighbors to *k* = 50. Based on the abundance and proximity in a cell’s neighborhood of reference cells of different types, the most likely cell type label for that cell was selected. Furthermore, a matching uncertainty score was calculated based on the consistency of reference annotations among the *k* nearest neighbors of the cell of interest

$$u_{c,y,{\rm{N}}_c} = 1 - p\left( {Y = y|X = c,{\rm{N}}_{c}}\! \right.{\left. \right)}$$

where *u*_*c*,y,Nc_ is the uncertainty score for a query cell *c* with transferred label *y*; N_*c*_ is its set of *k* nearest neighbors; and *p*(Y = *y*|X = *c*, N_*c*_) is the weighted (by edge weights) proportion of N_*c*_ with label *y*, as previously described^113^. Thus, high consistency in HLCA core annotations leads to low uncertainty scores and low consistency (that is, mixing of distinct reference annotations) leads to high uncertainty scores. For label transfer to lung cancer and healthy, spatially annotated projected data (Fig. 5b and Extended Data Fig. 7g), cells with an uncertainty score above 0.3 were set to unknown.

Disagreement between original labels and transferred annotations (that is, transferred annotations with high certainty but not matching the original label) in the data from ref. ^40^ highlighted three different cases: annotations not included in the mapped data (for example, preterminal bronchiole secretory cells, which were labeled as club and goblet in the mapped data; these may not be incorrect label transfers but cannot be verified by label comparison alone); cell types that are part of a continuum, with cutoffs between cell types chosen differently in the reference than in the projected data (for example, macrophage subtypes); and cell types missing in the HLCA core that have high transcriptional similarity to other cell types that are present in the HLCA, which was observed for several finely annotated immune cell identities. For example, γδ T cells, ILCs, megakaryocytes, natural killer T cells and regulatory T cells were not annotated in the HLCA core, as these cell types could not be distinguished with confidence in the integrated object and were often lacking in the constituent datasets. Indeed, cell types from the T cell/ILC/natural killer lineages are known to be particularly difficult to annotate using transcriptomic data only^16^. Therefore, cells annotated with these labels in the projected dataset were largely incorrectly annotated as CD4^+^ T cells, CD8^+^ T cells and natural killer cells through label transfer (Fig. 5b and Extended Data Fig. 6e)

### Calibration of uncertainty cutoff for classifying as unknown

For the extended atlas, we calibrated the uncertainty score cutoff by determining which uncertainty levels indicate possible failure of label transfer. To determine the uncertainty score at which technical variability from residual batch effects impairs correct label transfer, we evaluated how label transfer performed at the level of datasets, as these predominantly differ in experimental design. To determine an uncertainty threshold indicative of possible failure of label transfer, we harmonized original labels for 12 projected datasets^54,58,59,64,66^ (one unpublished: Duong_lungMAP_unpubl) and assessed the correspondence between original labels with the transferred annotations. Only cells with level 3 or 4 original annotations were considered, as these levels represent informative annotations while not representing the finest detail. Level 5 annotations will often display high uncertainty levels due to high annotation granularity rather than remaining batch effects. To assess the optimal uncertainty cutoff for labeling a new cell as unknown, we used these results to generate a receiver operating characteristic curve. We chose a cutoff around the elbow point, keeping the false positive rate below 0.5 (uncertainty cutoff = 0.2; true positive rate = 0.879; false positive rate = 0.495) to best distinguish correct from incorrect label transfers (Supplementary Fig. 10a). False positives were either due to incorrect label transfer or incorrect annotations in the original datasets. Cells with an uncertainty higher than 0.2 were set to unknown.

### Identifying clusters with spatially annotated cell types

The ref. ^40^ study of healthy lung included cell type annotations based on matched spatial transcriptomic data. Many of these annotations were not present in the HLCA core. To determine whether these spatial cell types could still be recovered after mapping to the HLCA core, we looked for clusters specifically grouping these cells. We focused on six spatial cell types: perineurial nerve-associated fibroblasts; endoneurial nerve-associated fibroblasts; immune-recruiting fibroblasts; chondrocytes; myelinating Schwann cells; and nonmyelinating Schwann cells. As these cell types were often present at very small frequencies, we performed clustering at different resolutions to determine whether these cells were clustered separately at any of these resolutions. We clustered at resolutions of 0.1, 0.2, 0.5, 1, 2, 3, 5, 10, 15, 20, 25, 30, 50, 80 and 100, with the number of neighbors set to *k* = 30 for resolutions under 25 and *k* = 15 for resolutions of 25 or higher, to enable the detection of smaller clusters. Minimum recall (the percentage of cells with the spatial cell type annotation captured in the cluster) and minimum precision (the percentage of cells from ref. ^40^ in the cluster that had the spatial cell type annotation) were both set to 25%. The cluster with the highest recall was selected for every spatial cell type (unless this cluster decreased precision by >33% compared with the cluster with the second highest recall). If the precision of the next best cluster was doubled compared with the cluster with the highest recall and recall did not decrease by >20%, this cluster was selected.

### Disease signature score calculation

To learn disease-specific signatures based on label transfer uncertainty scores, cells from the mapped data with the same transferred label (either alveolar fibroblasts or alveolar macrophages) were split into low-uncertainty cells (<0.2) and high-uncertainty cells (>0.4), excluding cells between these extremes (for alveolar fibroblasts*, n* = 11,119 (<0.2) and *n* = 2,863 (>0.4); for alveolar macrophages, *n* = 1,770 (<0.2) and *n* = 577 (>0.4)). We then performed a differential expression analysis on SCRAN-normalized counts using a Wilcoxon rank-sum test with default parameters, comparing high- and low-uncertainty cells. The 20 most upregulated genes based on log-fold changes were selected after filtering out genes with a false discovery rate-corrected *P* value (using the Benjamini–Hochberg procedure) above 0.05 and genes with a mean expression below 0.1 in the high-uncertainty group. To calculate the score of a cell for the given set of genes, the average expression of the set of genes was calculated, after which the average expression of a reference set of genes was subtracted from the original average, as described previously^114^. The reference set consists of a randomly sampled set of genes for each binned expression value. The resulting score was considered the cell’s disease signature score.

### Cross-dataset analysis of IPF-associated cell states

To uncover the cell identities affected in IPF, label transfer uncertainty was analyzed for three mapped datasets from the extended HLCA^58,62,64^ that included both IPF and healthy samples. Cell types of interest were determined based on the largest increase in mean label transfer uncertainty in IPF compared with healthy samples, while checking for consistency in increments across the three datasets. This highlighted alveolar fibroblasts as the main cell type of interest. To find IPF-specific alveolar fibroblast states, all alveolar fibroblasts from the abovementioned datasets and two more IPF datasets^21,24^ (for which no healthy data were mapped, as these were already in the core) were clustered together with the alveolar fibroblasts from the HLCA core. For clustering, a *k* nearest neighbor graph was calculated on the joint scArches-derived 30-dimensional embedding space setting *k* = 30, after which the cells were clustered using the Leiden algorithm with a resolution of 0.3. The resolution was chosen such that datasets were not isolated in single clusters, thus avoiding clustering driven solely by dataset-specific batch effects. One cluster (cluster 5) was small (*n* = 460 cells) and displayed low donor entropy (0.17), indicating that it almost exclusively came from a single donor (96% of cells from HLCA core donor 390C). It was therefore excluded from further analysis. To perform differential gene expression analysis, gene counts were normalized to a total of 7,666 counts (the median number of counts across the HLCA) and then log transformed with a pseudocount of 1. Finally, a Wilcoxon rank-sum test was used on the normalized data to detect differentially expressed genes for cluster 0 (*n* = 6,765 cells versus a total of *n* = 14,731). The results were filtered such that genes expressed in <30% of cells of the cluster of interest were excluded, as well as genes that were expressed in >20% of cells outside of the cluster and genes with a multiple testing-corrected *P* value (using the Benjamini–Hochberg procedure) above 0.05 (Supplementary Table 14).

### Multidisease analysis

To investigate whether the HLCA can be used to identify disease-associated cell states shared across multiple diseases, MDMs from the HLCA core, together with all cells from the mapped datasets labeled as MDMs based on label transfer, were jointly analyzed. Datasets and diseases with fewer than 50 MDMs were excluded from the analysis. The cells were subsequently clustered as described above for the cross-dataset IPF analysis. Finally, a Wilcoxon rank-sum test was used on the normalized data to detect differentially expressed genes per cluster (number of cells per cluster: *n* = 64,915 (cluster 0), 47,539 (cluster 1), 32,027 (cluster 2), 31,097 (cluster 3), 25,267 (cluster 4), 1,998 (cluster 5) and 307 (cluster 6)). The results were filtered as described above (Supplementary Table 15).

### Version information

The following tools and versions were used: R (version 4.1.1 for covariate modeling and version 4.0.3 for GSEA); edgeR (version 3.28.1); lme4 (version 1.1-27.1); LDSC (version 1.0.1); Limma (version 3.46.0); Scanpy (version 1.9.1); scArches (version 0.3.5); scIB (version 0.1.1); scikit-learn (version 0.24.1); and scvi-tools (scANVI; version 0.8.1).

### Reporting summary

Further information on research design is available in the Nature Portfolio Reporting Summary linked to this article.

## Online content

Any methods, additional references, Nature Portfolio reporting summaries, source data, extended data, supplementary information, acknowledgements, peer review information; details of author contributions and competing interests; and statements of data and code availability are available at 10.1038/s41591-023-02327-2.

## Supplementary information

Supplementary Information Supplementary Figs. 1–10.

Reporting Summary

Supplementary Tables 1–7, 11 and 13–16 Table 1. Overview and details of the datasets included in the HLCA. Table 2. Overview of the samples included in the HLCA, including experimental and donor metadata. Table 3. Encoding of a sample anatomical region into a 1D CCF score. Table 4. Mapping of datasets’ original cell type labels to the HLCA hierarchical reference annotation, which was built from coarsest annotation (level 1) to finest annotation (level 5, where available). Table 5. Overview of cell types in the final (manual) annotation in the HLCA, including their lower-level annotations (level 1: coarsest; level 5: finest). Table 6. Marker genes for each of the final cell types in the HLCA. Note that the marker gene set is hierarchical, with the first set of markers marking the cell type’s compartment (for example, ‘Stroma’ in the [ct]_marker_for column), the second set marking the coarse cell type (for example, ‘Fibroblast lineage’ in the [ct]_marker_for column) and the third set marking the actual cell type (for example, ‘Alveolar fibroblasts’ in the [ct]_marker_for column). [ct]_marker columns show the marker genes, while [ct]_marker_reference columns specify which part of the atlas was used as the reference for the differential expression analysis for those markers. For some cell types, only a single set of markers was sufficient to identify the cell type (for example, neuroendocrine). Table 7. Ranking of genes based on how often they are differentially expressed in different batches, across cell types. Table 11. Results of deconvolution of bulk expression data into cell type proportions, using the HLCA as a reference. Table 13. IPF disease signature genes for alveolar fibroblasts and alveolar macrophages. Table 14. Differentially expressed genes in alveolar fibroblast IPF-enriched cluster 0. Table 15. Differentially expressed genes per MDM cluster. Table 16. Filter parameter settings used for identifying robust marker genes for the HLCA cell types.

Supplementary Table 8 Results of modeling the effects of demographic variables on cell type-specific gene expression.

Supplementary Table 9 Gene set enrichment analysis results for modeling the effects of demographic variables on cell type-specific gene expression.

Supplementary Table 10 CIBERSORTx-generated cell type signature matrices for deconvolution of bulk expression data into cell type proportions.

Supplementary Table 12 Differential gene expression analysis comparing cells from patients with IPF with high cell type label transfer uncertainty and cells from the same patients with low label transfer uncertainty. Results for both alveolar fibroblasts and alveolar macrophages are included.

Supplementary Table 17 Mapping of gene names across HLCA datasets towards Ensembl release 107 gene names.

## Data Availability

The HLCA (raw and normalized counts, integrated embedding, cell type annotations and clinical and technical metadata) is publicly available and can be downloaded via cellxgene (https://cellxgene.cziscience.com/collections/6f6d381a-7701-4781-935c-db10d30de293). The HLCA core reference model and embedding for the mapping of new data to the HLCA can moreover be found on Zenodo (10.5281/zenodo.7599104). The original, published datasets that were included in the HLCA can also be accessed under GEO accession numbers GSE135893, GSE143868, GSE128033, GSE121611, GSE134174, GSE150674, GSE151928, GSE136831, GSE128169, GSE171668, GSE132771, GSE126030, GSE161382, GSE155249, GSE135851, GSE145926 and GSE178360, GSE227136, GSE158127, European Genome-phenome Archive study IDs EGAS00001004082, EGAS00001004344, EGAD00001005064 and EGAD00001005065 and URLs https://www.synapse.org/#!Synapse:syn21041850, https://data.humancellatlas.org/explore/projects/c4077b3c-5c98-4d26-a614-246d12c2e5d7, https://www.ncbi.nlm.nih.gov/projects/gap/cgi-bin/study.cgi?study_id=phs001750.v1.p1, https://www.nupulmonary.org/covid-19-ms2/?ds=full&meta=SampleName, https://figshare.com/articles/dataset/Single-cell_RNA-Seq_of_human_primary_lung_and_bronchial_epithelium_cells/11981034/1, https://covid19.lambrechtslab.org/downloads/Allcells.counts.rds, https://s3.amazonaws.com/dp-lab-data-public/lung-development-cancer-progression/PATIENT_LUNG_ADENOCARCINOMA_ANNOTATED.h5, https://github.com/theislab/2020_Mayr, https://static-content.springer.com/esm/art%3A10.1038%2Fs41586-018-0449-8/MediaObjects/41586_2018_449_MOESM4_ESM.zip, http://blueprint.lambrechtslab.org/#/099de49a-cd68-4db1-82c1-cc7acd3c6d14/*/welcome and https://www.covid19cellatlas.org/index.patient.html (see also Supplementary Table 1). GWAS summary statistics of COPD^46^ (GWAS catalog ID: GCST007692; database of Genotypes and Phenotypes (dbGaP) accession number: phs000179.v6.p2), IPF^115^ and lung adenocarcinoma^45^ (GWAS catalog ID: GCST004748; dbGaP accession number: phs001273.v3.p2) were made available via the dbGaP upon request. Summary statistics of lung function^47^ (GWAS catalog ID: GCST007429), asthma^44^ (GWAS catalog ID: GCST010043) and depression^116^ (used as a negative control; GWAS catalog ID: GCST005902) were publicly available.

## Code availability

The HLCA pipeline for processing the sequencing data for counting matrices, used for a subset of HLCA datasets (Methods), is available at https://github.com/LungCellAtlas/scRNAseq_pipelines. All further code used for the HLCA project can be found in the HLCA reproducibility GitHub repository (https://github.com/LungCellAtlas/HLCA_reproducibility). The landing page of the HLCA, including up-to-date links, can be found at https://github.com/LungCellAtlas/HLCA. Automated mapping to the HLCA and label transfer can be done with scArches^71^ at FASTGenomics (https://beta.fastgenomics.org/p/hlca) or using the code and tutorial in the HLCA mapping GitHub repository (https://github.com/LungCellAtlas/mapping_data_to_the_HLCA). Links to additional and updated platforms will be published on the HLCA landing page (see above). Automated mapping to the HLCA and label transfer with Azimuth^16,71^ (not shown in this manuscript) can be done at azimuth.hubmapconsortium.org. Label transfer with CellTypist^117^ (not shown in this manuscript) can be done at https://www.celltypist.org/models.
