# Supplementary material for: An integrated cell atlas of the lung in health and disease
Source: Nat Med. 2023 Jun 8;29(6):1563–77. doi: 10.1038/s41591-023-02327-2 (PMC10287567; doi:10.1038/s41591-023-02327-2)
Supplement: Supplementary file 1 — Supplementary Figs. 1–10. [file 41591_2023_2327_MOESM1_ESM.pdf]

nature medicine

Resource

https://doi.org/10.1038/s41591-023-02327-2

An integrated cell atlas of the lung in health and disease

In the format provided by the authors and unedited

SUPPLEMENTARY FIGURES

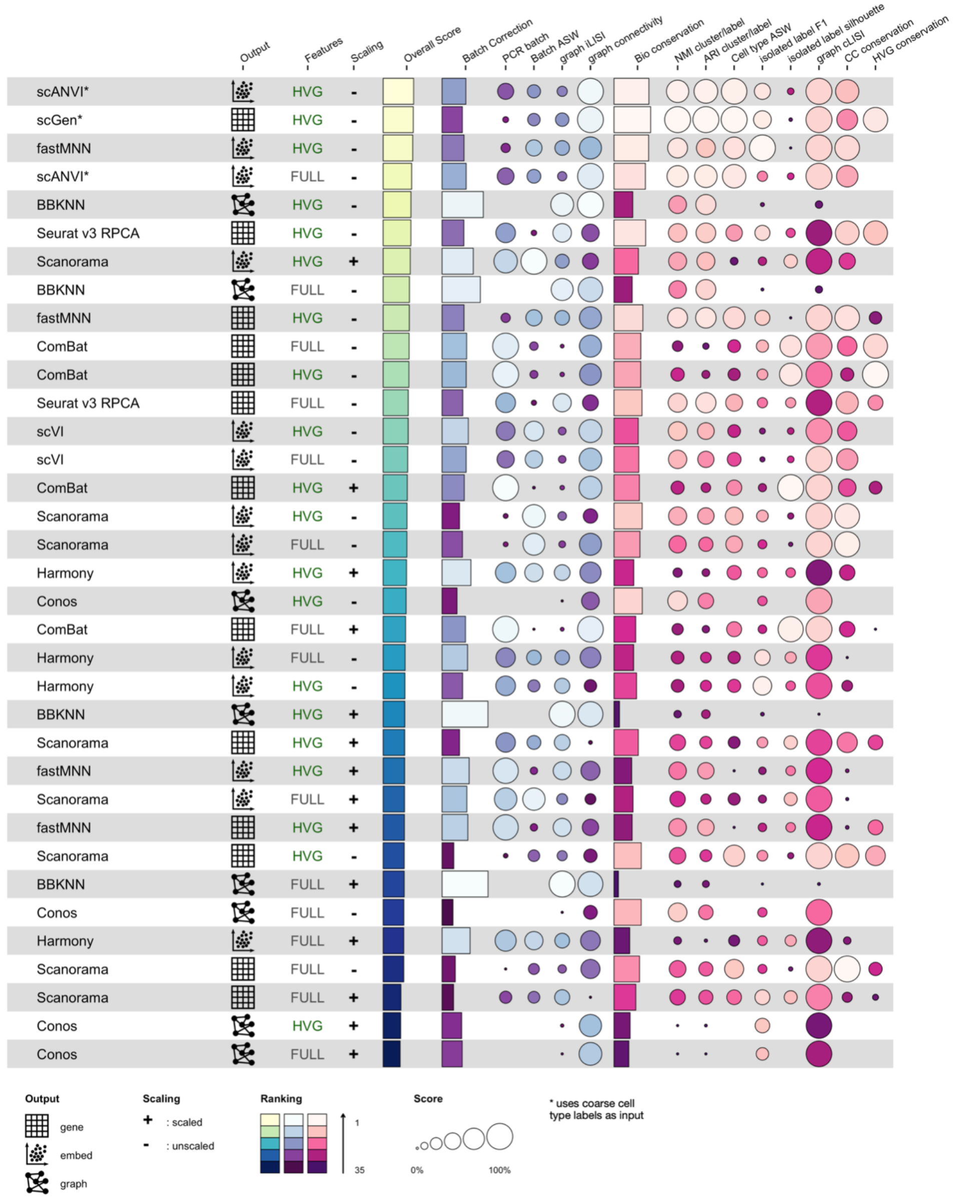

Supplementary figure 1. Results of dataset integration benchmarking. Rows represent methods tested, using a particular preprocessing and output. Preprocessing is summarized by “Features” (either HVG or FULL, corresponding to a gene selection of the 2000 or 6000 most highly variable genes, respectively) and “Scaling” (specifying whether or not gene values were scaled to mean 0 and standard deviation 1 across cells). Methods are sorted by overall score. Overall score is a weighted mean of the batch correction score and the bio conservation score, which in turn are a mean of the individual metrics within the category. The output column specifies whether a method has corrected gene counts, an integrated embedding, or an integrated graph as output. Scanorama and fastMNN were benchmarked twice, once with a corrected gene matrix as output, and once with a corrected embedding as output. scANVI and scGen were given coarse cell type labels as input, as indicated by the asterisk. fastMNN required too much memory on the full feature (6000 gene) data, therefore only the hvg results are included in the figure.

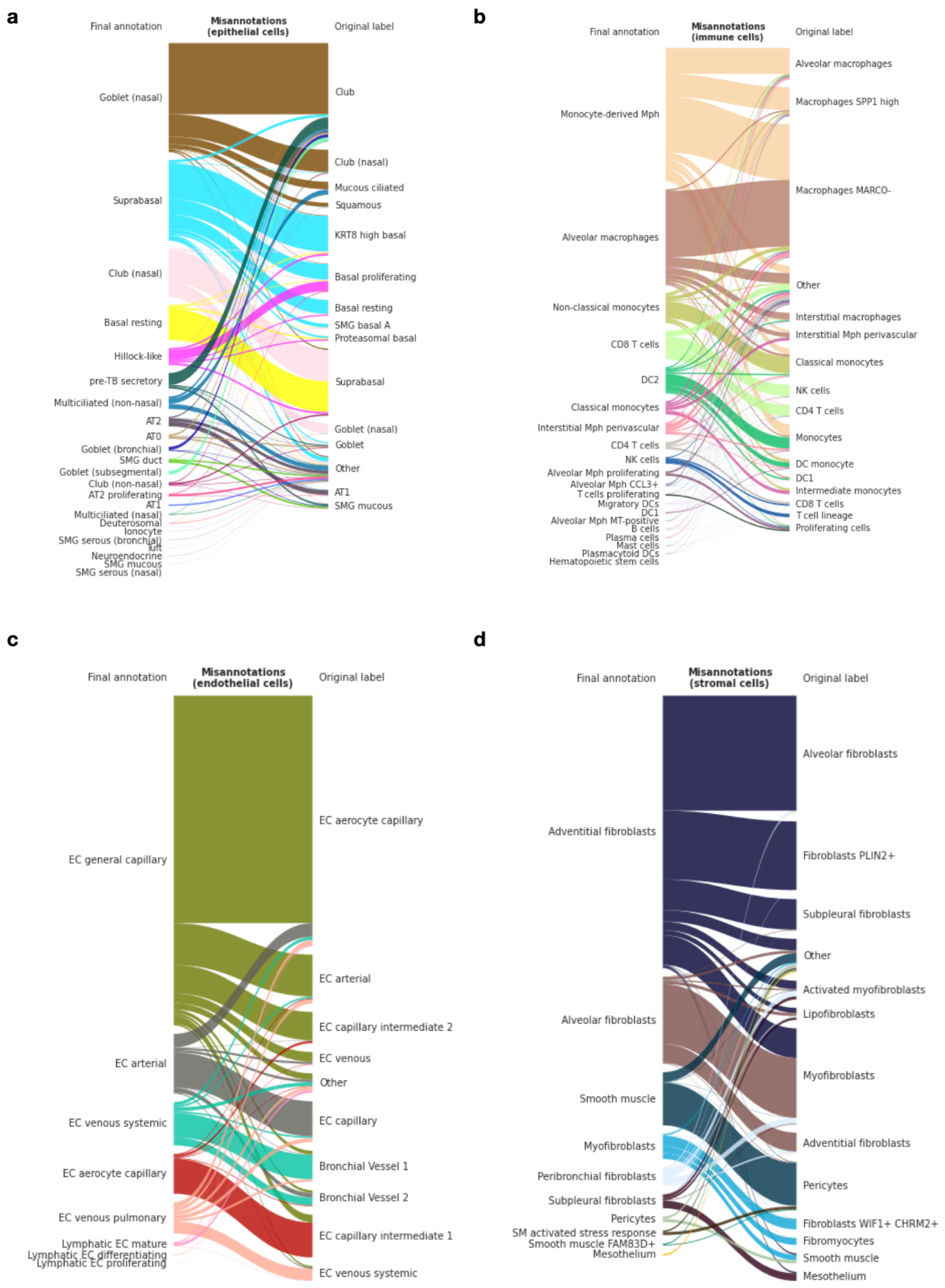

Supplementary figure 2. Final annotations and original labels of mislabeled cells. a, Final annotations (left) and original harmonized labels (right) of epithelial cells for which the final annotation and original label are contradictory, i.e. misannotated cells. Original labels that represent less than 1% of epithelial cells are set to “Other”. **b**, **c**, **d**, as **a** but for immune, endothelial and stromal cells, respectively. Mislabeled cells often differ from the final annotation at the finest level of annotation, but match at a lower level of granularity (e.g. adventitial instead of alveolar fibroblast). AT: alveolar type. TB: terminal bronchiole. SMG: submucosal gland. DC: dendritic cell. Mph: macrophage. NK: natural killer. MT: metallothionein. SM: smooth muscle. EC: endothelial cell.

a

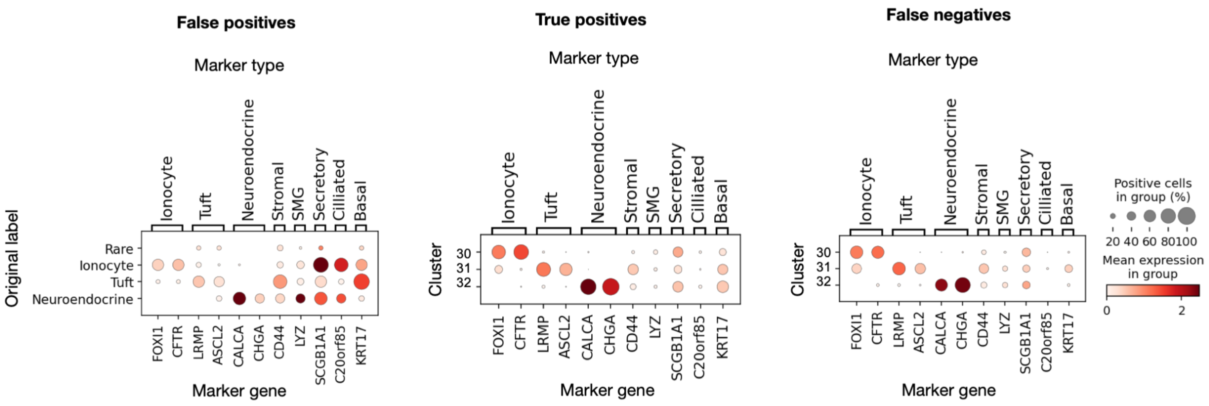

b

scANVI rare cell recall

cluster	% of ionocyte labels	% of tuft labels	% of NE cell labels
0.7.0	90.9	9.3	0.0
0.7.3	1.7	79.1	0.0
0.7.4	1.2	1.2	94.1

scANVI rare cell precision

cluster	Ionocyte precision	Tuft precision	NE precision
0.7.0	59.9	2.2	0.0
0.7.3	2.6	44.4	0.0
0.7.4	2.1	0.7	68.6

Harmony rare cell recall

cluster	% of ionocyte labels	% of tuft labels	% of NE cell labels
0.10.0	55.8	22.1	3.9
2.1.0	0.8	18.6	1.0
0.10.1	1.2	0.0	90.2

Harmony rare cell precision

cluster	Ionocyte precision	Tuft precision	NE precision
0.10.0	36.8	5.2	1.1
2.1.0	0.0	0.1	0.0
0.10.1	2.3	0.0	69.7

RPCA (Seurat) rare cell recall

cluster	% of ionocyte labels	% of tuft labels	% of NE cell labels
0.4.1	83.1	29.1	1.0
0.0.0	2.5	65.1	2.0
0.4.2	1.2	2.3	91.2

RPCA (Seurat) rare cell precision

cluster	Ionocyte precision	Tuft precision	NE precision
0.4.1	9.0	1.1	0.0
0.0.0	0.0	0.1	0.0
0.4.2	2.2	1.5	68.9

Supplementary figure 3. Rare cell identification and recovery for three different integration methods. a, Rare cell marker expression among different cell groups in the HLCA core. Markers of other cell types are included to show possible mis-annotations. Left: marker expression of cells originally labeled as rare, but which did not fall in one of the three rare cell clusters of the HLCA ("false positives"), subdivided by original label. Middle: Marker expression of cells originally labeled as rare, and which fell in one of the three rare cell clusters in the HLCA ("true positives"), subdivided by cluster. Right: marker expression of cells that were not originally labeled as rare, but that nonetheless were classified in one of the three rare cell clusters of the HLCA core ("false negatives"), subdivided by cluster. 78%, 76% and 96% of ionocytes, tuft, and neuroendocrine cells respectively were originally correctly labeled, and the final annotation increased the number of datasets in which these rare cells were detected up to three-fold to 10, 7 and 9 datasets, respectively. SMG: submucosal gland. **b**, Recall and precision of rare cell types in distinct clusters for three different integration methods: scANVI, Harmony and Seurat's RPCA. Results are shown for the best-performing preprocessing for each method, and based on the benchmarking data (12 datasets from the HLCA core). Recall (i.e. the percentage of cells with a specific label that are present in the cluster under consideration) and precision (i.e. the percentage of cells from a cluster labeled as the cell type under consideration) are shown for the three level 3 clusters with the highest recall of ionocytes, tuft, and NE cells respectively. NE: neuroendocrine.

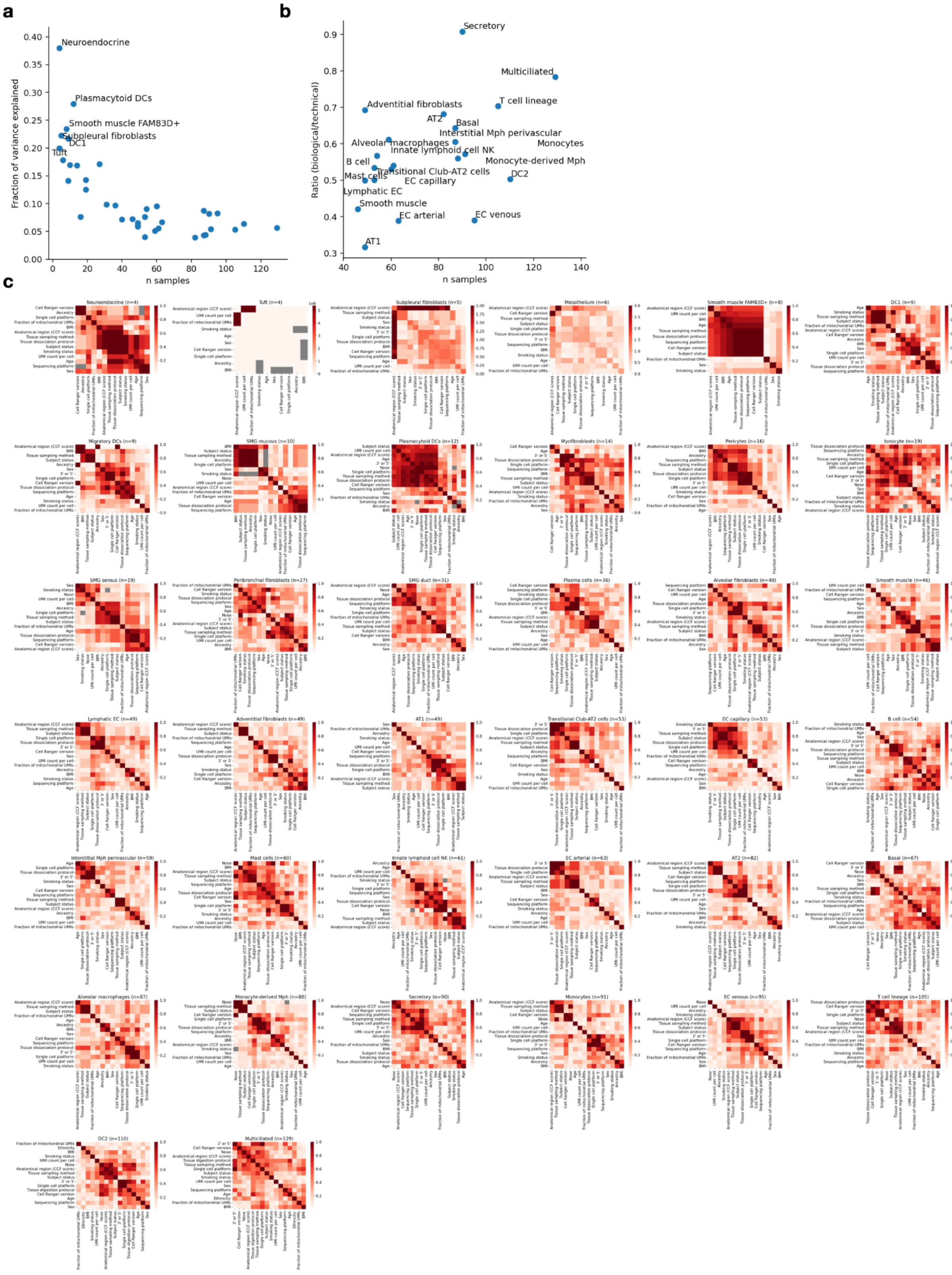

a
Fraction of variance explained
n samples
Neuroendocrine
Plasmacytoid DCs
Smooth muscle FAM83D+
Subpleural fibroblasts
DC1
Tuft
b
Ratio (biological/technical)
n samples
Secretory
Multiciliated
T cell lineage
Adventitial fibroblasts
AT2
Basal
Interstitial Mph perivascular
Alveolar macrophages
Monocytes
Innate lymphoid cell NK
B cell
Monocyte-derived Mph
Transitional Club-AT2 cells
Mast cells
DC2
EC capillary
Lymphatic EC
Smooth muscle
EC arterial
EC venous
AT1
c
Neuroendocrine (n=4)
Tuft (n=4)
Subpleural fibroblasts (n=5)
Mesothelium (n=6)
Smooth muscle FAM83D+ (n=8)
DC1 (n=9)
Migratory DCs (n=9)
SMG mucous (n=10)
Plasmacytoid DCs (n=12)
Myofibroblasts (n=14)
Pericytes (n=16)
Ionocyte (n=19)
SMG serous (n=19)
Peribronchial fibroblasts (n=27)
SMG duct (n=31)
Plasma cells (n=36)
Alveolar fibroblasts (n=40)
Smooth muscle (n=46)
Lymphatic EC (n=49)
Adventitial fibroblasts (n=49)
AT1 (n=49)
Transitional Club-AT2 cells (n=53)
EC capillary (n=53)
B cell (n=54)
Interstitial Mph perivascular (n=59)
Mast cells (n=60)
Innate lymphoid cell NK (n=61)
EC arterial (n=63)
AT2 (n=82)
Basal (n=87)
Alveolar macrophages (n=87)
Monocyte-derived Mph (n=88)
Secretory (n=90)
Monocytes (n=91)
EC venous (n=95)
T cell lineage (n=105)
DC2 (n=110)
Multiciliated (n=129)

Supplementary figure 4. Correlation of technical and biological covariates with number of samples, and with each other. **a**, Relation between the number of samples in which a cell type was observed, and the mean fraction of inter-sample variance explained per covariate, across technical and biological covariates. After n=40, these two variables become independent. Inter-sample variance was calculated based on the scANVI-integrated embedding, taking the mean score of each latent dimension across cells, for every sample for every cell type. **b**, The ratio of mean variance explained per biological covariate over that explained per technical covariate, for different cell types (y-axis), and its association with the number of samples in which a cell type was observed (x-axis) (Pearson r: 0.47, p=0.03). **c,** Correlation between covariates for every cell type, calculated at sample level. The square root of the fraction of variance from one covariate that could be explained by the other covariate through linear regression is shown (equivalent of Pearson correlation coefficient r for two continuous covariates). If both covariates were categorical and had more than two categories, normalized mutual information was calculated instead. AT: alveolar type. DC: dendritic cells. EC: endothelial cells. Mph: macrophages. NK cells: natural killer cells. SMG: submucosal gland.

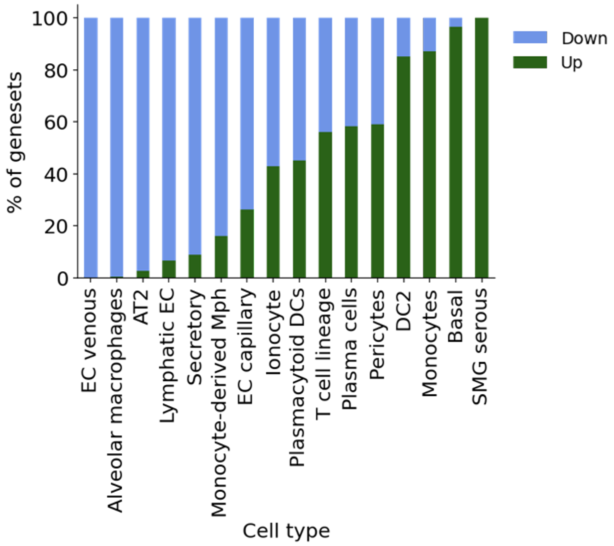

Supplementary figure 5. Proportion of significantly up- and down-regulated gene sets with BMI per cell type. Only cell types with at least 10 gene sets significantly associated with BMI are shown. DC: dendritic cells. EC: endothelial cells. Mph: macrophages. SMG: submucosal gland.

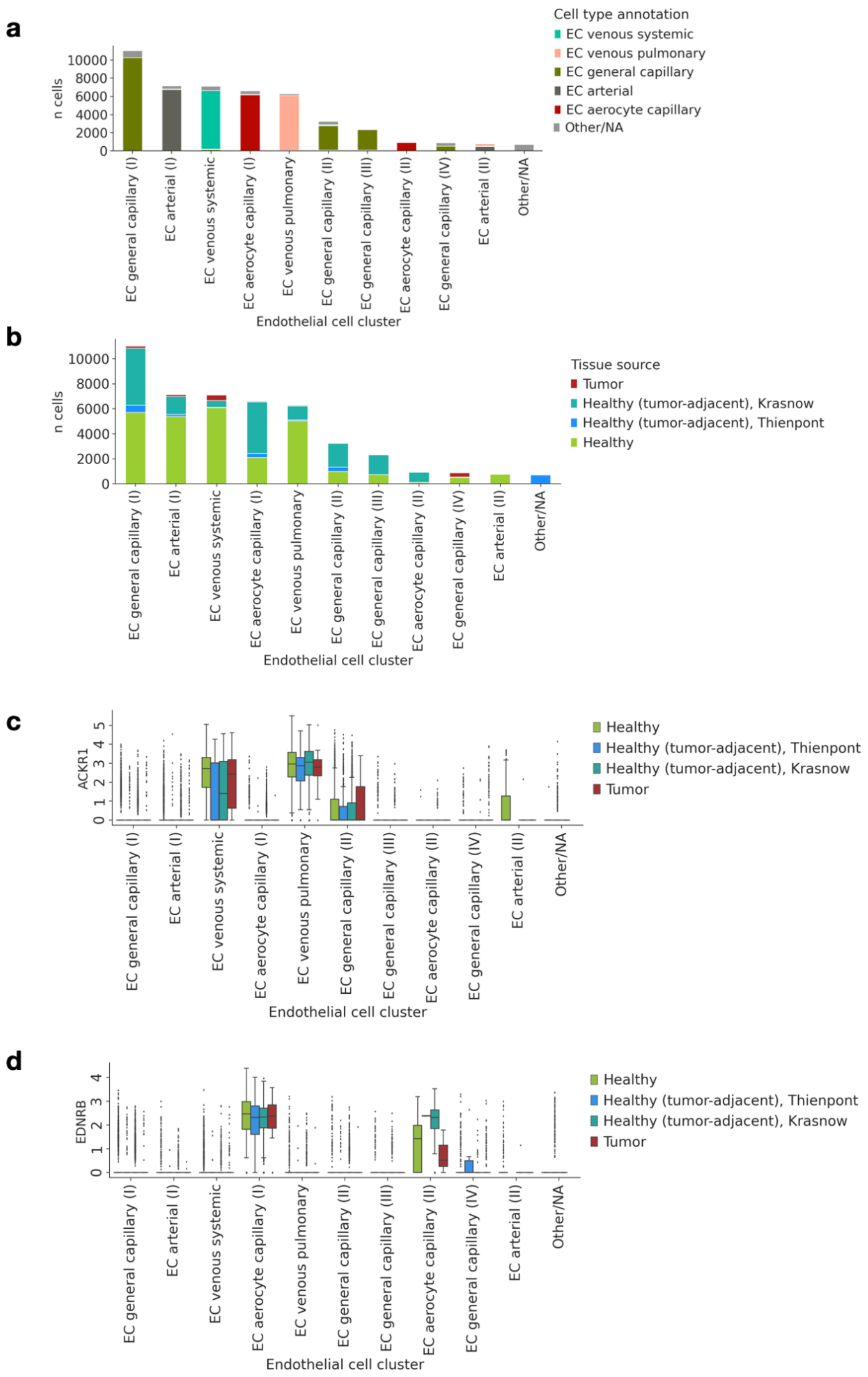

Supplementary figure 6. Endothelial cell clustering and marker expression in the joint embedding of unseen lung cancer data and the HLCA core. a, Cell type composition of EC clusters from the joint embedding of the HLCA core and the mapped lung cancer data. Final HLCA core annotations are shown, with cells from the cancer data as well as HLCA core annotations with fewer than 50 cells set to “Other/NA”. Clusters are named by their dominant cell type. **b**, Tissue source composition of EC clusters. Tissue source is either tumor, healthy (but from tumor-adjacent tissue), or healthy (from donors without lung cancer). Healthy tumor-adjacent is split by study, including the Krasnow study from the HLCA core (with non-tumorous tissue from lung cancer patients) for comparison. **c**, *ACKR1* expression in EC

clusters, split by tissue source. Boxes show median and interquartile range of expression. Cells with counts more than 1.5 times the interquartile range away from the high and low quartile are considered outliers and plotted as points. Whiskers extend to the furthest non-outlier point. **d**, same as **c**, but now showing *EDNRB* expression. EC: endothelial cell. For **a-d**, n cells per group is: EC general capillary (I): 10906, EC arterial (I): 7379, EC venous systemic: 7161, EC aerocyte capillary (I): 6574, EC venous pulmonary: 6318, EC general capillary (II): 3440, EC general capillary (III): 2859, EC aerocyte capillary (II): 930, EC general capillary (IV): 781, EC arterial (II): 689.

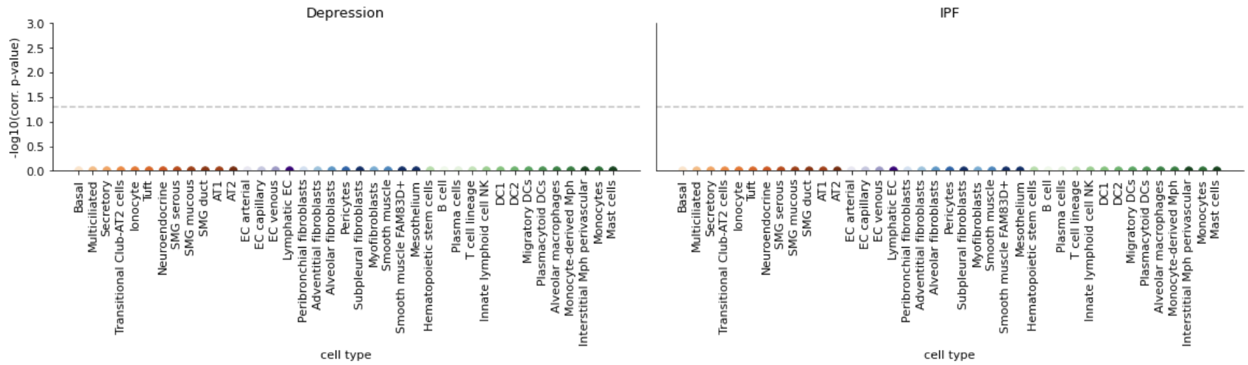

Supplementary figure 7. Association of lung cell types with depression and idiopathic pulmonary fibrosis. Analysis of a GWAS of depression (left) was included as a negative control for the analysis of **fig. 5d**. IPF data (right) likely included too few study donors to reach statistical significance in this analysis (**Methods**). Horizontal dashed line indicates significance threshold alpha=0.05. p-values were calculated using LD score regression (**Methods**) and multiple-testing-corrected with the Benjamini-Hochberg procedure. AT: alveolar type. SMG: submucosal gland. EC: endothelial cell. NK: natural killer. DC: dendritic cell. Mph: macrophages.

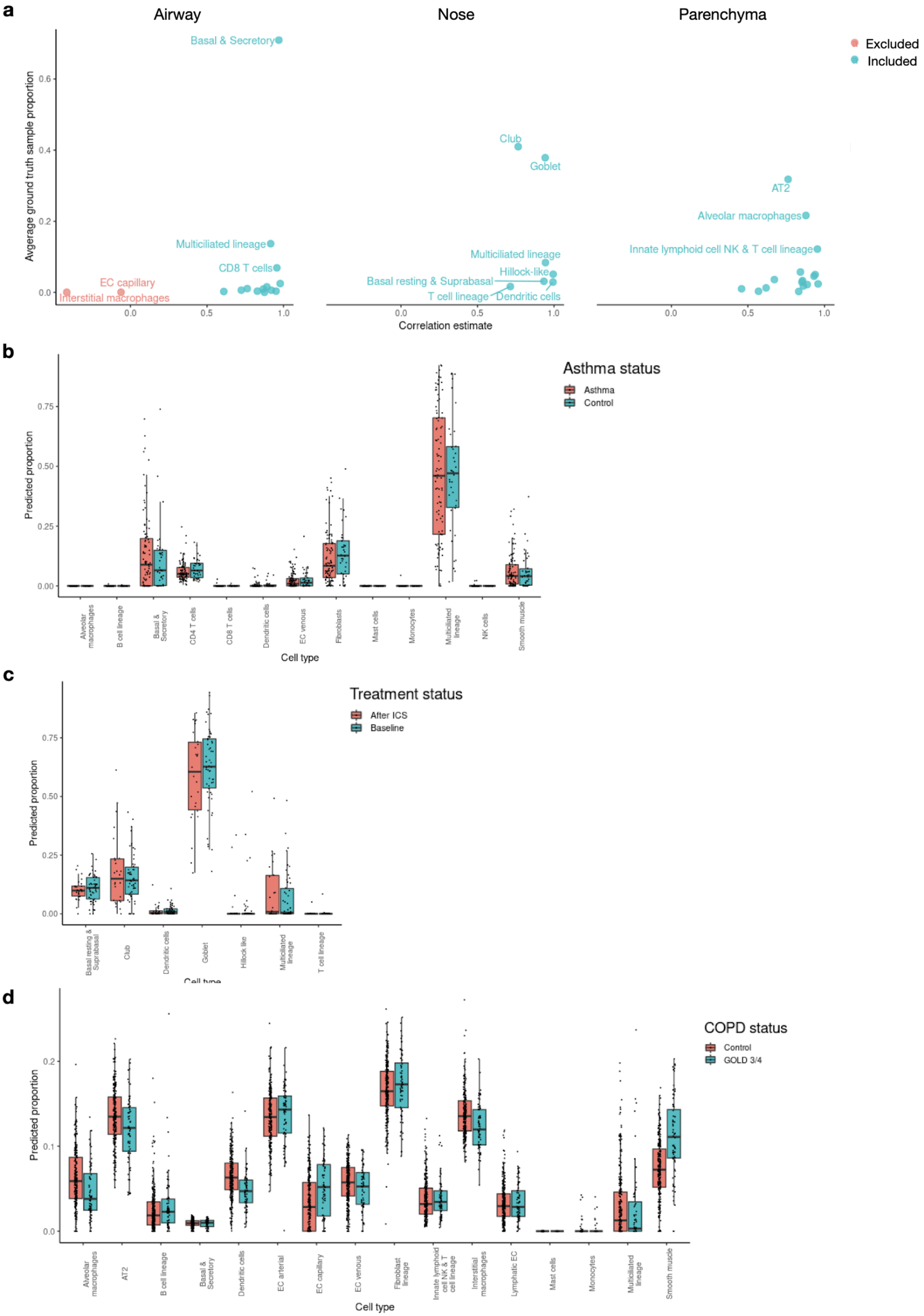

a
Airway
Nose
Parenchyma
Excluded
Included
Avgerage ground truth sample proportion
Basal & Secretory
Multiciliated lineage
CD8 T cells
EC capillary
Interstitial macrophages
Club
Goblet
Multiciliated lineage
Hillock-like
Basal resting & Suprabasal
T cell lineage
Dendritic cells
AT2
Alveolar macrophages
Innate lymphoid cell NK & T cell lineage
Correlation estimate
b
Asthma status
Asthma
Control
Predicted proportion
Alveolar macrophages
B cell lineage
Basal & Secretory
CD4 T cells
CD8 T cells
Dendritic cells
EC venous
Fibroblasts
Mast cells
Monocytes
Multiciliated lineage
NK cells
Smooth muscle
Cell type
c
Treatment status
After ICS
Baseline
Predicted proportion
Basal resting & Suprabasal
Club
Dendritic cells
Goblet
Hillock like
Multiciliated lineage
T cell lineage
Cell type
d
COPD status
Control
GOLD 3/4
Predicted proportion
Alveolar macrophages
AT2
B cell lineage
Basal & Secretory
Dendritic cells
EC arterial
EC capillary
EC venous
Fibroblast lineage
Innate lymphoid cell NK & T cell lineage
Interstitial macrophages
Lymphatic EC
Mast cells
Monocytes
Multiciliated lineage
Smooth muscle
Cell type

Supplementary figure 8. Deconvolution of bulk expression data into cell type proportions using the HLCA as a reference. a, Correlation of deconvolution-based cell type proportions with true proportions in HLCA-derived pseudo-bulks. Y-axis shows the mean ground truth proportion per cell type in HLCA-based pseudo-bulks, while the x-axis shows the correlation between deconvolution-based estimated cell type proportions versus true proportions in HLCA-based pseudo-bulks. All cell types displaying low correlation between deconvolution results and ground truth were present at low proportions. Cell types excluded from deconvolution based on low correlation with ground truth, per anatomical location (airway, nose and parenchyma) are shown in red, cell types included are shown in blue. **b**, Predicted cell type proportions in bronchial brush samples from donors with asthma (n=95) versus control donors (n=38), based on deconvolution of bulk expression data using the HLCA as a reference. **c**, Predicted cell type proportions of nasal scrapings from donors with asthma before (n=54) and after (n=26) inhalation of corticosteroids. **d**, Predicted cell type proportions in lung resections from severe COPD (GOLD stage 3 or 4, n=83) versus non-COPD control donors (n=281). Boxes show median and interquartile range of proportions. Samples with proportions more than 1.5 times the interquartile range away from the high and low quartile are considered outliers and plotted as points. Whiskers extend to the furthest non-outlier point. AT: alveolar type. EC: endothelial cell. NK: natural killer. COPD: chronic obstructive pulmonary disease.

a

	n cells in projected data	% of all cells	Mean precision (%)	Total recall (%)	Clustering resolution	N clusters
Chondrocytes	42	0.006	90.0	85.7	80	1
Endoneurial nerve-ass. fibroblasts	35	0.005	-	-	-	-
Immune-recruiting fibroblasts	59	0.008	74.2	39.0	80	1
Perineurial nerve-ass. fibroblasts	31	0.004	84.2	51.6	100	1
Schwann (myelinating)	7	0.001	41.1	100	20	1
Schwann (non-myelinating)	29	0.004	-	-	-	-

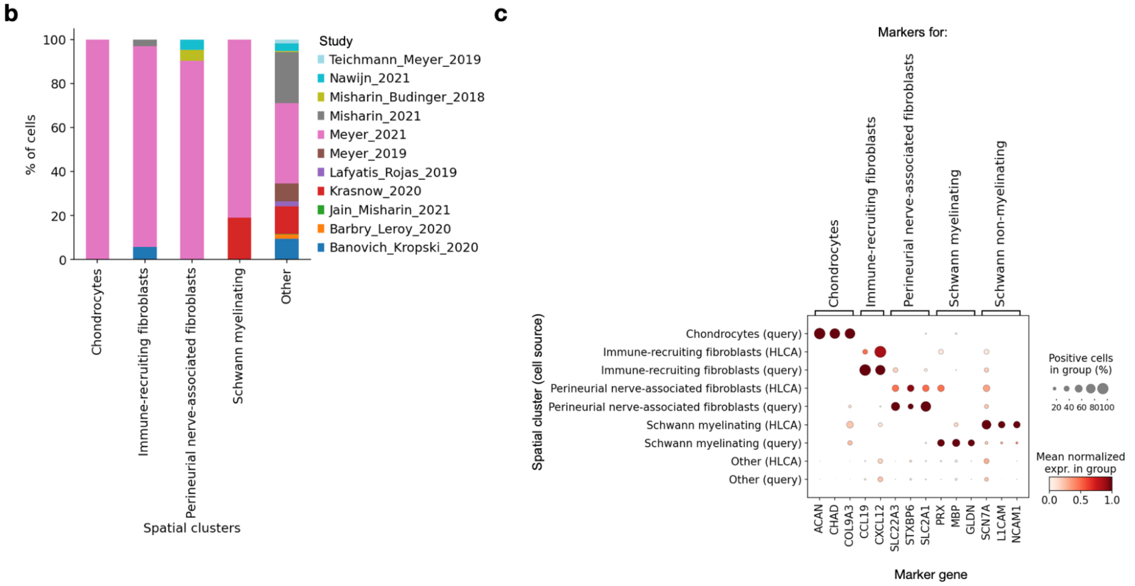

Supplementary figure 9. Identification of spatial-location-based cell types in the HLCA core based on mapping of spatially labeled data. a, Precision (i.e. the percentage of cells from a cluster labeled as the cell type under consideration) and recall (i.e. the percentage of cells with a specific label that are present in the cluster under consideration) of spatial cell types in spatial clusters. Precision and recall were calculated among cells of the mapped data only. Clustering resolution at which the cluster was identified, and number of clusters per spatial cell type is also shown. % of all cells specifies the percentage of cells with the label among cells of both the projected data and the HLCA core. **b**, Composition of spatially annotated clusters in terms of study from which the cells came. **c**, Marker expression of spatially annotated cell type markers across spatially annotated clusters, splitting clusters into cells from the HLCA core, and cells from the newly mapped data ("query"). Markers for non-myelinating Schwann cells are also included, as HLCA cells from the myelinating Schwann cell cluster rather exhibit marker expression of non-myelinating Schwann cells. Gene expression was normalized to range, within the stromal cell subset, from 0 to 1.

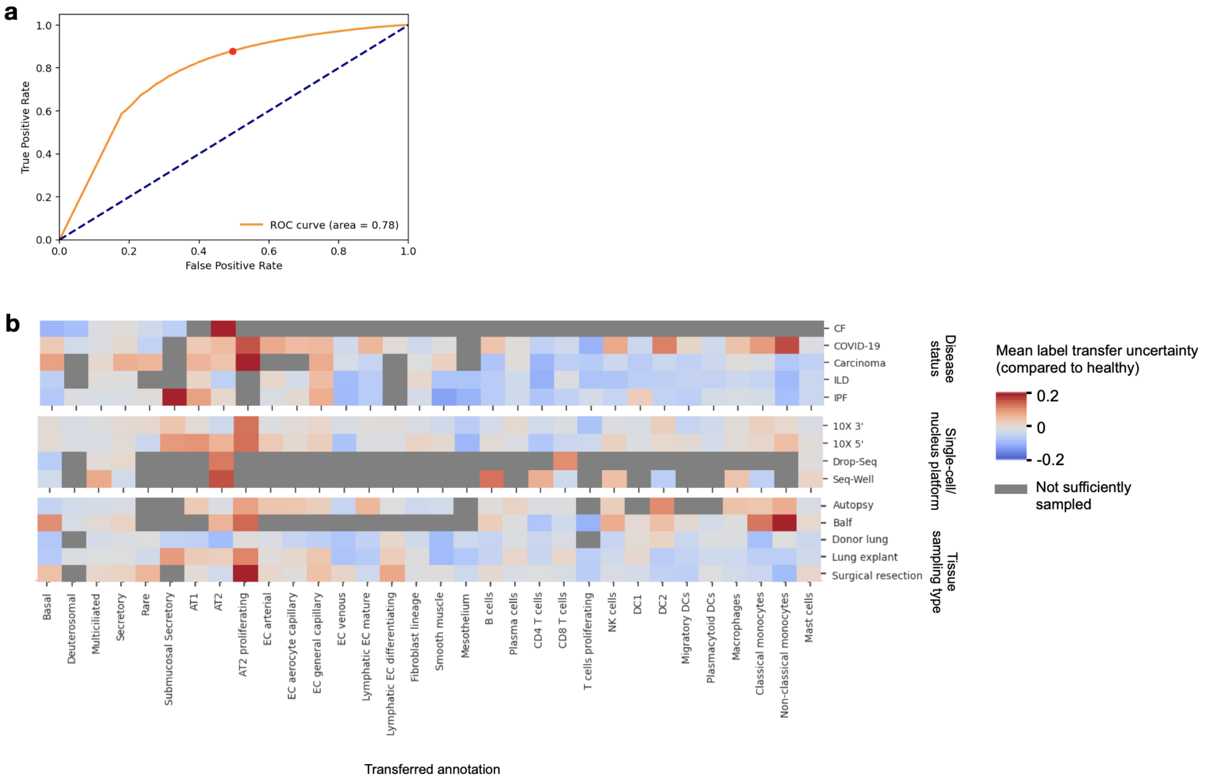

Supplementary figure 10. Label transfer uncertainty threshold setting and uncertainties per experimental feature and cell type. a, Calibration of label transfer uncertainty cutoff**.** ROC curve of label transfer accuracy across 12 datasets. The true and false positive rate of the chosen cutoff point (0.2), below which transferred labels will be considered low uncertainty, are shown as a red point on the ROC curve. **b**, Label transfer uncertainty per cell type across different experimental features. Label transfer uncertainty of cell types is shown for categories of three features (disease status, single nucleus/cell platform, tissue sampling type), as compared to uncertainty in healthy cells. For every category and for each cell type, the mean uncertainty across datasets from that category was calculated, using per-dataset means and splitting up datasets with samples from more than one category. The difference between cell type uncertainty from each category and those of healthy datasets is shown. Where mean uncertainty was higher than 0.25 even in healthy, coarser parent labels were included (e.g. for macrophages) instead of the finest cell type annotations. Datasets with fewer than 20 cells of a cell type are excluded for that cell type. When no dataset sampled enough cell types, the plot is masked in gray. Values higher than 0.2 or lower than -0.2 are cut off to 0.2 and -0.2, respectively.
