# Supplementary material for: An integrated cell atlas of the lung in health and disease
Source: Nat Med. 2023 Jun 8;29(6):1563–77. doi: 10.1038/s41591-023-02327-2 (PMC10287567; doi:10.1038/s41591-023-02327-2)
Supplement: Supplementary file 2 — Reporting Summary [file 41591_2023_2327_MOESM2_ESM.pdf]

nature portfolio

Corresponding author(s): Malte Luecken, Fabian Theis

Last updated by author(s): Mar 29, 2023

Reporting Summary

Nature Portfolio wishes to improve the reproducibility of the work that we publish. This form provides structure for consistency and transparency in reporting. For further information on Nature Portfolio policies, see our Editorial Policies and the Editorial Policy Checklist.

Statistics

For all statistical analyses, confirm that the following items are present in the figure legend, table legend, main text, or Methods section.

n/a	Confirmed	
☐	☒	The exact sample size (*n*) for each experimental group/condition, given as a discrete number and unit of measurement
☒	☐	A statement on whether measurements were taken from distinct samples or whether the same sample was measured repeatedly
☐	☒	The statistical test(s) used AND whether they are one- or two-sided<br>*Only common tests should be described solely by name; describe more complex techniques in the Methods section.*
☐	☒	A description of all covariates tested
☐	☒	A description of any assumptions or corrections, such as tests of normality and adjustment for multiple comparisons
☐	☒	A full description of the statistical parameters including central tendency (e.g. means) or other basic estimates (e.g. regression coefficient) AND variation (e.g. standard deviation) or associated estimates of uncertainty (e.g. confidence intervals)
☐	☒	For null hypothesis testing, the test statistic (e.g. *F*, *t*, *r*) with confidence intervals, effect sizes, degrees of freedom and *P* value noted<br>*Give P values as exact values whenever suitable.*
☒	☐	For Bayesian analysis, information on the choice of priors and Markov chain Monte Carlo settings
☒	☐	For hierarchical and complex designs, identification of the appropriate level for tests and full reporting of outcomes
☐	☒	Estimates of effect sizes (e.g. Cohen's *d*, Pearson's *r*), indicating how they were calculated

Our web collection on statistics for biologists contains articles on many of the points above.

Software and code

Policy information about availability of computer code

Data collection	No software was used for data collection.
Data analysis	All code is made public on GitHub, as also specified in the manuscript. https://github.com/LungCellAtlas/HLCA_reproducibility.<br>Software versions used:<br>R 4.1.1 (covariate modeling)<br>edgeR: 3.28.1<br>lme4: 1.1-27.1<br>LDSC: 1.0.1<br>Limma: 3.46.0, R 4.0.3 (GSEA)<br>Scanpy: 1.9.1<br>scArches: 0.3.5<br>scIB: 0.1.1<br>scikit-learn: 0.24.1<br>scvi-tools (scANVI): 0.8.1

For manuscripts utilizing custom algorithms or software that are central to the research but not yet described in published literature, software must be made available to editors and reviewers. We strongly encourage code deposition in a community repository (e.g. GitHub). See the Nature Portfolio guidelines for submitting code & software for further information.

Data

Policy information about availability of data

All manuscripts must include a data availability statement. This statement should provide the following information, where applicable:

- Accession codes, unique identifiers, or web links for publicly available datasets
- A description of any restrictions on data availability
- For clinical datasets or third party data, please ensure that the statement adheres to our policy

There are no restrictions on data availability. The HLCA is fully public.
Data Availability Statement
The HLCA (raw and normalized counts, integrated embedding, cell type annotations and clinical and technical metadata) is publicly available and can be downloaded via cellxgene:
https://cellxgene.cziscience.com/collections/6f6d381a-7701-4781-935c-db10d30de293
The HLCA core reference model and embedding for mapping of new data to the HLCA can moreover be found on Zenodo, doi: 10.5281/zenodo.7599104.

The original, published datasets that were included in the HLCA can also be accessed under GEO accession numbers GSE135893, GSE143868, GSE128033, GSE121611, GSE134174, GSE150674, GSE151928, GSE136831, GSE128169, GSE171668, GSE132771, GSE126030, GSE161382, GSE155249, GSE135851, GSE145926, GSE178360, EGA study IDs EGAS00001004082, EGAS00001004344, EGAD00001005064, EGAD00001005065, and under urls https://www.synapse.org/#!Synapse:syn21041850, https://data.humancellatlas.org/explore/projects/c4077b3c-5c98-4d26-a614-246d12c2e5d7, https://www.ncbi.nlm.nih.gov/projects/gap/cgi-bin/study.cgi?study_id=phs001750.v1.p1, https://www.nupulmonary.org/covid-19-ms2/?ds=full&meta=SampleName, https://figshare.com/articles/dataset/Single-cell_RNA-Seq_of_human_primary_lung_and_bronchial_epithelium_cells/11981034/1, https://covid19.lambrechtslab.org/downloads/Allcells.counts.rds, https://s3.amazonaws.com/dp-lab-data-public/lung-development-cancer-progression/PATIENT_LUNG_ADENOCARCINOMA_ANNOTATED.h5, https://github.com/theislab/2020_Mayr, https://static-content.springer.com/esm/art%3A10.1038%2Fs41586-018-0449-8/MediaObjects/41586_2018_449_MOESM4_ESM.zip, http://blueprint.lambrechtslab.org/#/099de49a-cd68-4db1-82c1-cc7acd3c6d14/*/welcome, https://www.covid19cellatlas.org/index.patient.html (see also Supplementary Data Table 1).
GWAS summary statistics of COPD(GWAS catalog ID: GCST007692, dbGaP accession number: phs000179.v6.p2), IPF, and of lung adenocarcinoma(GWAS catalog ID: GCST004748, dbGaP accession number: phs001273.v3.p2) were made available on dbGap upon request. Summary statistics of lung function (GWAS catalog ID: GCST007429), of asthma (GWAS catalog ID: GCST010043), and of depression (used as negative control, GWAS catalog ID: GCST005902) were publicly available.

Human research participants

Policy information about studies involving human research participants and Sex and Gender in Research.

Reporting on sex and gender

For each of the previously unpublished datasets included in the paper, the methods state whether sex was self-reported or assigned based on a medic's report. Disaggregated data of subjects' sex is available in the publicly available human lung cell atlas, which includes per-sample (and per-cell) metadata. Information about sex can moreover be found in Supplementary Data table 2, which includes per-sample metadata. Consent was obtained for obtaining and sharing patient data for each study, as indicated in the methods.
Effects of sex on cell type transcriptomes were modeled, for which results are shown in figure 4, as well as in Supplementary data table 8 and 9.
Overall male/female proportions among subjects included in the HLCA core are shown in figure 2a and specified in the caption (60% male, 40% female).

Population characteristics

This information can be found in Supplementary Data table 2 (sex, age, BMI, smoking history, lung disease). Statistics for the HLCA core are moreover stated in the text: "These datasets include samples from 107 individuals, with diversity in age, sex, ethnicity (harmonized as detailed in Methods), BMI, and smoking status (fig. 2a)." and in the caption of figure 2a: "Donors show diversity in ethnicity (harmonized metadata proportions 65% European, 14% African, 2% Admixed American, 2% mixed, 2% Asian, 0.4% Pacific Islander, 14% unannotated, see Methods)".
Further notes on the encoding of ethnicity can be found in the methods:
"Ethnicity metadata was based on self-reported ethnicity for live donors, or retrieved from medical records or assigned by the organ procurement team in case of organ donors, as collected in the individual studies. For donor ethnicity, the following categories of self-reported ethnicity were used during metadata collection: black, white, latino, asian, pacific islander, and mixed. To conform to pre-existing 1000 genomes ancestry “superpopulations”, these self-reported ethnicity categories were then harmonized with the superpopulation categories as follows: black was categorized as African, white as European, latino as admixed American, while keeping the category “Asian” (merging superpopulations “East Asians” and “South Asians'' as this granularity was missing from the collected self-reported ethnicity data), and keeping “Pacific Islander”, as this category did not correspond to any of the superpopulations but does constitute a separate population in HANCESTRO. We refer to the resulting categories as “harmonized ethnicity”. Both self-reported ethnicity as collected and harmonized ethnicity per donor are detailed in Supplementary Data Table 2."

Recruitment

Recruitment was done in individual studies as published, recruitment for unpublished data:

Banovich_Kropski_2020: Primary tissue was obtained from the Donor Network of Arizona or Tennessee Donor Services. All samples were collected from declined organ donors who were also consented for research. Only lungs with no known lung disease were used in this study.

Barbry_unpubl: Our IPF volunteers patients were selected from a prospective cohort of 180 IPF patients.
Patients involved in this paper respected all these Inclusion and non-inclusion criteria.
Inclusion criteria
Age > 18 years
Diagnosis of idiopathic pulmonary fibrosis made less than 5 years ago on scannographic and/or histological criteria and validated in an interstitial pathology consultation meeting according to the ATS/ERS/JRS/ALAT 2018 recommendations (Raghu G, Remy-Jardin M, Meyers JL, Richeldi L, Ryerson CJ, Lederer DJ, et al. Diagnosis of idiopathic pulmonary fibrosis: an official ATS/ERS/JRS/ALAT clinical practice guideline. Am J Respir Crit Care Med 2018;198:e44-e68.).
GAP Index 1 or 2 (Ley, B. et al. A Multidimensional Index and Staging System for Idiopathic Pulmonary Fibrosis. Ann Intern Med 156, 684-U658 (2012))
FVC > 50% of theoretical
DLCO > 35% of theoretical
Non-smoker (active or passive) or ex-smoker of less than 20 pack-years and stopped for more than 5 years
No current acute pathology at inclusion
No symptoms suggestive of a progressive pathology being diagnosed
Patient with a chest CT scan in the year prior to inclusion
Woman of childbearing age using effective contraception
Patient with written consent
Non-inclusion criteria:
Recent ENT or bronchial infection (< 6 weeks)
Long-term systemic corticosteroid therapy regardless of the reason for prescription
Systemic corticosteroid therapy in the previous 3 months
Patient on long-term oxygen therapy
Chronic cardiovascular, neuro-psychic or metabolic pathology in progress, clinically significant or not controlled during the last 6 months
Other associated chronic respiratory pathology (COPD, asbestosis, bronchiectasis, etc.)
Patient on anti-platelet agents or other anticoagulant at risk of bleeding during sampling
Patient with a history of cancer in the previous 5 years, excluding basal cell disease
Patient with a history of clinically significant (i.e. recurrent or loss of consciousness) vagal discomfort
History of allergy or intolerance to xylocaine and/or propofol
History of significant epistaxis (i.e. recurrent epistaxis of any amount or at least one severe epistaxis)
Patient at risk of difficult intubation according to the criteria of the SFAR 2006 expert conference*.
Relationship between volunteer and investigator
Patient not socially insured
Mental disability
Pregnant woman (a urine test will be carried out for all women of childbearing age) or nursing mother
Vulnerable person (person deprived of administrative and legal freedom).
In addition to the respect of all these inclusion criteria, a nasal swab analysis was made for each patient at the beginning of the procedure and analyzed for viruses (tested for 22 pathogens (RespiFinder® 2Smart). Virus: Influenza A, Influenza B, Influenza A(H1N1)pdm09, RSV-A, RSV-B, Human Metapneumovirus, Rhinovirus/Enterovirus, Adenovirus, Parainfluenza-1, Parainfluenza-2, Parainfluenza-3, Parainfluenza-4, Bocavirus, Coronavirus NL63/HKU1, Coronavirus OC43, Coronavirus 229E, SARS-CoV-2, MERS CoV, Bacteria: Mycoplasma pneumoniae, Chlamydophila pneumoniae, Legionella pneumophila, Bordetella pertussis). None of the patients selected had inhaled treatment.

Duong_HuBMAP_unpubl: The dataset includes a single donor: an organ donor who was a 37 year-old black male with a history of marihuana, with no lung disease.

Jain_Misharin_2021: Healthy volunteers were recruited to match a cohort of patients with cystic fibrosis for the ongoing study at Northwestern University (PI Manu Jain). In both studies Dr. Misharin did not influence participant recruitment and did not introduce biases in sample selection.

Misharin_2020: Donor Lungs. Samples were collected in an opportunistic manner, based on sample availability and organ allocation to Northwestern Lung Transplant center. One donor was rejected for organ transplant. For the other donor, samples were collected during donor lung for size reduction during lung transplantation.

Nawijn_2021: Subjects and methods
Recruitment was performed through advertisements in the local newspaper.
Inclusion criteria:
• Age between 18 and 45 years old.
• Smoking history ≤2 packyears and no smoking during the 6 months before inclusion
• No history of asthma.
• No use of inhaled corticosteroids or β2-agonists for a period longer than 1 month in their lifetime and not during the 6 weeks before inclusion.
• No symptoms of wheeze, nocturnal dyspnea, or bronchial hyperresponsiveness.
• PC20 methacholine > 8 mg/ml, FEV1/FVC > 70% and FEV1 > 80% predicted.
Exclusion criteria
• FEV1 <1.2 L,
• Subjects must be able to adhere to the study visit schedule and other protocol requirements.
• A subject is not eligible to enter and participate if he has not signed and dated a written informed consent form prior to participation in the study.
• A subject is not eligible to enter and participate if he does not agree that we inform his general practitioner.
• Upper respiratory tract infection (e.g. colds), within 6 weeks.
• Serious acute infections (such as hepatitis, pneumonia or pyelonephritis) in the previous 3 months.
• Signs or symptoms of severe, progressive or uncontrolled renal, hepatic, hematologic, endocrine, pulmonary, cardiac, neurologic or cerebral disease.
• Malignancy within the past 5 years (except for squamous or basal cell carcinoma of the skin that has been treated with no

evidence of recurrence).
• Known recent substance abuse (drug or alcohol).
• Females of childbearing potential without an efficient contraception unless they meet the following definition of post-menopausal: 12 months of natural (spontaneous) amenorrhea or 6 months of spontaneous amenorrhea with serum FSH >40 mIU/mL or the use of one or more of the following acceptable methods of contraception:
a) Surgical sterilization (e.g. bilateral tubal ligation, hysterectomy).
b) Hormonal contraception (implantable, patch, oral, injectable).
c) Barrier methods of contraception: condom or occlusive cap (diaphragm or cervical/vault caps) with spermicidal foam/gel/cream/suppository.
d) Continuous abstinence.

Schiller_2021: Non-involved tissue from lung tumor resections was used. All fresh tissues from patients in a given timeframe without any specific selection criteria were included. Only patients with obvious chronic lung disease as comorbidity based on their lung function parameters prior to tumor resection were excluded.

Schultze_unpubl: Patients undergoing lung tumor resections. At Hannover Medical School, MHH, patients with lung cancer were recruited in the course of their operation, i.e. surgical tumor resection according to the ethical vote of the German Centre for Lung Research (DZL), ethical vote 7414 and data safety guidelines. There was no further bias in patients recruitment since the samples were collected as fresh native tissue following surgical tumor resection and availability of “surplus” adjacent non-malignant lung tissue, which was resected in parallel to the tumor tissue.

Tata_unpubl: Transplant donor tissues were collected from individuals that died from accidental death. Lungs were screened by PCR and antigen testing to exclude HIV, HCV, Burkholderia and other respiratory viruses. Sub-transplant quality transplant donor tissues were collected from individuals that died from accidental death.

Ethics oversight

Banovich_Kropski_2020: Vanderbilt IRB nos. 060165 and 171657 and Western IRB no. 20181836
Barbry_unpubl: CHU Nice, registered at clinicaltrials under reference NCT04529993.
Duong_HUBMaP_unpubl: brindl.urmc.rochester.edu/
Jain_Misharin_2021: Protocol was approved by Northwestern University IRB (STU00214826)
Misharin_2020: Protocol was approved by Northwestern University IRB (STU00212120).
Nawijn_2021: University Medical Center Groningen Institutional Review Board (ABR number NL69765.042.19)
Schiller_2021: local ethics committee of the Ludwig-Maximilians University of Munich, Germany (EK 333-10 and 382-10).
Schultze_unpubl: ethical approval of Hannover Medical School Nr. 7414, 2017
Tata_unpubl: Duke University Institutional Review Board (Pro00082379) and the University of North Carolina Biomedical Institutional Review board (03-1396).

Note that full information on the approval of the study protocol must also be provided in the manuscript.

Field-specific reporting

Please select the one below that is the best fit for your research. If you are not sure, read the appropriate sections before making your selection.

☒ Life sciences ☐ Behavioural & social sciences ☐ Ecological, evolutionary & environmental sciences

For a reference copy of the document with all sections, see nature.com/documents/nr-reporting-summary-flat.pdf

Life sciences study design

All studies must disclose on these points even when the disclosure is negative.

Sample size

All large, publicly available single-cell lung datasets were included in the HLCA where available. For the HLCA core, 10X data of non-diseased lung tissue was included. For the atlas extension, disease data was included as well, from any UMI-based single cell protocol. Furthermore, data from groups who offered to share unpublished data (data generation included in the methods, data included in the -publicly available-HLCA was included. No other data was generated for the HLCA.

Data exclusions

No data were excluded from the analysis. For the HLCA core, only data of control (i.e. non-diseased) tissue was used, as the HLCA core serves as the control/healthy reference. All other data was included in the extended HLCA.

Replication

No replication experiments were done. One of the goals of building the HLCA, i.e. pooling data across studies, is to enable checking reproducibility of findings across studies.

Randomization

There were no different experimental groups, such as treated versus control, in the newly generated HLCA data.

Blinding

As there were no different experimental groups, there was also no blinding.

Reporting for specific materials, systems and methods

We require information from authors about some types of materials, experimental systems and methods used in many studies. Here, indicate whether each material, system or method listed is relevant to your study. If you are not sure if a list item applies to your research, read the appropriate section before selecting a response.

Materials & experimental systems

n/a	Involved in the study
☒	☐ Antibodies
☒	☐ Eukaryotic cell lines
☒	☐ Palaeontology and archaeology
☒	☐ Animals and other organisms
☐	☒ Clinical data
☒	☐ Dual use research of concern

Methods

n/a	Involved in the study
☒	☐ ChIP-seq
☒	☐ Flow cytometry
☒	☐ MRI-based neuroimaging

Clinical data

Policy information about clinical studies

All manuscripts should comply with the ICMJE guidelines for publication of clinical research and a completed CONSORT checklist must be included with all submissions.

Clinical trial registration

NCT04529993

Study protocol

https://clinicaltrials.gov/ct2/show/study/NCT04529993

Data collection

Participants recruited by the Pneumology Unit of the Nice University Hospital were sampled between the 1st and the 15th of December 2020. The full procedure is detailed in https://www.clinicaltrials.gov/ct2/show/NCT04529993. Nasal and tracheobronchial samples were collected from IPF patients after obtention of their informed consent, following a protocol approved by CHU Nice. The data was derived from the clinical trial registered at clinicaltrials under reference NCT04529993. This study was described as an "interventional study" instead of an "observational study" because the participants were volunteers and all assigned to a specific bronchoscopy not related to routine medical care. Participants were prospectively assigned to a procedure (bronchoscopy) according to a specific protocol to assess our ability to sample the airway. No other procedures were included in this study. "Participants recruited by the Pneumology Unit of the Nice University Hospital were sampled between the 1st and the 15th of December 2020. The full procedure is detailed in https://www.clinicaltrials.gov/ct2/show/NCT04529993".

Outcomes

As this study did not involve any treatments are tracking of patients, just a single biopsy from volunteers, "outcomes" were not tracked in this study.
